# miR-181b targets semaphorin 3A to mediate TGF-β–induced endothelial-mesenchymal transition related to atrial fibrillation

**DOI:** 10.1172/JCI142548

**Published:** 2022-07-01

**Authors:** Ying-Ju Lai, Feng-Chun Tsai, Gwo-Jyh Chang, Shang-Hung Chang, Chung-Chi Huang, Wei-Jan Chen, Yung-Hsin Yeh

**Affiliations:** 1 Cardiovascular Department, Chang Gung Memorial Hospital, Tao Yuan, Taiwan.; 2 Department of Respiratory Therapy, Chang Gung University College of Medicine, Tao Yuan, Taiwan.; 3 Department of Respiratory Care, Chang Gung University of Science and Technology, Chia Yi, Taiwan.; 4 Department of Thoracic and Cardiovascular Surgery, Chang Gung Memorial Hospital, Tao Yuan, Taiwan.; 5 Department of Medicine and; 6 Graduate Institute of Clinical Medical Sciences, Chang Gung University College of Medicine, Tao Yuan, Taiwan.; 7 Department of Pulmonary and Critical Care Medicine, Chang Gung Memorial Hospital, Tao Yuan, Taiwan.

**Keywords:** Cardiology, Endothelial cells, Fibrosis, Heart failure

## Abstract

Atrial fibrosis is an essential contributor to atrial fibrillation (AF). It remains unclear whether atrial endocardial endothelial cells (AEECs) that undergo endothelial-mesenchymal transition (EndMT) are among the sources of atrial fibroblasts. We studied human atria, TGF-β–treated human AEECs, cardiac-specific TGF-β–transgenic mice, and heart failure rabbits to identify the underlying mechanism of EndMT in atrial fibrosis. Using isolated AEECs, we found that miR-181b was induced in TGF-β–treated AEECs, which decreased semaphorin 3A (Sema3A) and increased EndMT markers, and these effects could be reversed by a miR-181b antagomir. Experiments in which Sema3A was increased by a peptide or decreased by a siRNA in AEECs revealed a mechanistic link between Sema3A and LIM-kinase 1/phosphorylated cofilin (LIMK/p-cofilin) signaling and suggested that Sema3A is upstream of LIMK in regulating actin remodeling through p-cofilin. Administration of the miR-181b antagomir or recombinant Sema3A to TGF-β–transgenic mice evoked increased Sema3A, reduced EndMT markers, and significantly decreased atrial fibrosis and AF vulnerability. Our study provides a mechanistic link between the induction of EndMT by TGF-β via miR-181b/Sema3A/LIMK/p-cofilin signaling to atrial fibrosis. Blocking miR-181b and increasing Sema3A are potential strategies for AF therapeutic intervention.

## Graphical abstract

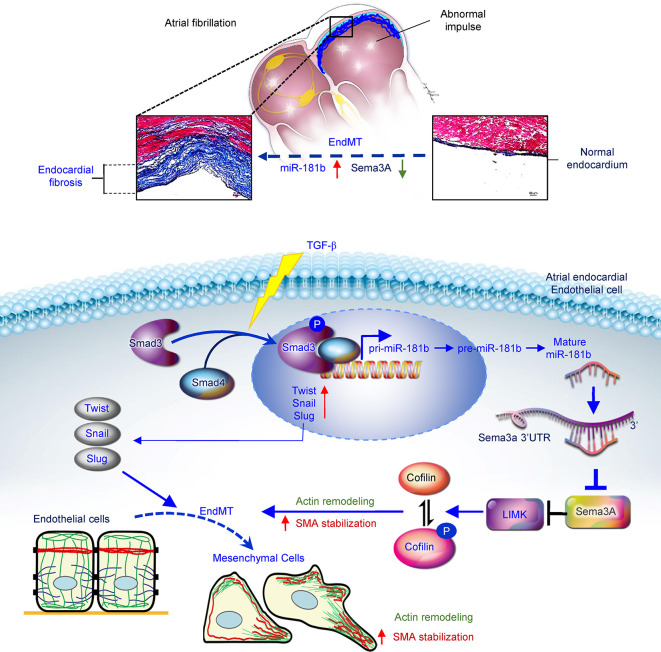

## Introduction

Atrial fibrillation (AF) is the most common sustained arrhythmia and results in increased morbidity and mortality. Rhythm control in persistent long-term AF, which is characterized by severe atrial myocyte remodeling and atrial fibrosis (1), is difficult to achieve with either antiarrhythmic drugs or catheter ablation. Atrial fibrosis contributes to the maintenance of AF and is among the important factors that make AF refractory to rhythm control (2, 3). Various stimuli provoke atrial fibrosis, including heart failure (HF), diabetes, vigorous exercise, and hypertension, by increasing the systemic or paracrine expression of profibrotic mediators, such as TGF-β (4). The well-characterized transcriptional program by which TGF-β induces epithelial-mesenchymal transition (EMT) is coordinated primarily by the SMAD2/3-dependent upregulation of the transcription factors Snail, Slug, and Twist (5, 6). In cardiac fibrosis, endothelial cells (ECs) can acquire a mesenchymal phenotype and express typical markers of myofibroblast differentiation, such as smooth muscle α actin (SMA), vimentin, and collagens (7). Moreover, the expression of SMA, a widely characterized cytoskeletal protein, is the hallmark of myofibroblast differentiation. TGF-β1 stimulates SMA expression and incorporation into stress fibers, thus increasing the myofibroblast contractile force in tissue remodeling (8, 9). Treatments for AF aim to prevent the formation and worsening of atrial fibrosis. TGF-β is an important mediator of atrial fibrosis in AF genesis (10). Previously, we showed that TGF-β causes atrial fibrosis mediated by oxidative stress in cardiac-specific TGF-β–transgenic mice (11).

Endothelial-mesenchymal transition (EndMT) is a transcriptional program that downregulates the expression of endothelial genes and upregulates the expression of mesenchymal genes. Through this process, ECs transdifferentiate into mesenchymal cells during embryogenesis and in several pathological conditions, such as cardiac fibroelastosis (12), venous graft remodeling (13), and pulmonary artery hypertension (14). Endocardial ECs can transdifferentiate into fibroblasts via EndMT and contribute to extracellular matrix deposition (7, 12, 15–19). Cardiac ECs can directly and indirectly influence cardiac function (1). Atrial endocardial endothelial cells (AEECs) line the inner surface of atria and may influence atrial function. The dysfunction of AEECs can lead to atrial thrombogenesis and increase the risk of embolic stroke (20). There is evidence for AEEC dysfunction resulting from reduced nitric oxide levels, upregulated prothrombotic factors, and downregulated antithrombotic factors (21). It was recently reported that AF is associated with EndMT in human atria (22). However, the underlying mechanism is unknown, and it is unclear whether EndMT contributes to atrial fibrosis and arrhythmogenesis in AF. In this study, we isolated AEECs and investigated how cells of the endocardial endothelium may transdifferentiate into mesenchymal cells via EndMT and contribute to atrial fibrosis.

## Results

### Subendocardial fibrosis is a marker of AF and HF.

**Figure 1:**
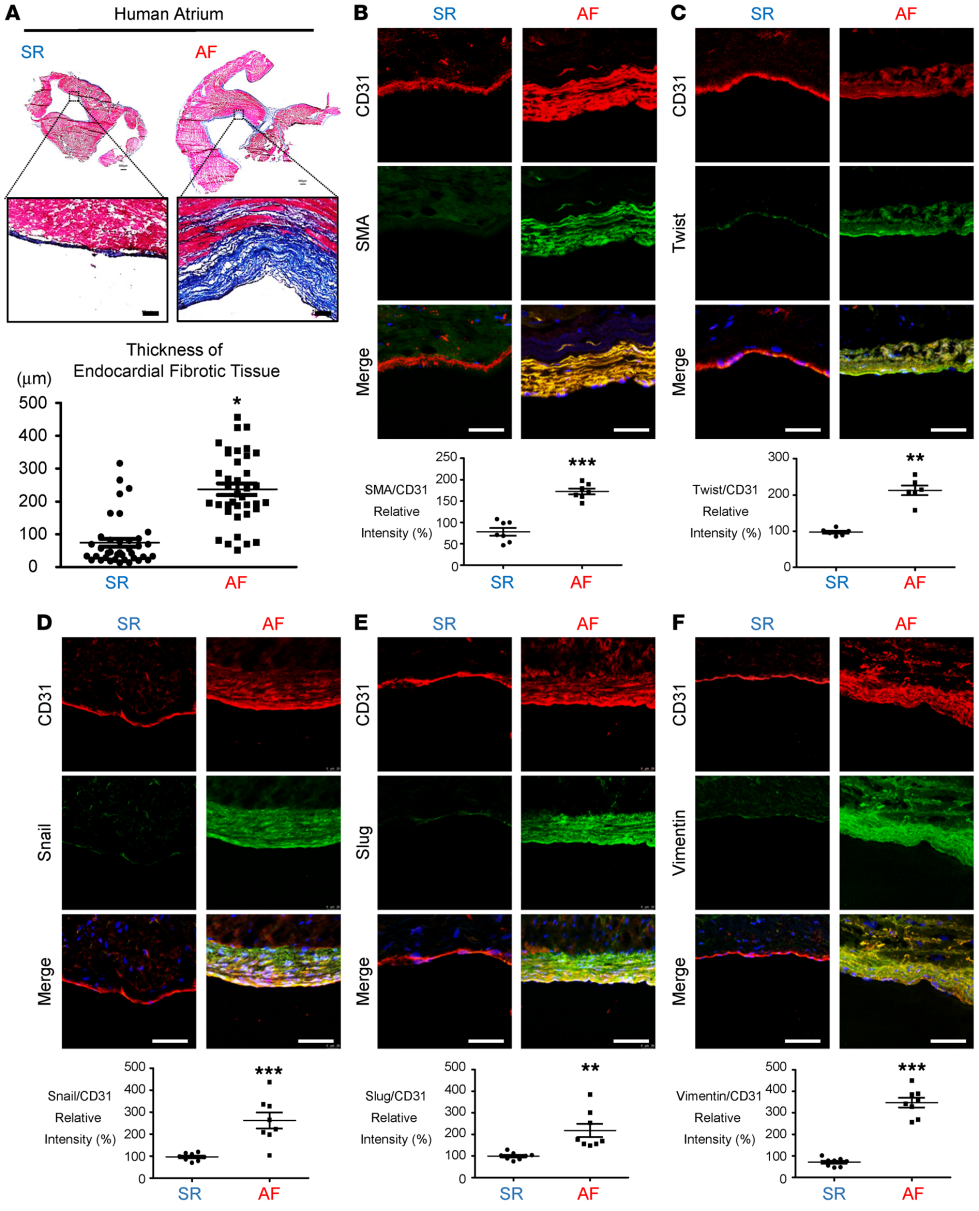
Histological analysis of atrial tissue. (**A**) Atrial appendage morphology and trichrome staining showed greater collagen (blue) deposition in endocardial tissue from patients with AF than in tissue from patients with SR. Scale bars: 100 μm. Original magnification: 500 μm. Plot shows quantitative analysis of endocardial fibrotic tissue thickness (*n =* 4). Immunohistochemical analysis of (**B**) SMA and CD31 and (**C**) Twist, (**D**) Snail, (**E**) Slug, (**F**) vimentin and CD31 in the endocardium layer. Scale bars: 50 μm. Quantitation of EndMT marker (CD31) expression in the endocardium (*n =* 8 per group). All data are presented as the mean ± SEM. (**A**–**F**) *P < 0.05, **P < 0.01, and ***P < 0.001 versus SR, by 2-tailed Student’s *t* test.

To investigate the subendocardial fibrosis patterns under normal and AF conditions, we examined atria from patients with chronic AF (Table 1) and found a significant increase in subendocardial fibrosis by trichrome staining compared with atria from patients with sinus rhythm (SR) (Figure 1A). We further established a rabbit model of HF using ventricular pacing as previously described (23). Consistently, subendocardial fibrosis was significantly thicker in the tachypacing-induced HF group than in the sham-treated group (Supplemental Figure 1A; supplemental material available online with this article; https://doi.org/10.1172/JCI142548DS1). CD31 (platelet–endothelial cell adhesion molecule [PECAM]) serves as an EC marker, whereas Twist, SMA, Snail, Slug, and vimentin are markers of TGF-β–induced EndMT (7, 13, 16, 23). We used double-IHC to evaluate subendocardial EndMT. Colocalization of CD31 with SMA, Twist, Snail, Slug, and vimentin was significantly increased in the atrial subendocardium of patients with AF (Figure 1, B–F), and increased colocalization of CD31 with SMA and Twist was observed in the HF rabbit model (Supplemental Figure 1, B and C). The coexpression of SMA, Twist Snail, Slug, and vimentin was significantly increased in AF atria, suggesting that EndMT may be involved in the development of subendocardial fibrosis.

### TGF-β induces miR-181b expression and reduces semaphorin 3A expression in human AEECs.

**Figure 2:**
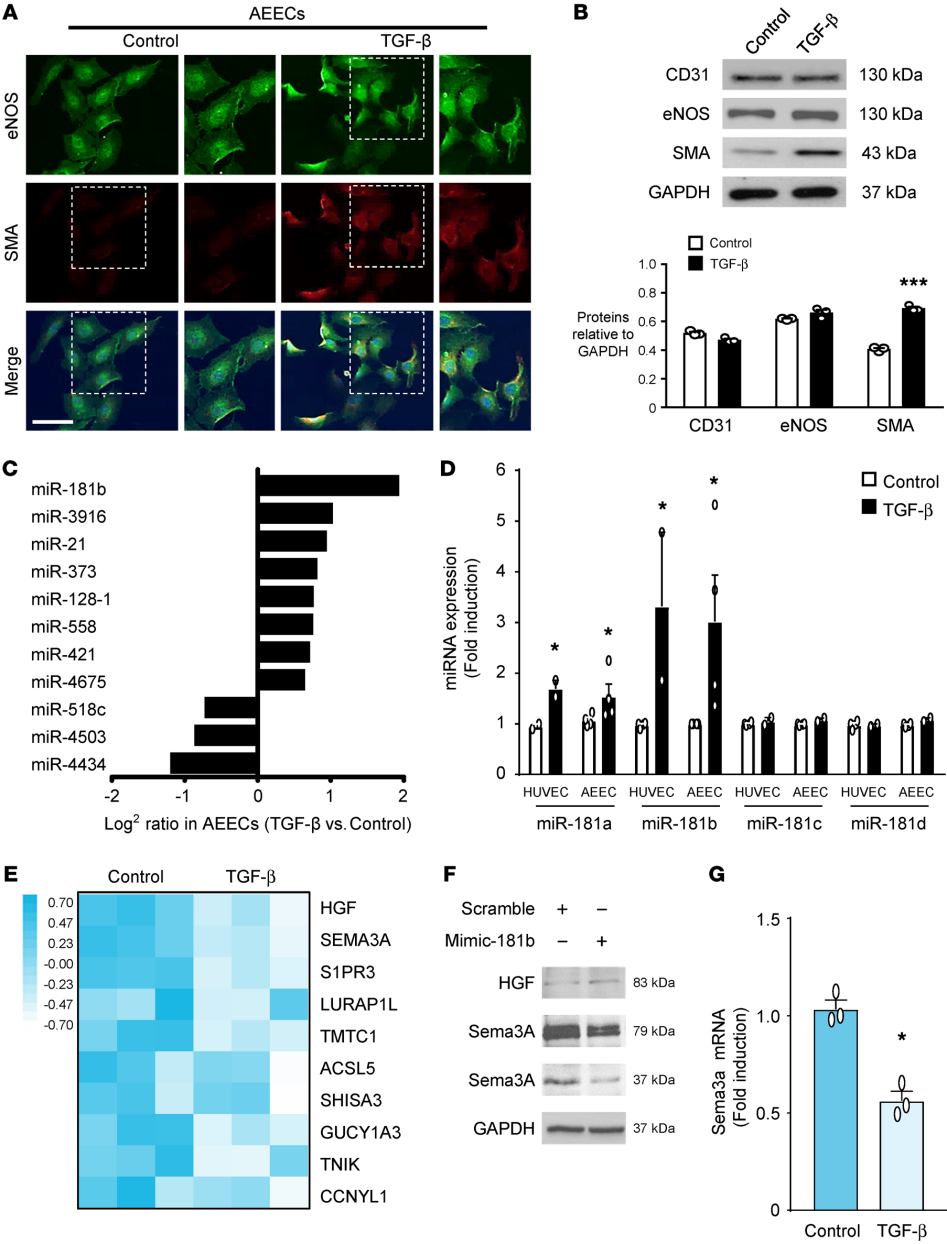
TGF-β induces EndMT and miR-181b expression and reduces Sema3A mRNA levels in human AEECs. (**A**) Localization of SMA (red) and eNOS (green) (nuclear staining with DAPI is shown in blue). Scale bar: 75 μm. (**B**) Quantitative analysis with normalization to GAPDH. ***P < 0.001 versus the untreated group, by 2-tailed Student’s *t* test (*n =* 3). Western blotting shows the protein expression levels in AEECs treated with or without TGF-β 5ng/ml. (**C**) miR-181b expression was significantly higher in AEECs treated with TGF-β. (**D**) miR-181 gene profiles, as determined by qRT-PCR, with U6 used as the loading control (*n =* 3). The white bars represent ECs without TGF-β treatment, and the black bars represent cells treated with TGF-β. *P < 0.05 versus the control group, by 2-tailed Student’s *t* test. (**E**) Heatmap shows the top 10 genes downregulated in response to TGF-β and related to miR-181b with a minimum change of 1.5-fold and *P* < 0.05, by 2-tailed Student’s *t* test between the control and TGF-β groups. (**F**) AEECs were transfected with the miR-181b mimic (mimic-181b) or the scrambled control. Representative immunoblot of HGF and Sema3A expression. (**G**) Representative Sema3A mRNA levels with GAPDH used as the loading control. *n =* 3. *P < 0.05 versus the untreated group, by 2-tailed Student’s *t* test.

We prepared primary cultures of AEECs from human atrial appendages to elucidate the mechanism of TGF-β–induced EndMT and subendocardial fibrosis. The protocol for AEEC isolation is shown in Supplemental Figure 2A. We used the EC-specific markers CD31 and eNOS to characterize the AEECs (Supplemental Figure 2B). CD31 and eNOS were downregulated at passage 4 (Supplemental Figure 2C), so we used AEECs before passage 4 in subsequent experiments. Immunocytochemical staining showed that SMA was upregulated in TGF-β–treated AEECs (Figure 2A). Western blot analysis revealed that TGF-β treatment of AEECs increased SMA expression, but CD31 and eNOS levels did not change significantly (Figure 2B). Since eNOS is also expressed in nonendothelial tissues, such as cardiac myocytes (5, 24), CD31 was used as an EC-specific marker throughout this study. We then performed Human Transcriptome Array 2.0 and principal component analysis (PCA) (Supplemental Figure 3A) to screen for genes with altered expression profiles in AEECs treated with or without TGF-β. This analysis identified miR-181b as the most upregulated miRNA in TGF-β–treated AEECs (Figure 2C). Human umbilical vein endothelial cells (HUVECs) have been used as an in vitro model of EndMT (25), so we compared the responses of AEECs and HUVECs to TGF-β and verified the upregulation of miR-181a and miR-181b by quantitative real-time PCR (qRT-PCR) (Figure 2D). With the gene list from the array data (Supplemental Table 1), we explored candidate genes targeted by miR-181b with a miRNA target prediction program (TargetScan algorithm) and predicted potential miR-181b binding sites in the 3′-UTR of the top 10 genes downregulated in response to TGF-β and related to miR-181b. ; these 10 genes are labeled in the heatmap in Figure 2E, and the fold change in expression of these genes is shown in Supplemental Figure 3B. Of these 10 genes, we selected semaphorin 3A (Sema3A) as the most promising potential target of miR-181b in mediating EndMT. Transfection of miR-181b mimics (mimic-181b) into AEECs decreased Sema3A expression but not hepatocyte growth factor (HGF) compared with transfection of scrambled mimics (Figure 2F). Using bioinformatics matching analysis, we predicted that miR-181b-3p (7 nt) binds to the conserved sequence 5′-UCAGUGA-3′ (positions 6368–6374 in the human sequence) in the 3′-UTR of Sema3A mRNA (Supplemental Figure 3C). Consistently, qRT-PCR confirmed that Sema3A mRNA was downregulated in response to TGF-β (Figure 2G), suggesting that miR-181b upregulation and Sema3A downregulation might contribute to TGF-β–induced EndMT in AEECs.

### TGF-β induces miR-181b expression via SMAD2/3 signaling.

**Figure 3:**
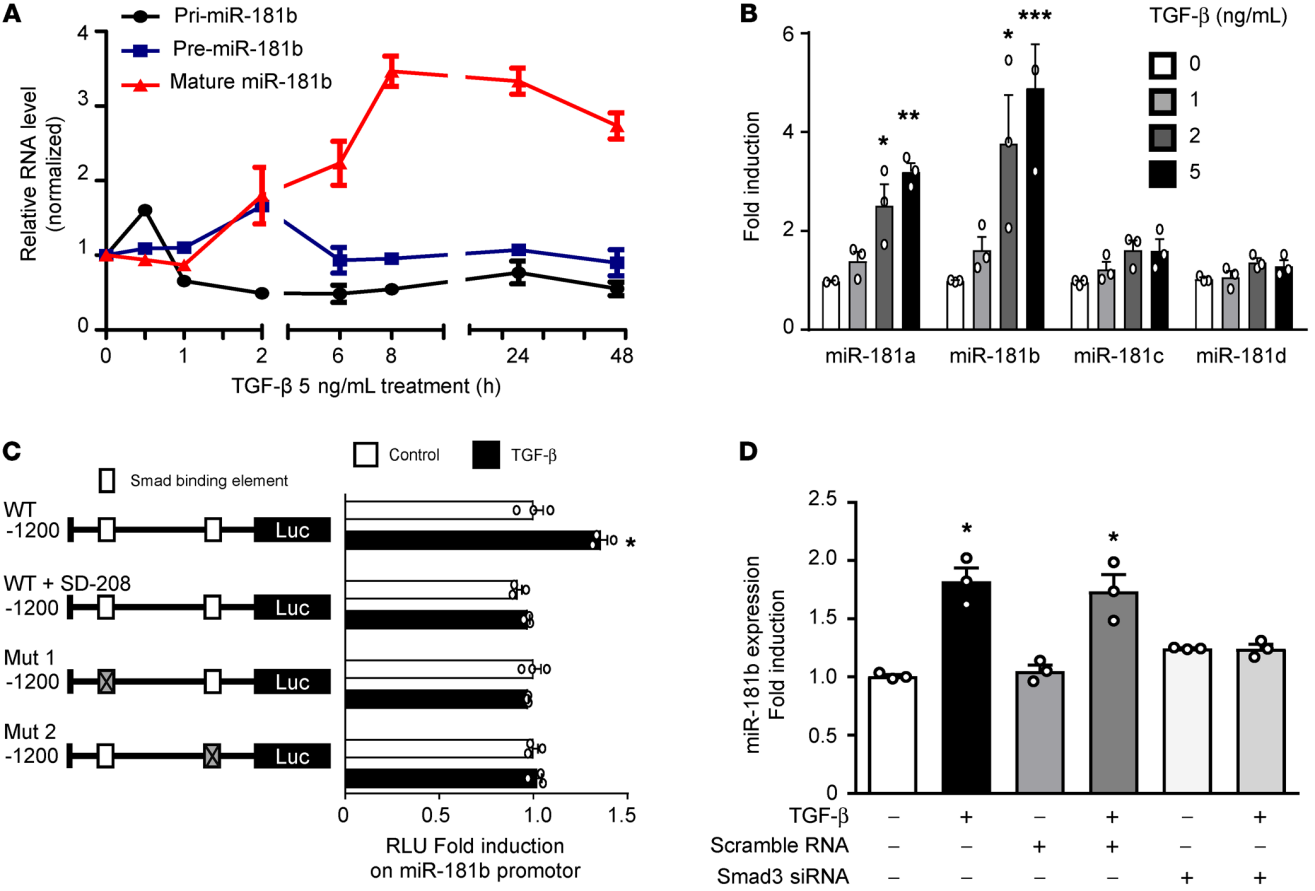
TGF-β induces miR-181b via SMAD2/3 signaling in vitro. (**A**) qRT-PCR analysis of mature (miR-181b), precursor (Pre-miR-181b), and primary (Pri-miR-181b) transcripts of miR-181b in AEECs treated with TGF-β for 0–48 hours (*n =* 3). (**B**) miR-181 RNA expression in AEECs treated with or without TGF-β. 18S rRNA served as the loading control. (**C**) Left: Linear map schematic of the putative SBEs at the promoter of the miR-181b gene, with the mutated luciferase (Luc) constructs. Right: Graph shows the luciferase assay results. AEECs were transiently transfected with mutant promoter constructs and treated with or without 5 ng/mL TGF-β for 8 hours. miR-181b promoter characterization by luciferase assays showed the fold change in luciferase activity of the WT SBEs and mutated SBE1 and SBE2 (Mut 1 and Mut 2; left) with and without 5 ng/mL TGF-β or 10 μM SD-208. (**D**) Representative qRT-PCR analysis of miR-181b in AEECs with siRNA-mediated SMAD3 knockdown and TGF-β treatment. (**A**, **B**, and **D**) *P < 0.05, **P < 0.01, and ***P < 0.001 versus the untreated group, by 1-way ANOVA with Dunnett’s post hoc test (*n =* 3). (**C**) *P < 0.05, by 2-tailed Student’s *t* test (*n =* 3).

Studies have demonstrated abnormal miRNA expression in cardiac fibrosis development and EndMT progression (12, 26). TGF-β controls gene expression through SMAD proteins, which are known signal transducers and transcriptional modulators (27) but have not been shown to affect RNA processing. To evaluate whether TGF-β affects miR-181b expression at the transcriptional level, we examined the primary miR-181b gene transcript (pri-miR-181b), pre-miR-181b, and mature miR-181b after TGF-β treatment over time to observe miR-181b biogenesis in AEECs. We observed the induction of pri-mir-181b at 0.5 hours, pre-mir-181b at 2 hours, and mature miR-181b between 2 hours and 24 hours after TGF-β treatment (Figure 3A). In AEECs, TGF-β treatment increased miR-181b expression in a time- and concentration-dependent manner (Figure 3, A and B), suggesting that the induction of miR-181b by TGF-β occurs at the transcriptional level. We then assessed whether SMAD3, a key TGF-β mediator (27), has a direct effect on the upregulation of miR-181b by TGF-β. To begin, we subcloned a plasmid to monitor the TGF-β–driven transactivation of a miR-181b–specific reporter (MLP-luc). Bioinformatic analysis identified 2 putative SMAD3-binding elements (SBEs) (–1091 to –1085 and –404 to –399) (11) in the miR-181b promoter region. Mutational analyses confirmed that miR-181b transcription induced by TGF-β/SMAD signaling was evident only when the promoter constructs contained SBE1 and SBE2 (Figure 3C). Transient transfection studies showed increased miR-181b promoter activity in TGF-β–treated AEECs, and this effect was reversed by SD-208 (a TGF-β/SMAD3 signaling inhibitor; Figure 3C). Furthermore, siRNA-mediated SMAD3 knockdown blocked TGF-β–induced miR-181b transcription (Figure 3D), indicating crucial roles for miR-181b and a SMAD3-dependent pathway in the effects of TGF-β. The knockdown efficiency of SMAD3 siRNA was confirmed by decreased SMAD3 expression in siRNA-transfected AEECs (Supplemental Figure 4). These results demonstrated that TGF-β/SMAD signaling mediates miR-181b transcriptional activity and may be involved in EndMT progression.

### miR-181b targets Sema3A to mediate TGF-β–induced EndMT.

**Figure 4:**
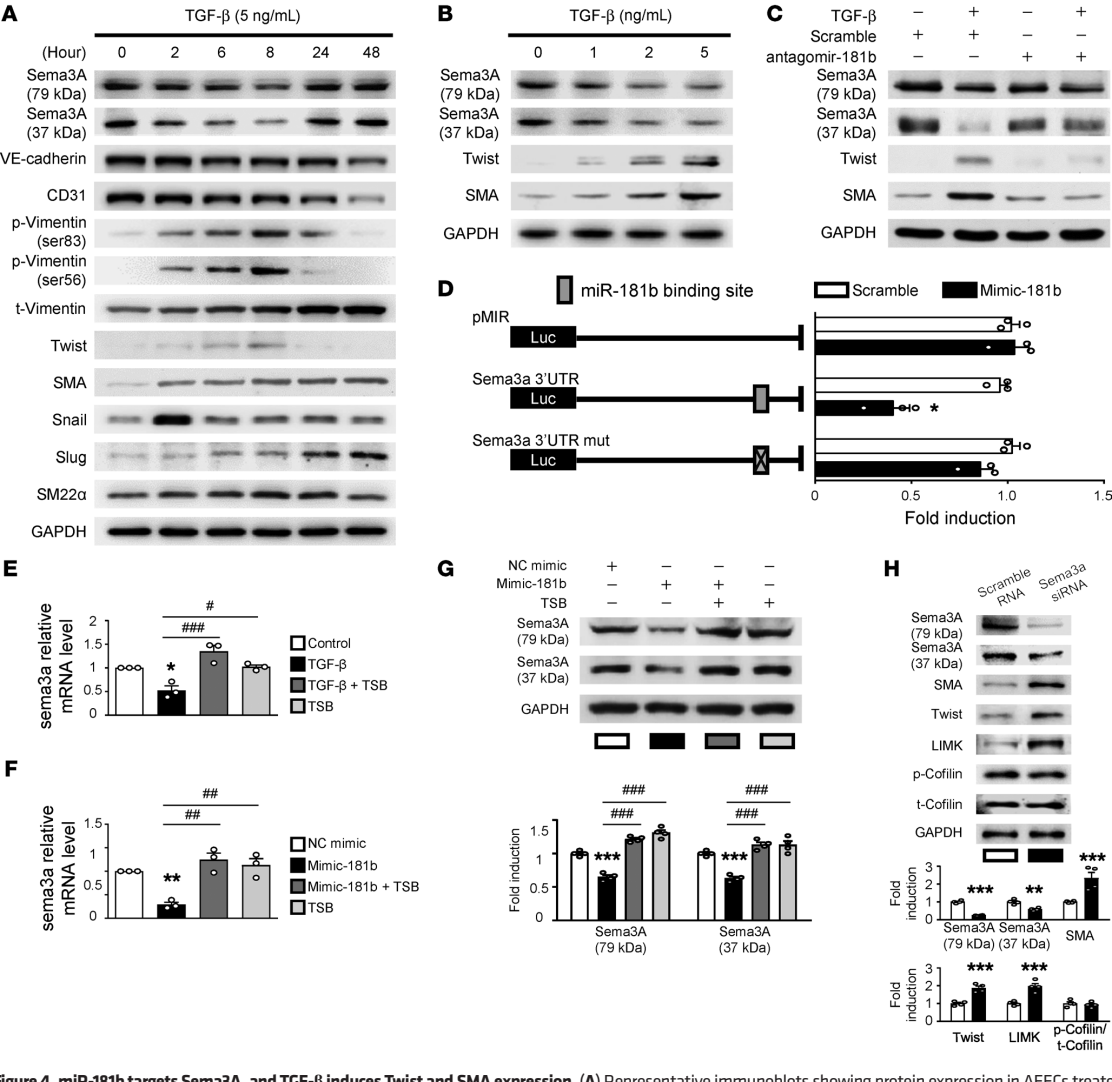
miR-181b targets Sema3A, and TGF-β induces Twist and SMA expression. (**A**) Representative immunoblots showing protein expression in AEECs treated with 5 ng/mL TGF-β for up to 48 hours. GAPDH was used as the loading control. (**B**) Representative immunoblots showing protein expression in AEECs incubated with or without TGF-β (0, 1, 2, or 5 ng/mL). (**C**) Representative immunoblots show protein expression in AEECs that were transfected with miR-181b antagomir (antagomir-181b) or scrambled control miRNA and then treated with or without TGF-β (5 ng/mL). (**D**) Confirmation of the hsa-miR-181b target site in the Sema3A 3′-UTR. Schematic representation of the Sema3A 3′-UTR indicating the predicted hsa-miR-181b binding site. AEECs were transfected with the pMIR-REPORT-Sema3A 3′-UTR (intact) or pMIR-REPORT-Sema3A 3′-UTR (mutant) luciferase reporter vector. The fold induction in relative luciferase activity was plotted (*n =* 3). *P < 0.05 compared with the scrambled control, by 2-tailed Student’s *t* test. (**E**) AEECs were transfected with the miR-181b TSB for 24 hours, after which AEECs were treated with or without TGF-β for 6 hours. Sema3A mRNA expression was measured by qRT-PCR (*n* = 3). *P < 0.05 compared with control; ^#^*P <* 0.05 and ^###^*P <* 0.001 compared with TGF-β; 1-way ANOVA with Bonferroni’s post hoc test. (**F**) AEECs were transfected with the negative control mimic (NC mimic) or the synthetic miR-181b mimic, with or without the miR-181b TSB. Sema3A mRNA expression was measured by qRT-PCR (*n =* 3). **P < 0.01 compared with NC mimic; ^##^*P <* 0.01 compared with miR-181b mimic; 1-way ANOVA with Bonferroni’s post hoc test. (**G**) Representative quantitative data for the immunoblots showing protein expression in AEECs transfected with miR-181b TSBs for 24 hours (*n =* 3). ***P < 0.001 versus the NC mimic group; ^###^*P <* 0.001 versus the 181b mimic group; 1-way ANOVA with Bonferroni’s post hoc test. (**H**) AEECs were transfected with Sema3A siRNA or scrambled siRNA. Representative immunoblots and densitometric quantification of protein expression are shown (*n =* 3). **P < 0.01 and ***P < 0.001 compared with scrambled siRNA–treated group, by 2-tailed Student’s *t* test. t, total.

TGF-β–induced EndMT, which is involved in cardiac fibrosis (2, 7, 28), is a complex process, whereby ECs adopt a mesenchymal phenotype and express mesenchymal cell markers, such as SMA and Twist (28); however, this process has not, to our knowledge, been evaluated in AEECs. We first evaluated whether miR-181b and Sema3A, a secreted glycoprotein that is crucial for embryonic heart development (29), are associated with TGF-β–induced EndMT. Time-course experiments in TGF-β–treated AEECs revealed that Sema3A levels decreased beginning at 2 hours, SMA levels increased from 2 hours to 48 hours, Twist levels increased from 2 hours to 8 hours, Snail levels increased to a peak at 2 hours, p-vimentin (Ser56 and Ser83) levels increased from 2 hours to 8 hours, and then we observed that mRNA and protein levels of vimentin and Slug increased from 24 hours to 48 hours (Figure 4A and Supplemental Figure 5, A and B). Furthermore, TGF-β treatment increased the expression of mesenchymal markers (SMA and Twist) and decreased Sema3A expression in a somewhat concentration-dependent manner (Figure 4B and Supplemental Figure 5C). In subsequent experiments, we evaluated relative protein expression levels in cells treated with 5 ng/mL TGF-β for 8 hours. Full-length Sema3A is initially synthesized as an inactive precursor of approximately 110 kDa that is processed into functional N-terminal and C-terminal fragments of 79 kDa and 37 kDa, respectively (30). Importantly, the 37 kDa fragment is less abundant than the 79 kDa fragment (30). Interestingly, when cells were treated with TGF-β, Western blotting showed that expression of both the 37 kDa and 79 kDa forms of Sema3A was significantly decreased. To further confirm the role of miR-181b/Sema3A in TGF-β–induced EndMT, we performed loss-of-function experiments using a miR-181b antagomir (antagomir-181b). Transfection of antagomir-181b into AEECs rescued Sema3A expression and prevented the upregulation of SMA and Twist in response to TGF-β (Figure 4C and Supplemental Figure 5D). We next subcloned luciferase reporter plasmids containing the WT Sema3A 3′-UTR, which harbors potential miR-181b–binding sites, or a version with mutated miR-181b–binding sites, to determine whether Sema3A is a direct target of miR-181b. We found that luciferase activity was significantly decreased in mimic-181b–transfected AEECs compared with scramble control–transfected cells, but this effect was reversed in the presence of the mutated Sema3A 3′-UTR (Figure 4D). To further confirm the direct effect of miR-181b on Sema3A, we performed target site blocker (TSB) experiments using custom-designed locked nucleic acid phosphorothioate oligonucleotides (QIAGEN) that bind to the Sema3A 3′-UTR to prevent miR-181b binding (Figure 4, E–G). Transfection of the TSB into AEECs specifically rescued the reduction in Sema3A mRNA and protein expression induced by treatment with TGF-β (Figure 4E) or the miR-181 mimic (Figure 4, F and G). Together, these data suggest that miR-181b is upregulated by TGF-β and then directly targets Sema3A mRNA, leading to decreased Sema3A protein levels in AEECs. In addition, Sema3A affects the cytoskeleton and cancer cell survival by negatively regulating LIMK/p-cofilin pathway–dependent actin polymerization (31). In loss-of-function experiments, we showed that siRNA-mediated Sema3A knockdown induced the expression of SMA, Twist, and LIMK in AEECs (Figure 4H). These results provide further evidence that Sema3A is directly targeted by miR-181b to regulate the LIMK/p-cofilin/SMA/Twist axis and may play a causative role in the progression of TGF-β–induced atrial EndMT.

### Sema3A regulates EndMT through LIMK/p-cofilin signaling and actin remodeling during lamellipodia formation.

**Figure 5:**
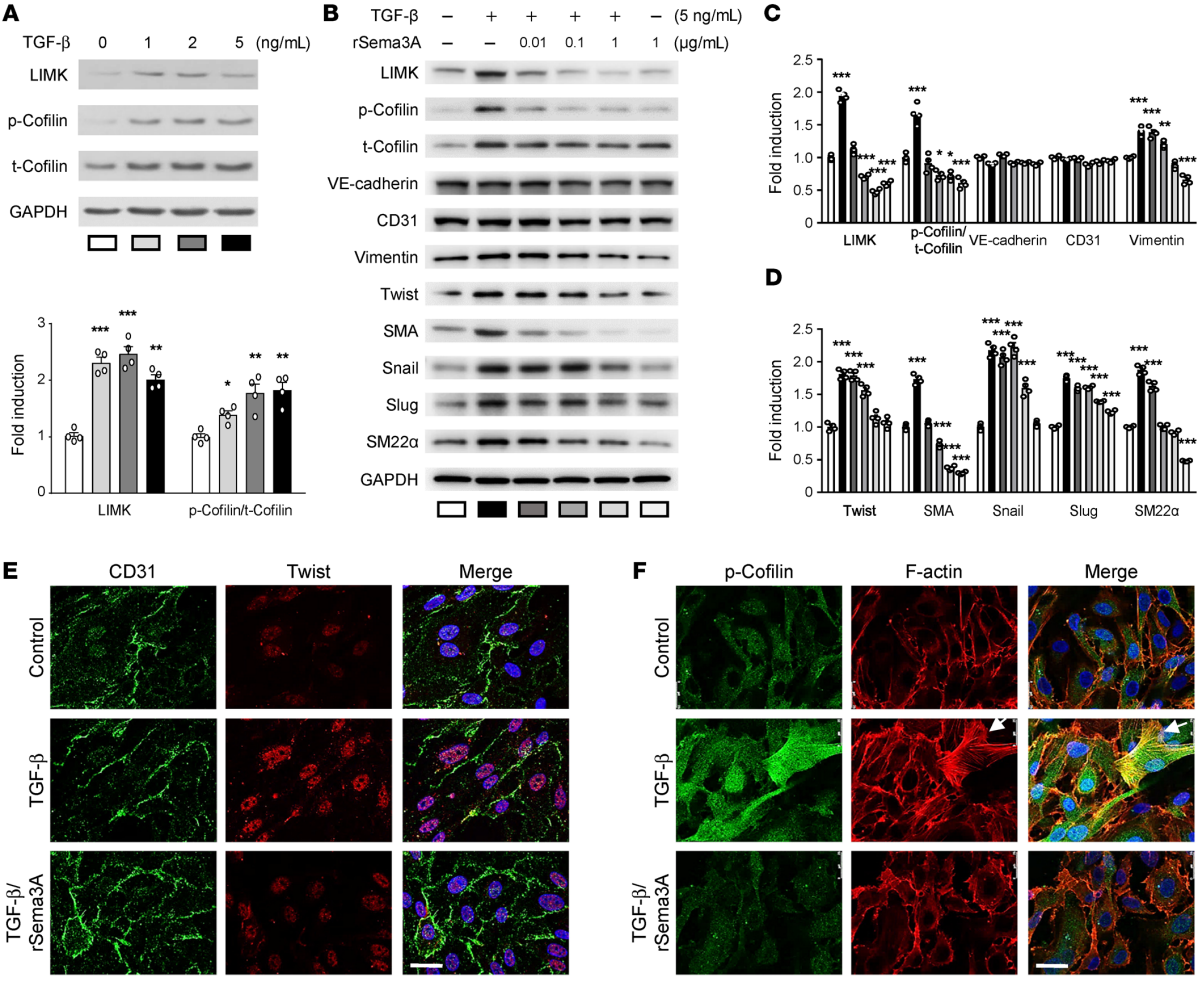
The effect of recombinant Sema3A on TGF-β–mediated induction of LIMK/p-cofilin signaling and EndMT. (**A**) Immunoblots showing protein expression levels in AEECs treated with 0, 0.01, 0.1, or 1 ng/mL TGF-β with GAPDH as the loading control (*n =* 4) and densitometric quantification of protein expression following TGF-β treatment. *P < 0.05, **P < 0.01, and ***P < 0.001 versus the untreated group, by 1-way ANOVA with Dunnett’s post hoc test. (**B**–**D**) Representative immunoblots (**B**) and densitometric quantification (**C** and **D**) of protein expression. Data are presented as the mean ± SEM (*n =* 3). *P < 0.05, **P < 0.01, and ***P < 0.001 versus the untreated group, by 1-way ANOVA with Dunnett’s post hoc test. (**E**) Immunocytochemical analysis shows the localization of CD31 (green) and Twist (red). Scale bar: 25 μm. (**F**) Immunocytochemical analysis shows the localization of p-cofilin (green) and F-actin (red) (nuclear staining with DAPI is shown in blue). White arrows indicate lamellipodia and filopodia. Scale bar: 25 μm.

The semaphorin family, including Sema3A, was shown in previous studies to mediate EMT (32). LIMK and cofilin are factors downstream of Sema3A (33). The LIMK/p-cofilin signaling pathway links the limited Sema3A signal to lamellipodia formation and mediates EMT via cytoskeletal actin remodeling in cancer cells (34–36). Furthermore, EndMT has been observed in the atrial tissue of patients with AF (22), but no detailed mechanism has been reported. Therefore, we hypothesized that the mechanistic link between the TGF-β–mediated decrease in Sema3A expression and the increase in LIMK/p-cofilin signaling plays an important role in EndMT associated with the pathogenesis of AF. The next experiments were designed to investigate whether LIMK/p-cofilin signaling, which is essential for cytoskeleton remodeling and filopodia formation, is influenced by TGF-β. Western blotting showed that TGF-β increased LIMK and p-cofilin levels in a concentration-dependent manner (Figure 5A). To determine whether Sema3A treatment can block LIMK/p-cofilin pathway–mediated actin assembly, we evaluated the effects of 0.01, 0.1, and 1 μg/mL recombinant Sema3A (rSema3A) on AEECs (Figure 5, B–D). Western blot analysis showed that TGF-β upregulated LIMK, p-cofilin, vimentin, Twist, SMA, Snail, Slug, and SM22α expression (Figure 5, B–D). Cotreatment of AEECs with 1 μg/mL rSema3A and 5 ng/mL TGF-β reduced LIMK, p-cofilin SMA, Twist, vimentin, Snail, Slug, and SM22α levels compared with treatment with TGF-β alone. Actin remodeling is essential during EndMT (37–39), in which cells acquire motility and invasive capabilities by developing actin-rich projections such as lamellipodia and filopodia (19). Immunocytochemistry showed that TGF-β increased Twist and p-cofilin levels and induced morphological protrusions of the cell membrane, indicating lamellipodia formation that was further supported by the detection of F-actin, thus providing evidence for actin remodeling and reorganization in AEECs (Figure 5, E and F). Furthermore, treatment with 1 μg/mL rSema3A inhibited the effects of TGF-β on Twist and p-cofilin expression to prevent lamellipodia formation (Figure 5, E and F). These data indicate that the inhibitory effect of rSema3A on Twist and actin remodeling is potentially mediated by LIMK/p-cofilin signaling, thus highlighting the crucial role of Sema3A in blocking TGF-β–induced EndMT via actin remodeling.

### Transgenic mice with cardiac-specific TGF-β overexpression develop atrial subendocardial fibrosis and AF.

**Figure 6:**
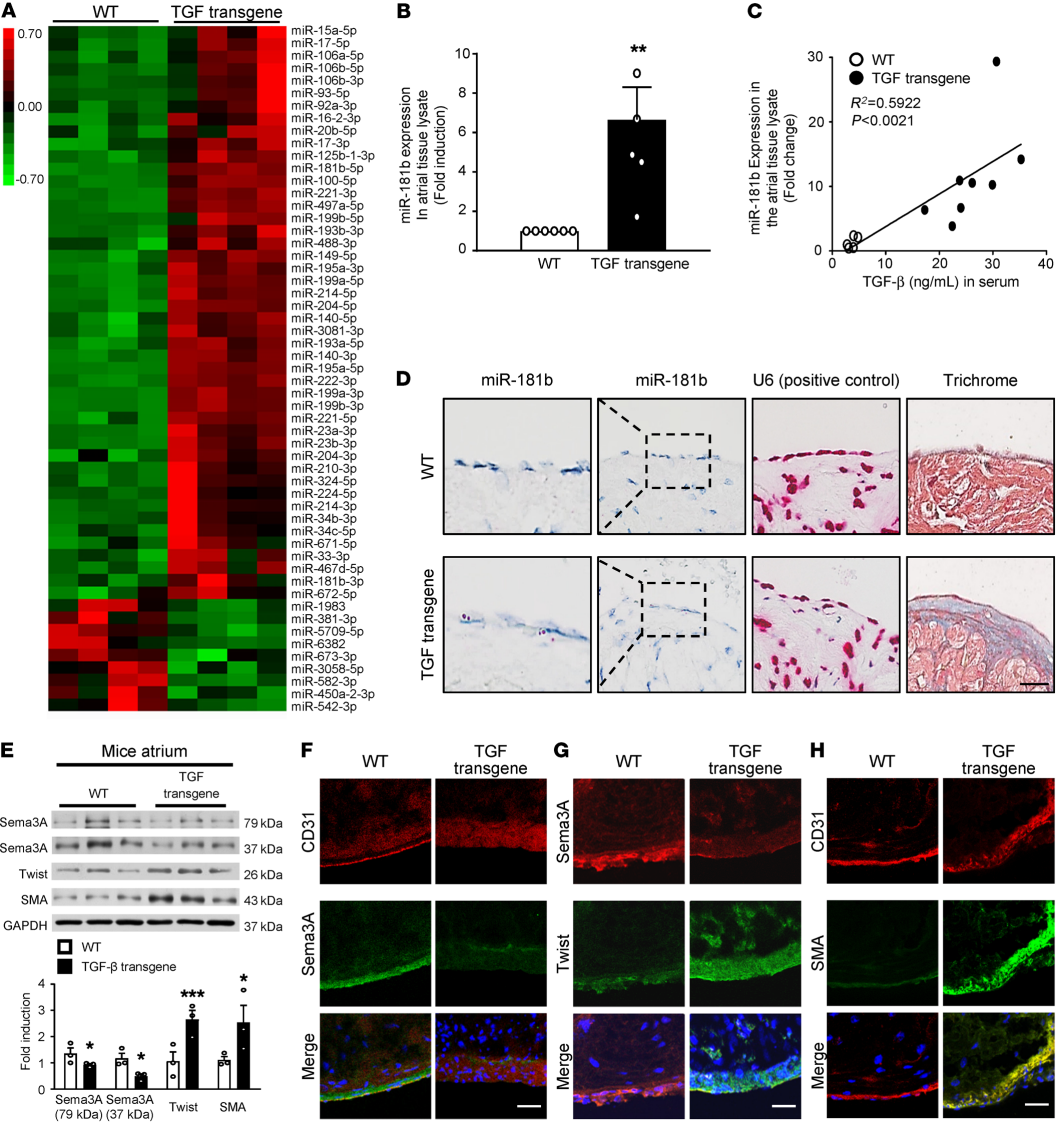
miR-181b and Sema3A expression in transgenic mice with cardiac-specific TGF-β overexpression. (**A**) Heatmap showing normalized read counts of miRNAs differentially expressed (>2-fold difference and *P* < 0.05, by 2-tailed Student’s *t* test) between WT and transgenic mice with cardiac-specific TGF-β overexpression (TGF transgene). Hierarchical clustering of samples is shown. Color bar indicates *z* scores of normalized read counts (red indicates high expression; green indicates low expression). (**B**) Validation of miR-181b expression using qRT-PCR in atrial tissue lysates with U6 as the loading control (*n =* 5–8). **P < 0.01 versus WT mice, by 2-tailed Student’s *t* test. (**C**) Correlation of miR-181b RNA levels from atrial tissue with serum TGF-β concentration by linear regression (*n =* 5–10). (**D**) RNA CISH analyses of miR-181b in atrial tissue. miR-181b signal (red dots) and U6 (used as a positive control, red dots) were observed in atrial endothelial edge tissue from TGF-β–transgenic mice, and trichrome staining shows miR-181b expression in atrial subendocardial fibrotic tissue (*n =* 5). Scale bar: 50 μm. Original magnification x400; enlarged insets x800. (**E**) Western blot analysis of Sema3A, Twist, and SMA in atrial tissue from TGF-β–transgenic and WT mice. The relative protein expression levels of Sema3A, Twist, and SMA, normalized to GAPDH, were obtained by densitometry (*n* = 3 per group). *P < 0.05 and ***P < 0.001 versus WT mice, by 2-tailed Student’s *t* test. (**F**–**H**) Immunohistochemical analysis of (**F**) Sema3A and CD31, (**G**) Sema3A and Twist, and (**H**) CD31 and SMA in the endocardium. Scale bars: 50 μm.

TGF-β signaling plays an essential role in atrial fibrosis (2, 28). To ascertain the miRNA expression patterns in cardiac-specific TGF-β–transgenic mice, we conducted small RNA-Seq of atrial whole-tissue lysates. Differential expression analysis of the mapped miRNAs yielded 55 miRNAs that were significantly differentially regulated between WT mice and TGF-β–transgenic mice (*P <* 0.05; –2 < fold change > 2) (Figure 6A), and the detailed sequencing summary of the small RNA libraries in shown in Supplemental Table 2, with the PCA shown in Supplemental Figure 6A. Among these 55 differentially regulated miRNAs, 46 were found to be upregulated and 9 were downregulated in TGF-β–transgenic mice. The heatmap showed that miR-181b was substantially upregulated in TGF-β–transgenic mice compared with WT mice but was not the most highly expressed miRNA (Figure 6A). This finding may relate to the fact that the atrial tissues used for miRNA analysis contained only 12.2% ECs (40). We then validated miR-181b expression in atrial tissue lysates from TGF-β–transgenic mice by qRT-PCR. As shown in Figure 6B, miR-181b expression was significantly increased in atria from TGF-β–transgenic mice, and miR-181b levels in atrial tissue were positively correlated with the TGF-β serum concentration (*P <* 0.002) (Figure 6C). To localize miR-181b expression in the atrial endocardium, we performed a specific RNA chromogenic in situ hybridization (CISH) assay for miR-181b with atrial tissue sections from TGF-β–transgenic mice and WT mice. Interestingly, we found that miR-181b was expressed at the edge of the atrial endothelium in TGF-β–transgenic mice and confirmed the location of miR-181b expression in atrial subendocardial fibrosis (Figure 6D). We evaluated the expression of EndMT markers in TGF-β–transgenic mice. Consistent with the in vitro findings, Twist and SMA expression levels were significantly increased in atria from TGF-β–transgenic mice compared with levels in atria from WT mice, whereas Sema3A expression was decreased (Figure 6, E–H; for quantification of the immunofluorescence results, see Supplemental Figure 7, A–C). Moreover, subendocardial fibrosis and AF inducibility were significantly increased in the transgenic mice (Supplemental Figure 6, B and C). These analyses of the atrial endocardium from TGF-β–transgenic mice compared with WT mice highlighted the critical role of Sema3A targeting by miR-181b in TGF-β–induced EndMT and the development of subendocardial fibrosis.

### Blocking miR-181b and increasing Sema3A reverse atrial subendocardial fibrosis, reduce EndMT markers, and decrease AF vulnerability in TGF-β–transgenic mice.

**Figure 7:**
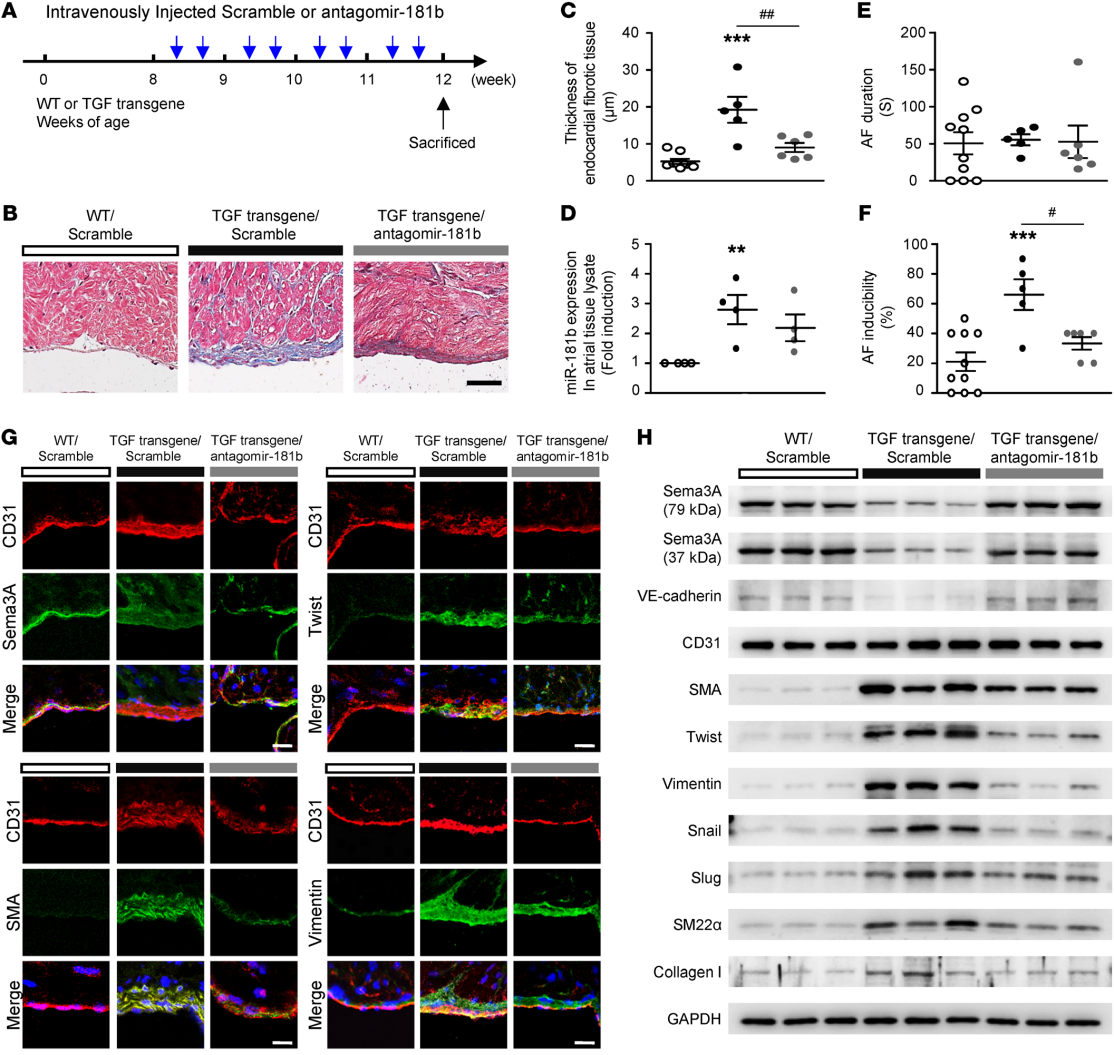
Antagomir-181b inhibits the development of atrial subendocardial fibrosis in TGF-β–transgenic mice. (**A**) Design and optimization of the appropriate treatment strategy. (**B**) Histological morphology analysis of trichrome-stained atrial tissue demonstrating collagen (blue) deposition. Scale bar: 25 μm. (**C**) Quantitative analysis of endocardial fibrotic tissue thickness, (**D**) miR-181b expression in atrial tissue lysates, (**E**) AF duration, and (**F**) AF inducibility. (**C**–**F**) Data are presented as the mean ± SEM (*n =* 5–7 per group). **P < 0.01 and ***P < 0.001 versus WT mice; ^#^*P <* 0.05 and ^##^*P <* 0.01, versus TGF-β–transgenic mice; 1-way ANOVA with Bonferroni’s post hoc test. (**G**) Immunohistochemical analysis of CD31 with Sema3A, Twist, SMA and vimentin in the endocardium (*n =* 5). Scale bars: 50 μm. (**H**) Western blot analysis of proteins in atrial tissue from WT, TGF-β–transgenic mice (TGF transgene), and TGF-β–transgenic mice with antagomir-181b treatment showing increased SMA, Twist, vimentin, Snail, Slug, SM22α, and collagen I levels but decreased Sema3A and VE-cadherin levels in TGF-β–transgenic mice compared with WT mice. TGF-β–transgenic mice with antagomir-181b treatment showed a reversal of Sema3A and VE-cadherin protein levels and reduced expression of EndMT markers.

After showing that miR-181b targeted Sema3A to mediate TGF-β–induced EndMT and atrial subendocardial fibrosis in vitro and in vivo, we aimed to evaluate whether miR-181b or Sema3A is a potential therapeutic target for reducing atrial fibrosis and AF vulnerability. TGF-β–transgenic mice were treated with 3 μL (1 nmol/μL) antagomir-181b intravenously twice a week for 4 weeks (Figure 7A). Treatment with antagomir-181b reduced the degree of atrial fibrosis in TGF-β–transgenic mice compared with scramble control treatment (Figure 7B). Cardiac morphology in longitudinal sections from mice in the 3 groups and histological analysis of trichrome-stained atrial tissue demonstrated a difference in the amount of collagen deposition between the antagomir-181b– and scramble control–treated groups (Figure 7B). Quantification of subendocardial fibrosis thickness revealed significant differences among the 3 groups (Figure 7C). Notably, the increased miR-181b expression in TGF-β–transgenic mice was reversed by antagomir-181b treatment (Figure 7D). TGF-β–transgenic mice exhibited increased AF inducibility, but not duration, compared with WT littermates (Figure 7, E and F). Moreover, antagomir-181b treatment significantly reduced AF inducibility in TGF-β–transgenic mice compared with scramble control treatment (Figure 7, E and F). The TGF-β–transgenic mice were subjected to burst atrial pacing via a transesophageal approach (Supplemental Figure 8). Accordingly, we determined whether antagomir-181b treatment is effective at preventing EndMT in atria of TGF-β–transgenic mice. Antagomir-181b treatment reduced EndMT marker levels and reversed Sema3A protein levels, as detected by IHC (EndMT marker localization) and Western blotting (quantification of protein expression) (Figure 7, G and H, and Supplemental Figure 9). To further investigate the potential therapeutic effect of rSema3A on TGF-β–induced AF, we intravenously injected TGF-β–transgenic mice with rSema3A (1 mg/kg; refs. 41, 42) or PBS twice weekly for 4 weeks (Supplemental Figure 10A). The data revealed that rSema3A treatment decreased subendocardial fibrosis thickness and AF inducibility in TGF-β–transgenic mice (Supplemental Figure 10, B and C). Accordingly, we sought to determine whether Sema3A is effective at preventing EndMT in vivo. rSema3A administration significantly reduced EndMT marker levels, as determined by immunohistochemical analysis (Supplemental Figure 10, D–F). These results indicated that antagomir-181b and rSema3A can reduce the degree of atrial fibrosis and AF vulnerability by downregulating miR-181b.

### miR-181b overexpression and decreased Sema3A expression in atria are associated with AF.

**Figure 8:**
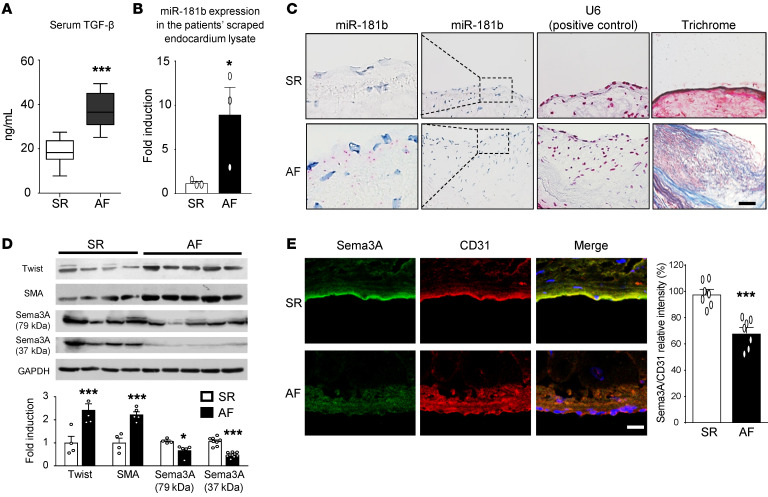
miR-181b and Sema3A expression in human AF. (**A**) TGF-β serum levels in patients with AF or SR. Data are presented as the mean ± SEM; (*n =* 15). (**B**) qRT-PCR analysis of miR-181b in AEECs derived from the atrium of patients with AF or SR with U6 used as the loading control. Data are presented as the mean ± SEM (*n =* 3). (**C**) RNA CISH analyses of miR-181b in atrial tissue sections from patients with AF. miR-181b signal (red dots) and U6, used as a positive control (red color), were observed in the atrial endothelial edge in tissue, and trichrome staining showed miR-181b expression in the atrial subendocardial fibrotic tissue (*n =* 3) of these patients. Scale bar: 50 μm. Original magnification x400; enlarged insets x800. (**D**) Western blot analysis of Twist, SMA, and Sema3A in the atrium of patients with AF (*n* = 5) or SR (*n* = 4). Relative protein expression values were normalized to GAPDH. (**E**) Immunohistochemical analysis of Sema3A and CD31 in the endocardium (scale bar: 50 μm), and quantitation of Sema3A expression versus CD31 in the endocardium (*n =* 7 per group). All data are presented as the mean ± SEM. (**A**, **B**, **D**, and **E**) *P < 0.05 and ***P < 0.001 versus the SR group, by 2-tailed Student’s *t* test.

We further evaluated miR-181b and Sema3A expression in atria of patients with AF. Atrial appendage specimens were acquired from patients with AF and patients with SR, and serum TGF-β concentrations were measured. We evaluated miR-181b expression in tissue scraped from atrial samples, and assessed Twist, SMA, and Sema3A expression levels in whole atrial tissue. We found that AF patients had increased serum TGF-β levels (Figure 8A) and miR-181b levels (Figure 8B). To localize miR-181b expression in the atrial endocardium, we performed a specific RNA CISH assay for miR-181b with atrial tissue sections from patients with AF or SR. This assay showed that miR-181b was expressed at the edge of the atrial endothelium in patients with AF and confirmed the location of miR-181b expression in atrial subendocardial fibrotic tissue (Figure 8C). Western blot analysis revealed the upregulation of Twist and SMA and the downregulation of Sema3A in patients with AF (Figure 8D). Consistent with the Western blot findings, Sema3A expression was significantly decreased in the atrial endocardium of patients with AF (Figure 8E) but that Twist and SMA levels were increased (Figure 1, B and C). Together, these results firmly established that miR-181b is involved in mediating TGF-β–induced EndMT and atrial subendocardial fibrosis through the disruption of Sema3A, which is physiologically significant in AF.

## Discussion

**Figure 9:**
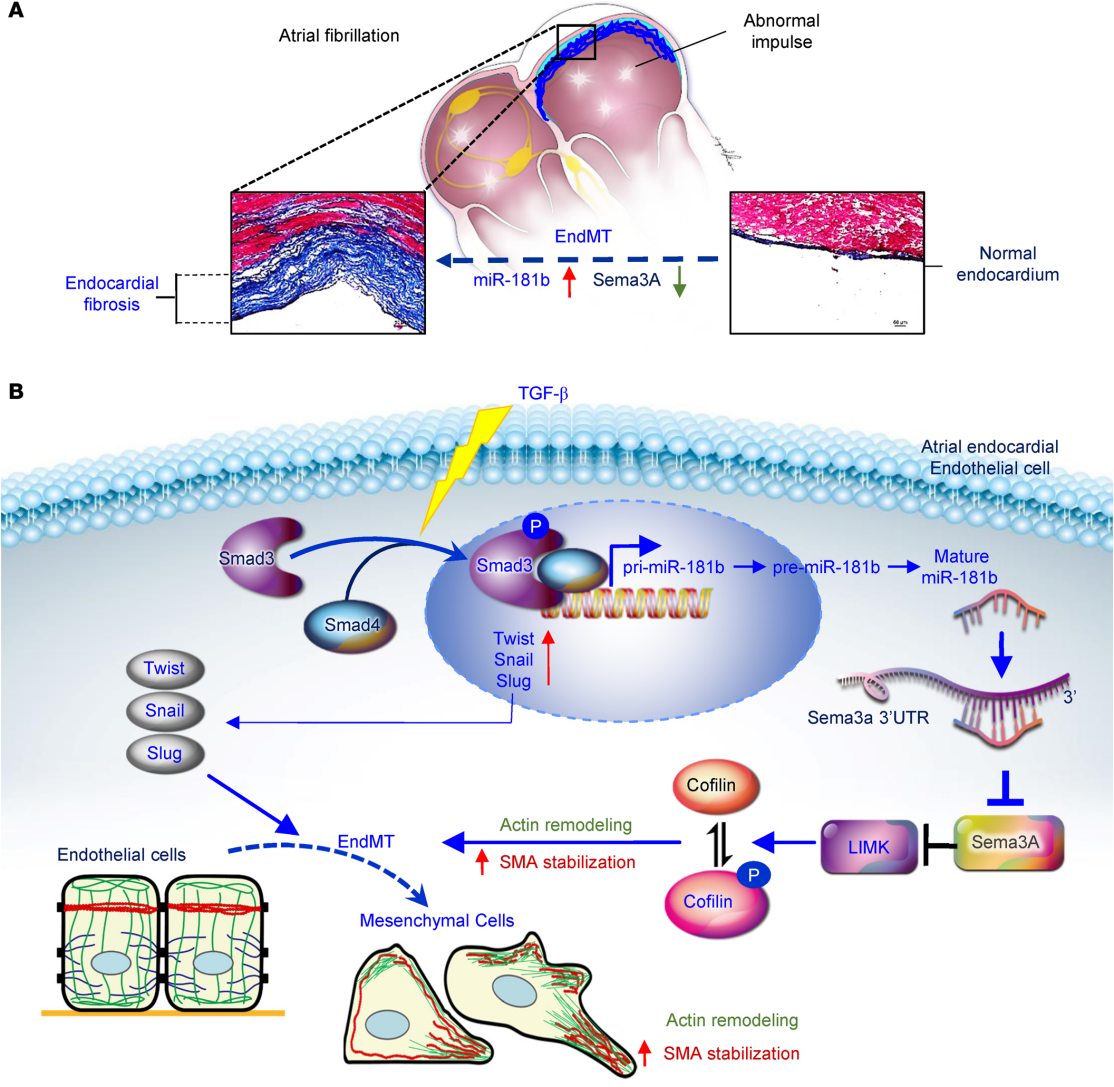
Schematic illustration of the mechanism linking miR-181b and Sema3A in a fundamental TGF-β-induced EndMT process and atrial subendocardial fibrosis. (**A**) A mechanism of fundamental TGF-β–induced EndMT and atrial subendocardial fibrosis was identified. (**B**) TGF-β activates p-SMAD3, which binds to SMAD4 and forms the SMAD complex. This complex translocates to the nucleus and promotes miR-181b transcription. Mature miR-181b targets the 3′-UTR of Sema3A mRNA for degradation. Insufficient expression of Sema3A protein, which is needed to increase LIMK/p-cofilin signaling, leads to actin remodeling, lamellipodium formation, increased SMA stabilization, and the EndMT process, thus participating in the atrial fibrosis.

This study illustrates a mechanism linking miR-181b and Sema3A in a fundamental TGF-β–induced EndMT process and atrial subendocardial fibrosis. Altogether, our findings substantiate the involvement of miR-181b in mediating TGF-β–induced EndMT and atrial subendocardial fibrosis through the disruption of Sema3A, which is physiologically significant in AF. Moreover, our work indicated that the administration of miR-181b antagomir and rSema3A reversed atrial subendocardial fibrosis and reduced AF vulnerability in TGF-β–overexpressing mice. We identified miR-181b and Sema3A as mediators of EndMT and atrial fibrosis. The schema in Figure 9 shows the correlation between elevated miR-181b and reduced Sema3A with AF severity.

AF is frequently related to atrial fibrosis (2), and TGF-β is among the key profibrotic mediators (1, 5, 43, 44). AF may increase TGF-β expression in atrial myocytes. Previously, we showed that rapid electrical activation of cultured atrial myocytes induced TGF-β expression, and NADPH oxidase 2/4–dependent oxidative stress was identified as the underlying mechanism (45). These results were consistent in humans: AF was associated with TGF-β expression in the atrial myocardium of patients with chronic AF.

TGF-β is among the key mediators of EndMT/EMT via numerous signaling pathways in various physiologic and pathologic conditions (18, 19). Recently, Kato et al. observed EndMT in the atria of patients with AF (22). They found prominent fibrosis in the subendocardial region of AF atria, and the expression of EndMT markers, including Snail and S100A4, was associated with the severity of fibrosis. Nevertheless, they did not identify a detailed molecular mechanism for the involvement of EndMT in AF. Several studies have emphasized the role of interstitial fibrosis in AF initiation and the associated remodeling (46). In addition to the proinflammatory state, the profibrotic pathway is a highly plausible cause of arrhythmia recurrence. Substantial clinical and experimental evidence supports a central role for TGF-β, a profibrotic biomarker, in AF-related atrial fibrosis (2). Both SMAD2/3 and c-Jun N-terminal kinase are potential downstream effectors of TGF-β (47). The miR-181 family has causative roles in EMT and metastasis in various cancers. Taylor et al. showed that TGF-β increased miR-181a expression to promote breast cancer metastasis (48). Furthermore, miR-181b was shown to mediate TGF-β–induced EMT in lung cancer stem cells (49).

We showed that AF susceptibility induced by burst-pacing in cardiac-specific TGF-β–transgenic mice was significantly reduced by the intravenous administration of antagomir-181b, which decreased both subendocardial and interstitial fibrosis in atria; these reductions may contribute to its antiarrhythmic effect. Zheng et al. reported that miR-181b activates hepatic stellate cells via the PTEN/Akt pathway and therefore promotes hepatic fibrosis (50). Cardiac fibroblasts undergo several stages of differentiation, and better elaboration of these different states in the presence of miR-181b is required to elucidate the specific molecular mechanism of EndMT (51). Our study extends prior knowledge of the involvement of miR-181b in TGF-β–induced EndMT to the pathogenesis of atrial fibrosis and AF. We found that TGF-β upregulated miR-181b expression in atrial ECs in a SMAD3-dependent manner and that miR-181b played an essential role in EndMT. Furthermore, in WT and cardiac-specific TGF-β–transgenic mice, atrial miR-181b levels were positively correlated with serum TGF-β concentrations. Importantly, miR-181b levels in the atrial endocardium were higher in patients with AF than in those with SR. These observations suggest that miR-181b is a potential biomarker in AF. However, we did not clarify whether miR-181b levels are associated with AF itself or with the underlying atrial fibrosis. We speculate that one potential mechanism involves miRNA-containing exosomes (52). Notably, we found that AF susceptibility induced by burst-pacing in cardiac-specific TGF-β–transgenic mice was reduced by intravenous administration of antagomir-181b. This antagomir reduced both subendocardial and interstitial fibrosis in atria, and these reductions may contribute to its antiarrhythmic effect. Our study provides further documentation of the critical role of miR-181b in the development of atrial fibrosis and AF.

In this study, we showed that TGF-β promotes EndMT, contributing to fibrosis in AF atria, and determined that the Sema3A/LIMK/p-cofilin/actin axis was involved in EndMT/EMT. Actin remodeling is essential during EndMT (37–39), which is achieved when cell-cell adhesion is dissolved, the actin cytoskeleton remodels and projects lamellipodia, and cells acquire migratory capabilities (38). Dynamic actin remodeling and reorganization of the cytoskeleton facilitate invasive protrusions of the cell membrane, such as lamellipodia, and increase transitory cell migratory potential (37). The semaphorin family contains 21 genes, and the encoded proteins were initially described as axon guidance factors that regulate central nervous system development (32) and play roles in epithelial junctions, especially in cancer cells (53). Our study discovered that in AEECs, TGF-β and miR-181b downregulated Sema3A, leading to actin remodeling via the LIMK/p-cofilin pathway. Previous studies have reported that the Sema3A/LIMK/p-cofilin/actin axis is probably involved in EndMT/EMT. Overexpression of Sema3A inhibited cancer metastatic dissemination induced by antiangiogenic treatment in mice via the suppression of EMT (54). During EMT, tumor cells lose the epithelial marker E-cadherin and gain mesenchymal markers, such as Snail1 and vimentin, and these effects are abolished by the overexpression of Sema3A. Actin remodeling induced by Sema3A has been implicated as the underlying mechanism (32, 33, 55). Cofilin is a downstream target of LIMK, which acts to sever and depolymerize actin. The activation of p-cofilin and LIMK by TGF-β has been shown to mediate EMT by reorganizing the actin cytoskeleton and cell-cell adhesion in cancer cells (36). Binding of Rac1 by semaphorin receptor plexin A or B family members could inactivate p21-activated kinase, leading to dynamic actin remodeling through LIMK and cofilin (56). Moreover, Sema3A was shown to regulate the phosphorylation of cofilin by LIMK in nerve development (57). Our results showed that TGF-β induced cell membrane protrusions containing SMA, providing evidence of actin remodeling and reorganization. rSema3A decreased EndMT markers and rescued TGF-β–induced lamellipodia formation and actin remodeling. These results suggested that Sema3A depletion may contribute to TGF-β–induced EndMT via dynamic actin remodeling. Other semaphorins have been shown to participate in EMT, but with diverse effects. Sema3E, Sema4D, and Sema7A were shown to contribute to EMT (53, 58, 59), whereas Sema3B, Sema3F, and Sema5A inhibited EMT in cancer cells (53, 60, 61). Various studies have reported that Sema3C either facilitates or inhibits EMT (53, 62)

Twist is a marker of EndMT and EMT and promotes these processes. The ectopic expression of Twist results in the loss of E-cadherin–mediated cell-cell adhesion, activation of mesenchymal markers, dynamic remodeling of the cytoskeleton and actin, and increased cell migration ability (17). Consistent with the findings of previous studies, our results indicate that, in addition to the direct activation of EndMT/EMT-related proteins, the depletion of Sema3A by TGF-β and miR-181b negatively regulates LIMK/p-cofilin signaling and facilitates actin remodeling and reorganization, which are among the essential molecular processes of EndMT in AF. In addition, experiments in which Sema3A levels were increased with a peptide or decreased with siRNA in AEECs confirmed that Sema3A is upstream of Twist. In 2019, Li and colleagues identified the Neuropilin1 (*Nrp1*) gene as a direct transcriptional target gene of Twist through RNA-Seq and ChIP-Seq (63). NRP1 acts as a cell-surface receptor for Sema3A and affects cell survival and migration, which are essential for tumor progression and EMT (64). As a ligand, Sema3A binds to NRP1 and inhibits Twist transcription (63). However, there remain considerable unknowns regarding how Sema3A downregulation permits Twist upregulation, and future work is needed to confirm the mechanism.

Another possible explanation for subendocardial fibrosis is that subendocardial fibroblasts are activated by factors secreted from local endocardial ECs or myocytes in TGF-β–transgenic mice. In the TGF-β–transgenic mice in this study, TGF-β was produced and secreted from atrial myocytes rather than endocardial ECs. Our study did not exclude this as a likely cause of subendocardial fibrosis in AF. However, our study showed coexpression of CD31 and EndMT markers in regions of the subendocardium in TGF-β–transgenic mice, thus reducing the likelihood that it contributes to subendocardial fibrosis.

### Limitations and unanswered questions.

First, there were regional differences in the degree of EndMT and thickness of the subendocardial fibrosis in atria from patients with AF, tachypacing-induced HF rabbits, and TGF-β–transgenic mice. We did not evaluate or explain the regional differences in the atrium, and we obtained only left atrial appendage samples. Second, although we showed that neutralization of miR-181b attenuated atrial EndMT and atrial fibrosis, which was associated with reduced AF vulnerability, the degree to which EndMT contributes to subendocardial fibrosis and general atrial fibrosis is unclear. Third, since the proliferation of ECs may participate in the pathogenesis of subendocardial fibrosis, whether miR-181b/Sema3A signaling is also involved in EC proliferation is worthy of further study. Finally, our results suggested that AEECs were likely an important source of atrial fibroblasts that contributed to fibrosis via TGF-β–mediated EndMT. These results were limited, in that we did not further investigate the dynamic changes in EC markers during the different stages of EndMT or after this process was completed, nor did we examine the activation and proliferation of the mesenchymal cells and fibroblasts. Our study identifies a mechanistic link from the induction of EndMT by TGF-β via miR-181b/Sema3A signaling in AF. Much remains unknown regarding how AF-related endocardial mesenchymal transition differs from EndMT or endocardial fibroelastosis (EFE) of the ventricular endocardium, and future work is needed to confirm the mechanism.

### Conclusions.

AF and various underlying systemic diseases promote atrial fibrosis, which makes it difficult to achieve rhythm control in patients with long-duration AF. The sources of activated atrial fibroblasts that accumulate in AF remain largely unclear. This study illustrates a mechanism involving miR-181b and Sema3A in a fundamental TGF-β–induced EndMT process and atrial subendocardial fibrosis. Our work showed that the miR-181b antagomir reversed atrial subendocardial fibrosis and reduced AF vulnerability in TGF-β–transgenic mice. This study identified what we believe to be a previously unrecognized pathway, TGF-β/miR-181b/Sema3A/LIMK/p-cofilin, and characterized miR-181b and Sema3A as potential therapeutic targets for the treatment and prevention of AF.

## Methods

An expanded material and methods section is available in the Supplemental Methods. See complete unedited blots in the supplemental material. The sequences of miRNA, siRNA, and qRT-PCR are listed in Table 2.

### Statistics.

Statistical analyses were performed using GraphPad Prism 7.0.3 (GraphPad Software). A 2-tailed Student’s *t* test and 1-way ANOVA with post hoc (Bonferroni’s or Tukey’s) test were applied for comparisons of 2 groups and multiple groups, respectively. Fisher’s exact test was used to compare categorical variables between groups. A *P* value and confidence level of less than 0.05 (95% CI) were considered to indicate statistical significance. Unless otherwise noted, data represent the mean ± SEM. The original data files of the microarray and next-generation sequencing (NGS) data have been deposited in the NCBI’s Gene Expression Omnibus (GEO) database (GEO GSE156835 and GSE201318; http://www.ncbi.nlm.gov/geo).

### Study approval.

Human atrial appendage samples were obtained from patients undergoing cardiac surgery. Informed consent was obtained from all participating patients. The study protocol was approved by the IRB of the Chang Gung Medical Foundation (IRB no. 104-2436A3), and the study was conducted in accordance with the principles of the Declaration of Helsinki. All animal study procedures and experiments were reviewed and approved by the IACUC of Chang Gung Memorial Hospital (IACUC approval nos. 2007100501 and 2016120805) and were in accordance with NIH guidelines for the care and use of animals. Housing and maintenance were provided by Chang Gung Memorial Hospital, and all animals were fed a standard chow diet with free access to water.

## Author contributions

YHY, WJC, and YJL had full access to all of the data in this study and take responsibility for the integrity of the data and the accuracy of the data analysis. YJL, FCT, WJC, and YHY conceptualized and designed the study. YHY, SHC, GJC, CCH, and YJL performed experiments and analyzed and interpreted the data. YJL and YHY wrote the manuscript.

## Supplementary Material

Supplemental data

## Figures and Tables

**Table 2:**
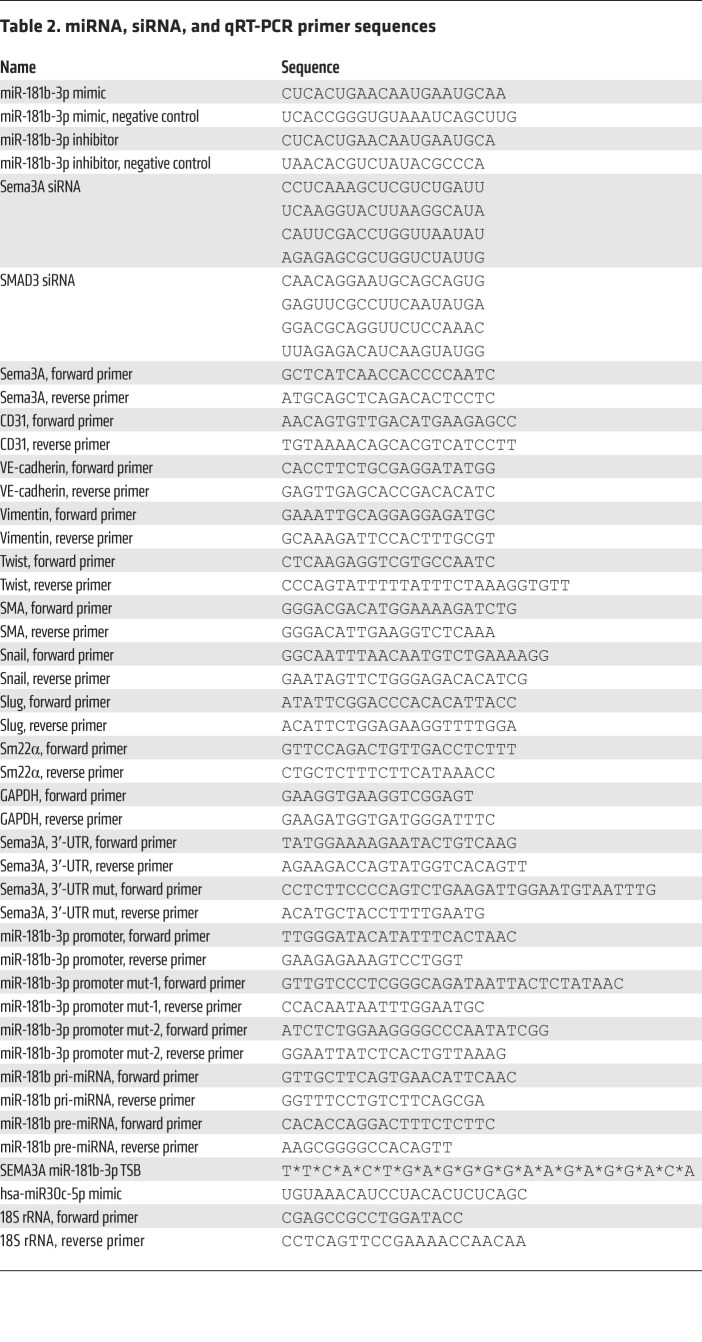
miRNA, siRNA, and qRT-PCR primer sequences

**Table 1:**
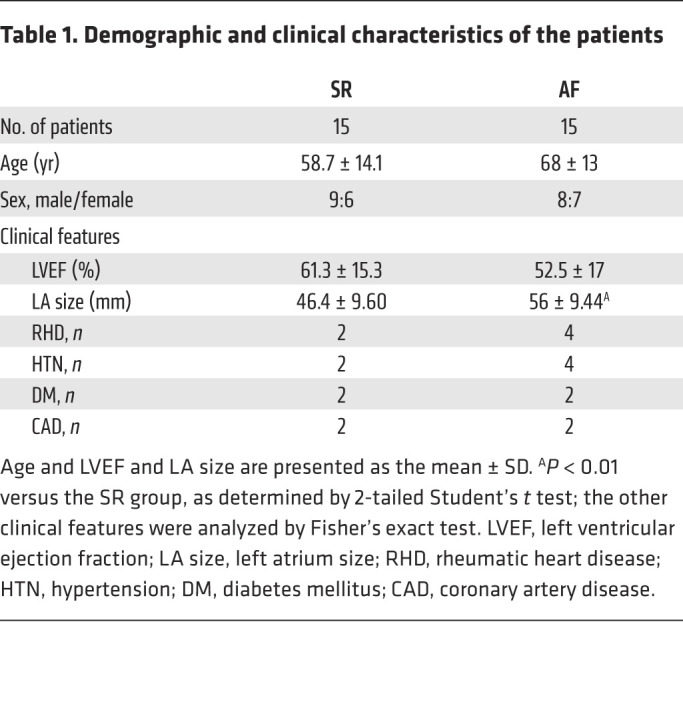
Demographic and clinical characteristics of the patients

## Version 1 07/01/2022

Electronic publication
