# Supplementary material for: miR-181b targets semaphorin 3A to mediate TGF-β–induced endothelial-mesenchymal transition related to atrial fibrillation
Source: J Clin Invest. 2022 Jul 1;132(13):e142548. doi: 10.1172/JCI142548 (PMC9246393; doi:10.1172/JCI142548)

Supplemental Material

MiR-181b targets semaphorin 3a to mediate TGF-β-induced endothelial-to-mesenchymal transition related to atrial fibrillation

Ying-Ju Lai[1,2,3#], Feng-Chun Tsai[4, 5#], Gwo-Jyh Chang[1,6], Shang-Hung Chang[1,5], Chung-Chi Huang[2,7], Wei-Jan Chen[1,5]*, Yung-Hsin Yeh[1,5]*

[1]Cardiovascular Department, Chang Gung Memorial Hospital, Tao Yuan, [2]Department of Respiratory Therapy, Chang Gung University College of Medicine, Tao Yuan. [3]Department of Respiratory Care, Chang-Gung University of Science and Technology, Chia Yi. [4]Department of Thoracic and Cardiovascular Surgery, Chang Gung Memorial Hospital. [5]Department of medicine, Chang Gung University College of Medicine, [6]Graduate Institute of Clinical Medical Sciences, Chang Gung University College of Medicine, [7] Department of Pulmonary and Critical Care Medicine, Chang Gung Memorial Hospital, Tao Yuan, Taiwan

Address for Correspondence:
Yung-Hsin Yeh, MD
The Cardiovascular Department, Chang Gung Memorial Hospital, Fu Shin Rd. No. 5, Kwei Shan, Tao Yuan 333, Taiwan
Tel: 886-3-3281200 ext 8162
Fax: 886-3-3271192
E-mail: yeongshinn@cgmh.org.tw

Wei-Jan Chen, MD, PhD
The Cardiovascular Department, Chang Gung Memorial Hospital, Fu Shin Rd. No. 5, Kwei Shan, Tao Yuan 333, Taiwan
Tel: 886-3-3281200 ext 8162
Fax: 886-3-3271192
E-mail: wjchen@cgmh.org.tw

# Drs Lai and Tsai contributed equally to this work.
* Drs Yeh and Chen contributed equally to this article

Human atrial appendage specimens

Atrial appendage samples were obtained from 15 patients with AF and 15 patients with sinus rhythm (SR) undergoing cardiac surgery (Table 1). Informed consent was obtained from all participating patients. The study protocol was approved by the Chang Gung Medical Foundation Institutional Review Board, (IRB 104-2436A3) and the study was conducted in accordance with the principles of the Declaration of Helsinki.

Histological analysis of atrial subendocardial fibrosis (ASF) thickness

For all histological procedures, atrial tissues were fixed with 10% neutral formalin, immersed in formalin overnight, and embedded in paraffin. Sections (5 μm thick) were processed for modified Masson's trichrome staining according to the manufacturer's instructions (ScyTek, catalog TRM-2-IFU, Logan, UT, USA). Trichrome staining of subendocardial fibrosis samples shows collagen (blue) deposition in the endocardium. Quantitative analysis of the thickness of collagen deposits by a color deconvolution method revealed a significant reduction in the percentage of the fibrotic area. All atrial sections were divided randomly into 6 fields, and trichrome-stained ASF were measured. By microscopic examination under 200X (human and rabbit tissue sections) or 400X (mouse tissue sections) magnification, ASF was defined as the distance between the elastic laminae to the fibrotic lumen using Nikon NIS-elements 4.3.00 BR software (Supplemental Figure 1).

Rabbit HF model

All animal experiments and study protocols were reviewed and approved by the Institutional Animal Care and Use Committee of Chang -Gung Memorial Hospital (CGMH-2007100501). All surgeries were performed under anesthesia, and all efforts were made to minimize suffering. Male New Zealand rabbits weighing 2-3 kg underwent the HF surgery. The rabbits were anesthetized by intramuscular injections of Zoletil (15 mg/kg) (Virbac, Carros, France) and Rompun (5 mg/kg) (Bayer, leverkusen, Germany). Then, the rabbits were placed on the clean surgery table, and anesthesia was maintained with 3% isoflurane (AbbVie, Lake County, IL, USA) by endotracheal intubation. While ensuring ventilation compliance, the right chest was opened at the third intercostal space, and an epicardial pacing lead was placed in the lateral wall of the right ventricle and connected to an Itrel III pacemaker (Medtronic, Inc., Minneapolis, MN, USA) for tachycardia pacing. After 1 week of convalescence, the rabbit ventricle was paced at 372 beats/min for 3 weeks.

Generation of transgenic mice with cardiac TGF-β expression

MHC-TGF-Cys33Ser transgenic mice (the generous gift from Loren J. Field, James Whitcomb Riley Hospital for Children, IN) were generated using the mouse α-cardiac MHC promoter and the human TGF-β cDNA sequence as described previously (1, 2). The resulting pups were screened by diagnostic polymerase chain reaction (PCR). For all surgeries, mice were anesthetized with a combination of Zoletil (20 mg/kg) (Virbac, Carros, France) and

Rompun (5 mg/kg) (Bayer, leverkusen, Germany) by intraperitoneal injection. After confirming a fully anesthetized state (e.g., no response to toe pinching), mice received programmed electrical stimulation, and the right/left atria and ventricles were dissected after sacrifice. All animal experiments were reviewed and approved by the Institutional Animal Care and Use Committee of Chang Gung Memorial Hospital (IACUC: 2016120805).

Isolation and characterization of human atrial endocardial endothelial cells (AEECs)

We developed a method for obtaining primary atrial EC cultures from human atrial appendages. To establish the AEECs isolation and culture protocol, we modified the pulmonary arterial EC isolation and culture protocol from Rabinovitch's laboratory at Stanford University (3). Atria were isolated from patients and rinsed 3 times in Hank's balanced salt solution (HBSS) (Gibco, catalog 14025-092, Grand Island, NY, USA) at 4°C to remove possible blood clots. The left atrium was then placed in a 50 ml conical tube and incubated with 0.4 mg/ml collagenase (Sigma-Aldrich, catalog C9722, Burlington, MA, USA) in HBSS at 37°C on a rotating platform for 20 min. After removing the collagenase solution, the inside of the left atrium was scraped gently with a 10-mm round glass slide to obtain the loosely attached EC layer and M199 (Gibco, 11150-059, Rockville, MD, USA) containing 10% FBS (Gibco, catalog 26140-079) was added to harvest the cells. Then, the cell suspension was filtered using a 100 μm cell strainer (Corning Falcon, catalog 352360, Durham, NC, USA) and centrifuged for 1 min. The pellet was resuspended in M199

containing 10% FBS, and the cells were seeded on gelatin (Acros Organics, catalog AC611990000, Fair Lawn, NJ, USA)-coated culture dishes for 20 min at 37°C. Then, the medium was removed, and fresh EC growth medium containing 20% FBS (Supplemental Figure. 2A) was added. The culture medium was renewed every 3 days until the cells reached 80% confluence. The ECs were purified by incubation for 20 min at 37°C with magnetic beads (30 μl, Dynabeads; Invitrogen, catalog 11155D, Waltham, MA, USA) conjugated with an anti-CD31 antibody. The cells bound to the beads were recovered with a magnet and washed five times with PBS containing 0.1% BSA (Sigma-Aldrich, catalog A7906, Burlington, MA, USA). The AEECs were then seeded on gelatin-coated culture dishes with EC growth medium. We utilized the endothelial-specific markers CD31 (Abcam, catalog ab28364, Cambridge, UK), and eNOS (BD Bioscience, catalog 610299, Franklin Lakes, NJ, USA) to characterize AEECs (Supplemental Figure 2B). All endothelial-specific markers were downregulated at passage 4 (Supplemental Figure 2C) and thus, the AEECs were utilized before passage 4 for the in vitro experiments. Human umbilical vein endothelial cells (HUVECs) were obtained and cultured in 0.1% gelatin-coated 100-mm dishes with EC growth medium (Lonza, catalog CC3156 and CC4176, Walkersville, MD, USA) and used between passages 5 and 7.

Immunocytochemical analysis

Immunocytochemical analysis of AEECs was performed with primary antibodies against

eNOS (BD Bioscience, catalog 610299, Franklin Lakes, NJ, USA), SMA (Abcam, catalog ab5694, Cambridge, UK), Twist (NOVUS, catalog, NBP2-37364, Briarwood Avenue Centennial, CO, USA), CD31 (Abcam, catalog ab28364, Cambridge, UK), and p-Cofilin (Cell Signaling Technology, catalog 3313, clone 77G2, Danvers, MA, USA), At the end of the experiments, cells were fixed with cold methanol; blocked with 1% goat serum/1% BSA in PBS for 30 min, and then incubated with primary antibodies for 1 h. Subsequently, the AEECs were incubated with Alexa-488-conjugated (green) (Abcam, catalog ab150113) and Cy3-conjugated (red) (abcam, catalog ab97075) secondary antibodies and then subjected to rhodamine-phalloidin (Abcam, catalog ab235138, Cambridge, UK) staining (red) for filamentous actin to observe lamellipodium formation. Nuclei were visualized by 4,6-diamidino-2-phenylindole (DAPI) staining (Invitrogen, catalog P36935, Waltham, MA, USA). Fluorescence was observed with a confocal microscope (Leica, Confocal TCS SP8XL, Wetzlar and Mannheim, Germany) at the Microscope Core Laboratory of Chang Gung Memorial Hospital.

Microarray data generation and analysis

Total RNA was isolated from AEECs using TRIzol reagent (Invitrogen, catalog 15596018) and further concentrated using the PureLink^{TM} RNA Mini kit (Invitrogen, catalog 12183018A). Total RNA quality and quantity were evaluated by using a Bioanalyzer 2100 (Agilent Technologies, Santa Clara, CA). Gene expression profiles were analyzed by using

the Affymetrix Human Transcriptome Array 2.0**.** (Affymetrix, Santa Clara, CA) (Supplemental Figure 3B), and array data were analyzed by the Genomic Medicine Research Core Laboratory of Chang Gung Memorial Hospital, Lin-Kou.

Next-generation sequencing and data pre-processing

Tissues were obtained from atria of mice and preserved in RNA*later* Stabilization Solution (Invitrogen, catalog AM7020) directly after resection and stored at 4 °C. About 50~60 mg of tissues was used for RNA extraction using TRIzol Reagent (Invitrogen, catalog 15596018) according to the manufacturer's instructions. The quality of RNA was evaluated by LabChip GX, and tissue extraction RNA integrity number (RIN) greater than 7 were further processed. For the small RNA-seq, we prepared libraries using NEBNext Multiplex small RNA library Pre Set for Illumina kits according to manufacturer's instructions. Briefly, 1 μg of purified total RNA was ligated with adapter and PCR amplified. The small RNA libraries were size selected with a target insert size of 15~30 nt in length using the 6% TBE PAGE gel. The yield and size distribution of the purified RNA and small RNA libraries were assessed using the Agilent 2100 Bioanalyzer instrument with the Agilent High Sensitivity DNA Kit (Agilent Technologies, catalog 5067-4626). Equal amounts of libraries were pooled in molecular ratio and consequently sequenced by the NextSeq 500 sequencer. Small RNA-Seq raw read counts were normalized to reads per million (RPM) for quantitative representation. Principal component analysis (PCA) analysis was performed on both sequencing data sets using R

package 'ggbi plot' and 'stats'. For the differential expression analysis. R/BioConductor 'DESeq. 2' was used to perform the differential expression analysis. We defined differentially expressed genes (DEGs) and miRNAs (DEMs) in the TGF-β transgenic mice/ WT mice comparison on the basis of P value <0.05 and -2 >, fold change > 2. For the comparison among groups, differential expression analysis was performed for the samples and DEGs were defined by p-value <0.05 and fold change > 2.

Quantitative reverse transcription-PCR (qRT-PCR)

miRNAs were isolated from AEECs with the miRNeasy mini kit (Qiagen, catalog 217004, Hilden, Germany) according to the manufacturer's protocol. Real-time qRT-PCR was performed as described previously (4). Briefly, total RNA containing miRNA was polyadenylated, and cDNA was synthesized using a poly(T) primer with a 3' degenerate anchor and a 5' universal tag. Then, cDNA served as the template for miRNA qRT-PCR using the miCURY LNA^{TM} RT kit (Qiagen, catalog 339340) and the following Qiagen-validated primers: miR-181b-specific primers (hsa-miR-181b-3p target sequence: 5'-CUCACUGAACAAUGAAUGCAA-3') and U6 snRNA control primers (Qiagen, catalog YP00203907, Hilden, Germany). qRT-PCR analysis of primary (Pri), precursor (Pre) transcripts of miR-181b in AEECs using 18S rRNA as the loading control. qRT-PCR assays were performed using the 7900HT Fast Real-Time PCR System (Applied Biosystems, Foster city, CA) with the following program: denaturation at 95°C for 10 min, followed by 40 cycles

of 95°C for 10 s and 60°C for 60 s, and ending with melting curve analyses. To determine Sema3a RNA expression levels, total cellular RNA was extracted using a PureLink™ RNA Mini kit (Invitrogen, catalog 12183018A) according to the manufacturer's protocol, and qRT-PCR was performed as described previously. GAPDH mRNA was used as an internal control. The primers were as follows: Sema3a forward: 5'-GCTCATCAACCACCCCAATC-3' and reverse: 5'-ATGCAGCTCAGACACTCCTC3'; and GAPDH forward: 5'-GAAGGTGAAGGTCGGAGT-3' and reverse: 5'-GAAGATGGTGATGGGATTTC-3'. Relative Sema3a expression was calculated using the $2^{-\Delta\Delta Ct}$ method with SYBR Green detection.

Western blot analysis

Proteins were extracted and processed as described previously (4). Western blotting was performed using primary antibodies against CD31 (Abcam, catalog ab28364), eNOS (BD Bioscience, 610299), SMA (Thermo Scientific, catalog MS-113-P, clone 1A4, Waltham, MA, USA), HGF (Abcam, catalog ab83760), Sema3A (Abnova, catalog H00010371-M01, clone 5G9, Taipei, Taiwan), VE-Cadherin (Cell Signaling Technology, catalog 2500, clone D87F2), p-Vimentin (Ser83) (Cell Signaling Technology, catalog 3878), p-Vimentin (Ser56) (Cell Signaling Technology, catalog 3877), total Vimentin (Cell Signaling Technology, catalog 9775, clone D21H3), Twist (NOVUS, catalog, NBP2-37364), Snail (Cell Signaling Technology, catalog 3879, clone C15D3), Slug (Cell Signaling Technology, catalog 9585,

clone C19G7), SM22α (Abcam, catalog ab14106), LIM kinase (LIMK) (Cell Signaling Technology, catalog 3845, clone 8C11), p-Cofilin (Cell Signaling Technology, catalog 3313, clone 77G2), total Cofilin (Cell Signaling Technology, catalog 5175, clone D3F9) and Collagen (Abcam, catalog ab21286). Signals were detected using the ECL detection method (Millipore, catalog WBULS0500, Temecula, CA, USA) and quantified by densitometry. Immunoreactive bands were in the linear range, and the results are presented relative to the bands corresponding to the GAPDH (Santa Cruz Biotechology, catalog SC-32233, clone 6C5, Dallas, TX) or tubulin antibody (Santa Cruz Biotechnology, catalog 32293, clone DM1A).

Small interfering (si) RNA, miR-181b antagomir, target site blockers and miR-181b mimic in AEECs

Chemically synthetic siRNA targeting human Smad3 (Dharmacon, catalog L-020067-00-0005 Lafayette, CO) or, Sema3a (Dharmacon, catalog L-020091-00-0005), and control siRNA were purchased from Dharmacon (Thermo Fisher Scientific Inc). The siRNAs sequences are presented in Table 2. AEECs at 70% confluence were transfected with 25 nM siRNA for 24 h using Lipofectamine^{TM} RNAi MAX Transfection Reagent (Invitrogen, catalog 13778-150, Waltham, MA, USA) according to the manufacturer's instructions. Next, the transfected cells were cultured in regular culture medium, and protein was collected 48 h after transfection. The knockdown efficiency was evaluated at 48 h by measuring protein levels in lysates via Western immunoblotting (see Western blot analysis). The miR-181b-3p

mimic (Qiagen, catalog YM004700056) and antagomir (antagomir-181b) (Qiagen, catalog YI04104675) and negative control oligonucleotides (Qiagen, catalog YM00479902 and YI00199006) were purchased from Qiagen. Oligomers (100nM) were transfected into AEECs using DharmaFECT 1 (Dharmacon, catalog T-2001-01, Lafayette, CO, USA) according to the manufacturer's instructions. To interfere with the binding of miR-181b to its targets, miRCURY LNA™ miRNA Power Target Site Blockers (Qiagen catalog YT0071336) with the following custom sequences were used according to the manufacturer's instructions.: 5'-TTCACTGAGGGGAAGAGGACA-3'.

Luciferase reporter assay for the Sema3a 3' untranslated region (UTR).

For the luciferase reporter experiments, the Sema3a 3'UTR was amplified by PCR from human genomic DNA (forward: 5'-TATGGAAAAGAATACTGTCAAG-3'; reverse: 5'-AGAAGACCAGTATGGTCACAGTT-3'). The PCR product was ligated into the pMIR luciferase reporter (pMIR-REPORT System, Ambion). AEECs were transfected with *Trans*IT®-LT1 transfection reagent (Mirus, catalog MIR2300, Madison, WI) according to the manufacturer's protocol. Site-directed mutagenesis of the putative miR-181b binding sites was performed by PCR using the Q5 Site-Directed Mutagenesis kit (New England Biolabs, catalog E0554, Ipswich, MA). All sequences related to PCR are presented in Table 2. Luciferase activity was measured with a luciferase assay kit (BioThema, catalog 484-001, Handen, Sweden) and detected by a luminometer (Promega, GloMax® Luminometer,

Madison, WI).

Luciferase reporter assay for miR-181b promoter activity

For the luciferase reporter experiments, a fragment of the human miR-181b promoter (nucleotides -1200 to +1) was amplified by PCR from human genomic DNA (forward: 5'-TTGGGATACATATTTCACTAAC-3'; reverse: 5'-GAAGAGAAAGTCCTGGT-3'). The sequences used to detect miR-181b promoter activity are presented in Table 2. The PCR product was ligated into the pGL3-Basic vector (Promega, Madison, WI) at the KpnI and XhoI sites. Putative Smad3 binding elements (SBEs, -1091 to -1085 and -404 to -399) were found in the miR-181b promoter. Site-directed mutagenesis of the SBEs was performed by PCR using the Q5 Site-Directed Mutagenesis kit (New England Biolabs, catalog E0554). AEECs were transfected with *Trans*IT®-LT1 transfection reagent (Mirus, catalog MIR2300) according to the manufacturer's protocol. Luciferase activity was measured with the luciferase assay kit (BioThema, catalog 484-001) and detected by a luminometer (Promega, GloMax® Luminometer).

Immunohistochemistry

Immunohistochemical analysis was performed with primary antibodies against TGF-β (Abcam, catalog ab64715, clone 9016), CD31 (Abcam, catalog ab28364) (abcam, catalog ab24590, clone P2B1), SMA (Abcam, catalog ab7817, clone 1A4) (Abcam, catalog ab5694), Twist (Abcam, catalog ab175430, clone 10E4E6) (Abcam, catalog ab50581), Snail (Cell Signaling Technology, catalog 3879, clone C15D3) , Slug (Cell Signaling Technology, catalog 9585, clone C19G7), Vimentin (Santa Cruz Biotechology, catalog SC-6260, clone V9)

and Sema3A (Abnova, catalog H00010371-M01, clone 5G9) and with Alexa-488-conjugated (green) (Abcam, catalog ab150113) and Cy3-conjugated (red) (abcam, catalog ab97075) secondary antibodies. Nuclei were visualized by DAPI staining (Invitrogen, catalog P36935). Fluorescence was observed with a confocal microscope (Leica, Confocal TCS SP8XL) at the Microscope Core Laboratory of Chang-Gung Memorial Hospital. The fluorescence intensity was detected and calculated using "NIS Elements 4.30.00" imaging software from Nikon.

miR-181b chromogenic in situ hybridization.

The cellular localization of miR-181b was investigated by chromogenic in situ hybridization (CISH) using a modified version of the RNA Scope 2.5 high-definition procedure (Advanced Cell Diagnostics [ACD], Hayward, CA, USA)(5, 6). In brief, 5-μm thick paraffin sections from tissue samples were pretreated and hybridized with miRNAscope™ Probe-SR-hsa-miR-181b-3p-S1, targeting 2-20 of MIMAT0022692, (Advanced Cell Diagnostics, catalog 1075841-S1) targeting miR-181b overnight. The ZZ-pairs binding miR-181b were detected using seven amplification steps, including a Tyramid Signal Amplification step (TSA-DIG; PerkinElmer, catalog NEL748001KT, Skovlunde, Denmark) labeled with alkaline phosphatase-conjugated sheep anti-DIG FAB fragments (Roche, catalog 11093274910, Basel, Switzerland), before visualized with Liquid Permanent Red (DAKO, catalog K0640, Glostrup, Denmark) and counterstained with Mayer's hematoxylin. Data were collected using a using "NIS Elements 4.3.00" Imaging software from Nikon.

Systemic delivery of miR-181b antagomir or recombinant Seam3A

Systemic antagomir delivery was achieved as described previously (7). Eight-week-old mice were injected through the tail vein with miR-181b-3p antagomir or miRNA nonspecific control twice a week for 4 weeks. In brief, 3 μl of 1 nmol/μl miRNA nonspecific control or miR-181b-3p antagomir (Life Technologies, catalog MH20327) was mixed with 22 μl Opti-MEM™ I Reduced-Serum Medium (Gibco, catalog 31985-070). Lipofectamine™ RNAiMAX Transfection Reagent (20 μl) (Invitrogen, catalog 13778-150) was mixed with

DPBS (5 μl) (Gibco, catalog 14190-136) (solution 2) and placed at room temperature for 15 min. Solutions 1 and 2 were then mixed by gentle pipetting. After incubation at room temperature for 30 min, the mixture added 50 μl normal saline (100 μl per mouse) was injected via the tail vein **(Figure 7A)**. To investigate the potential therapeutic of Sema3A, the prepared recombinant sema3A (rSema3A) (Sino Biological, catalog 10758-H01H, Beijing, China). As previously sema3A intervention *In Vivo* studies(8, 9), eight-week-old mice were injected through the tail vein with rSema3A (1mg/kg body weight) or PBS (as control) twice a week for four weeks.

Programmed electrical stimulation

Twelve-week-old mice received programmed transesophageal electrical stimulation as previously described (4). Briefly, a 4 French electrode catheter (St. Jude Medical, MN, USA) connected to an isolated stimulator (IX-TA-220, iWork Systems Inc, WA, USA) was inserted through the esophagus of anaesthetized mice for atrial pacing. The pacing programs and EKG recordings were managed by LabScribe software (iWork Systems Inc, WA, USA). A stimulus amplitude of 1.5X diastolic capture threshold was used with a burst stimulus duration of 2 ms. A pretest burst was performed with a cycle length of 100 ms for 10 s to ensure the position and capture of atrial stimulation. Then, a pacing burst with a cycle length of 40 ms for 3 s was administered and repeated 10 times. AF was defined as rapid irregular atrial rhythm lasting longer than 3 s. AF inducibility was expressed as the ratio of pacing-triggered AF episodes/10 pacing bursts in each mouse. The AF duration was presented as the AF episode in each mouse **(Supplemental Figure 9).**

Enzyme-linked immunosorbent assay (ELISA)

Serum TGF-β (Human, R&D System, DY240-05, Minneapolis, MN, USA) (Mouse, R&D System, DY1679-05, Minneapolis, MN, USA) protein level was evaluated using ELISAs, which were processed according to the manufacturer's instructions.

Statistical analysis

Statistical analysis was performed using GraphPad Prism 7.03 statistical software. Unless otherwise noted, data represent mean ± SEM. 2-tailed Student's t test and one-way analysis of variance (ANOVA) with the post hoc (Dunnett's and Bonferroni's) test were applied for comparisons of 2 groups and multiple groups, respectively. The Fisher's exact test was used to compare categorical variables between groups. Linear regression test was used to compare correlation between groups. A P value less than 0.05 was considered significant. The original data files of the microarray and next-generation sequencing (NGS) have been deposited in Gene Expression Omnibus (http://www.ncbi.nlm.gov/geo) with the access number GSE156835 and GSE201318.

1. Nakajima H, Nakajima HO, Salcher O, Dittie AS, Dembowsky K, Jing S, et al. Atrial but not ventricular fibrosis in mice expressing a mutant transforming growth factor-beta(1) transgene in the heart. *Circ Res.* 2000;86(5):571-9.
2. Verheule S, Sato T, Everett Tt, Engle SK, Otten D, Rubart-von der Lohe M, et al. Increased vulnerability to atrial fibrillation in transgenic mice with selective atrial fibrosis caused by overexpression of TGF-beta1. *Circ Res.* 2004;94(11):1458-65.
3. Sawada H, Saito T, Nickel NP, Alastalo TP, Glotzbach JP, Chan R, et al. Reduced BMPR2 expression induces GM-CSF translation and macrophage recruitment in humans and mice to exacerbate pulmonary hypertension. *J Exp Med.* 2014;211(2):263-80.
4. Chang SH, Yeh YH, Lee JL, Hsu YJ, Kuo CT, and Chen WJ. Transforming growth factor-beta-mediated CD44/STAT3 signaling contributes to the development of atrial fibrosis and fibrillation. *Basic Res Cardiol.* 2017;112(5):58.
5. Moldovan LI, Hansen TB, Veno MT, Okholm TLH, Andersen TL, Hager H, et al. High-throughput RNA sequencing from paired lesional- and non-lesional skin reveals major alterations in the psoriasis circRNAome. *BMC Med Genomics.* 2019;12(1):174.
6. Kristensen LS, Ebbesen KK, Sokol M, Jakobsen T, Korsgaard U, Eriksen AC, et al. Spatial expression analyses of the putative oncogene ciRS-7 in cancer reshape the microRNA sponge theory. *Nat Commun.* 2020;11(1):4551.
7. Sun X, Lin J, Zhang Y, Kang S, Belkin N, Wara AK, et al. MicroRNA-181b Improves Glucose Homeostasis and Insulin Sensitivity by Regulating Endothelial Function in White Adipose Tissue. *Circ Res.* 2016;118(5):810-21.
8. Hayashi M, Nakashima T, Taniguchi M, Kodama T, Kumanogoh A, and Takayanagi H. Osteoprotection by semaphorin 3A. *Nature.* 2012;485(7396):69-74.

9. Hu H, Xuan Y, Xue M, Cheng W, Wang Y, Li X, et al. Semaphorin 3A attenuates cardiac autonomic disorders and reduces inducible ventricular arrhythmias in rats with experimental myocardial infarction. *BMC Cardiovasc Disord.* 2016;16:16.

Supplemental Figures

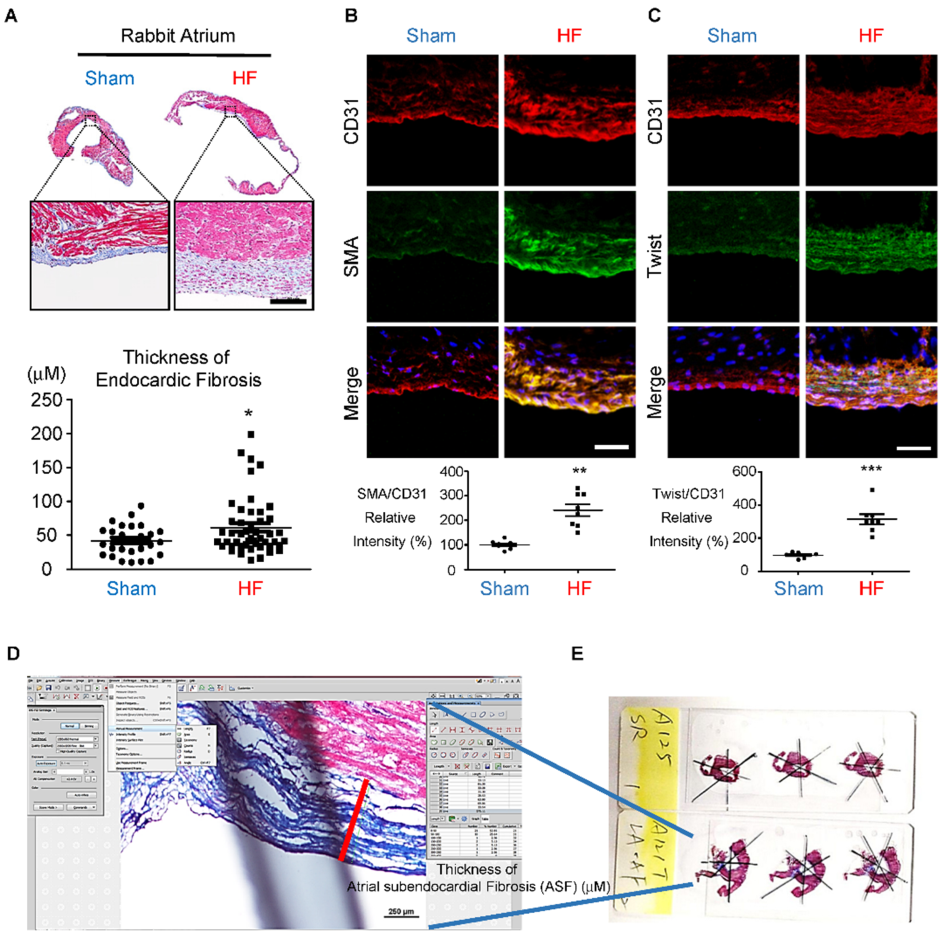

Supplemental Figure 1. Histological analysis of atrial tissue. (A) Rabbit atrial morphology and trichrome staining of the endocardium. Scale bar: 100 µm. Quantitative analysis of endocardial fibrosis thickness. (n= 8). **(B)** Immunohistochemistry analysis of SMA and CD31 and **(C)** Twist and CD31 in the endocardium layer of rabbit heart failure (HF) atria. Quantitation of SMA and Twist expression in the endocardium (CD31). Quantitative analysis of the thickness of atrial subendocardial fibrosis (ASF) (n=8)**. (B-C)** Data are presented as the mean±SEM. *$p<0.05$, **$p<0.01$, ***$p<0.001$ versus sham were obtained by 2-tailed Student's t test. **(D)** Trichrome staining of subendocardial fibrosis shows collagen (blue) deposition in the endocardium. Quantitative analysis of the thickness of collagen deposits by the length of fibrotic area. Thickness of atrial subendocardial fibrosis (ASF) was defined as the distance between the elastic laminae to fibrotic lumen. **(E)** All atrial sections were divided randomly into 6 fields, and trichrome-stained thickness of ASF was measured.

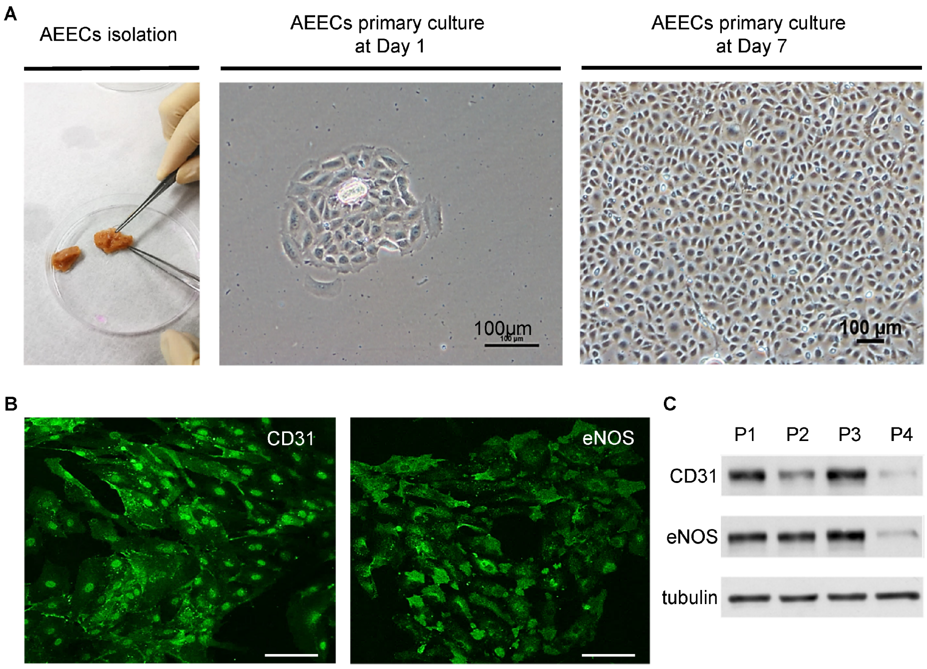

Supplemental Figure 2. Isolation and initial characterization of human AEECs

(A) The AEECs was isolated from AF patients, the phase contrast images show the morphology of AEECs in adherent culture at Day 1, and Day 7. The images were obtained using 20X and 10X objectives. **(B)** The primary culture AEECs were identified by the endothelial-specific markers CD31, and eNOS to characterize the AEECs. **(C).** All endothelial-specific markers are down-regulated at passage 4. Therefore, the AEECs were utilized before passage 4 for the in vitro experiments.

A

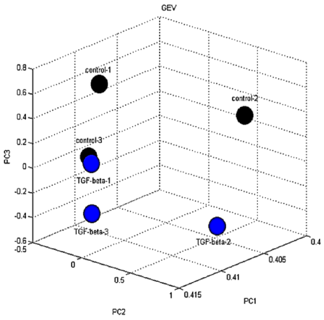

B

Gene Symbol	Gene Description	Fold change
HGF	hepatocyte growth factor	-3.59
SEMA3A	Semaphorin 3a	-2.76
S1PR3	sphingosine-1-phosphate receptor 3	-2.52
LURAP1L	leucine rich adaptor protein 1-like	-1.99
TMTC1	transmembrane and tetratricopeptide repeat containing 1	-1.92
ACSL5	acyl-CoA synthetase long-chain family member 5	-1.89
SHISA3	shisa homolog 3	-1.87
GUCY1A3	guanylate cyclase 1, soluble, alpha 3	-1.84
TNIK	TRAF2 and NCK interacting kinase	-1.79
CCNYL1	cyclin Y-like 1	-1.78

C

```
SEMA3A 3'UTR   5'...CAUGUCCUCUUCCCC--UCAGUGAA...3'
                                     |||||||
miR-181b-3p    3'.........AACGUAAGUAACAAGUCACUC...5'
```

Supplemental Figure 3. Microarray data generation and analysis

(A) AEECs were treated without (black) or with (blue) 5 ng/ml TGF-β and compared by microarray analysis. The three-dimensional projection of the principal component analysis (PCA) shows separation between the two groups. **(B)** Expression profiles of the top 10 downregulated genes related to miR-181b in the hAEEC response to TGF-β. **(C)** Sema3a is a target of miR-181b. Schematic diagram of human Sema3a 3' UTR containing the predicted conserved miR-181b binding site.

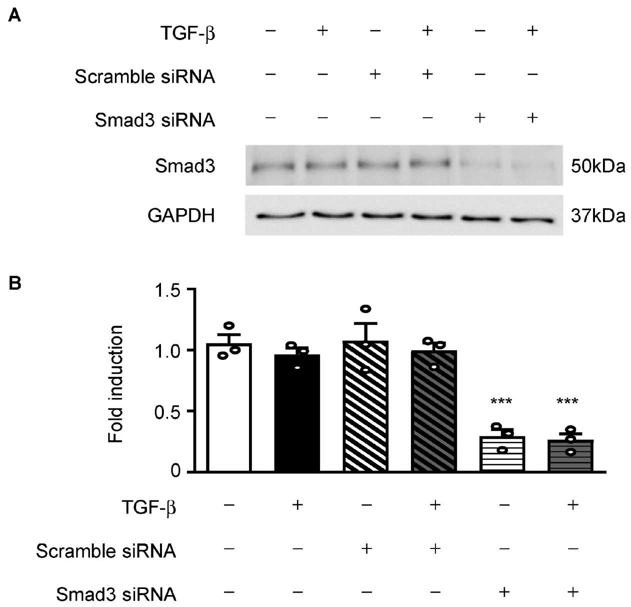

Supplemental Figure 4. Smad3 siRNA knockdown efficiency

AEECs were transfected with Smad3 siRNA or control nontargeting siRNA (scrambled siRNA) and treated with or without TGF-β. Representative immunoblot **(A)** and densitometric quantification **(B)** of protein expression after siRNA transfection. Data are presented as the mean±SEM. (n=3 per group), ***$p<0.001$ versus the scrambled siRNA only group were obtained by 1-way ANOVA with Dunnett's post hoc test.

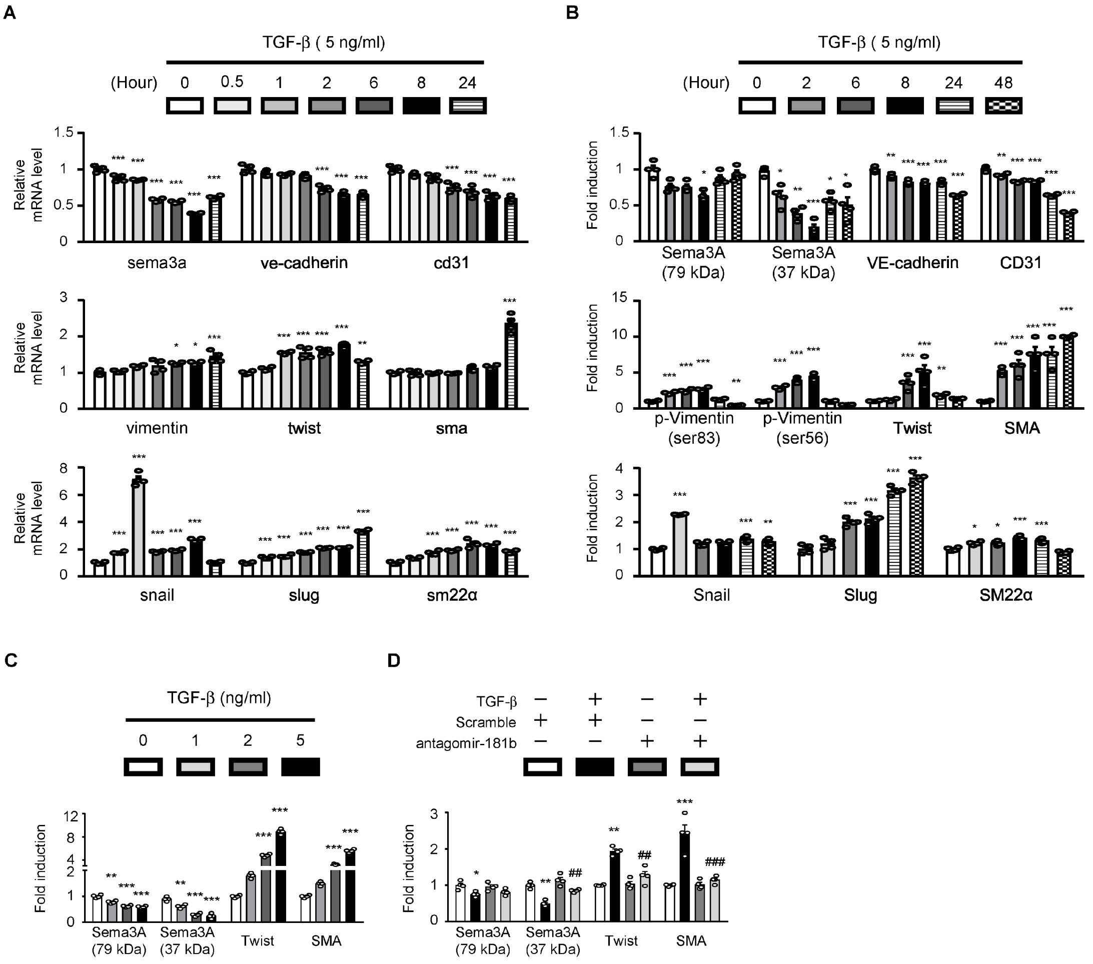

Supplemental Figure 5. The quantitative data of miR-181b targets Sema3a, and TGF-β induces EndMT markers. (A) human AEECs were growth-arrested in starvation medium (0.1% FBS) and then stimulated with 5 ng/ml TGF-β and mRNAs measured by qRT-PCR relative to GAPDH at time points up to 48h. TGF-β decreased sema3a, ve-cadherin, and cd31 and increased EndMT markers in AEECs. GAPDH was used as the loading control. *p<0.05, **p<0.01, ***p<0.001 versus the untreated group were obtained by 1-way ANOVA with Dunnett's post hoc test. **(B)** Representative quantitative data of western immunoblots in AEECs treated with 5 ng/ml TGF-β are showed increased Vimentin, Twist and SMA levels but decreased Sema3A levels at time points up to 48 h. TGF-β increased Twist expression at 8 h and SMA expression at 2 to 48 h and decreased Sema3A expression at 2 to 8 h in AEECs.

GAPDH was used as the loading control. *p<0.05, **p<0.01, ***p<0.001 versus the untreated group were obtained by 1-way ANOVA with Dunnett's post hoc test. **(C)** Representative quantitative data of western immunoblots are shown AEECs were incubated with or without TGF-β (0, 1, 2, or 5 ng/ml). TGF-β increased Twist and SMA at 2 and 5 ng/ml and decreased Sema3A in a dose-dependent manner at 8 h. GAPDH was used as the loading control. *p<0.05, **p<0.01, ***p<0.001 versus the untreated group were obtained by 1-way ANOVA with Dunnett's post hoc test. **(D).** AEECs were transfected with miR-181b antagomir (antagomir-181b) or scrambled control miRNA and then treated with or without TGF-β (5 ng/ml). Representative quantification of protein expression in immunoblot. *p<0.05, **p<0.01, ***p<0.001 versus the scrambled miRNA group without TGF-β; ##p<0.01, ###p<0.001, TGF-β+ scramble group versus the TGF-β+ antagomir-181b group were obtained by 1-way ANOVA with Bonferroni's post hoc test.

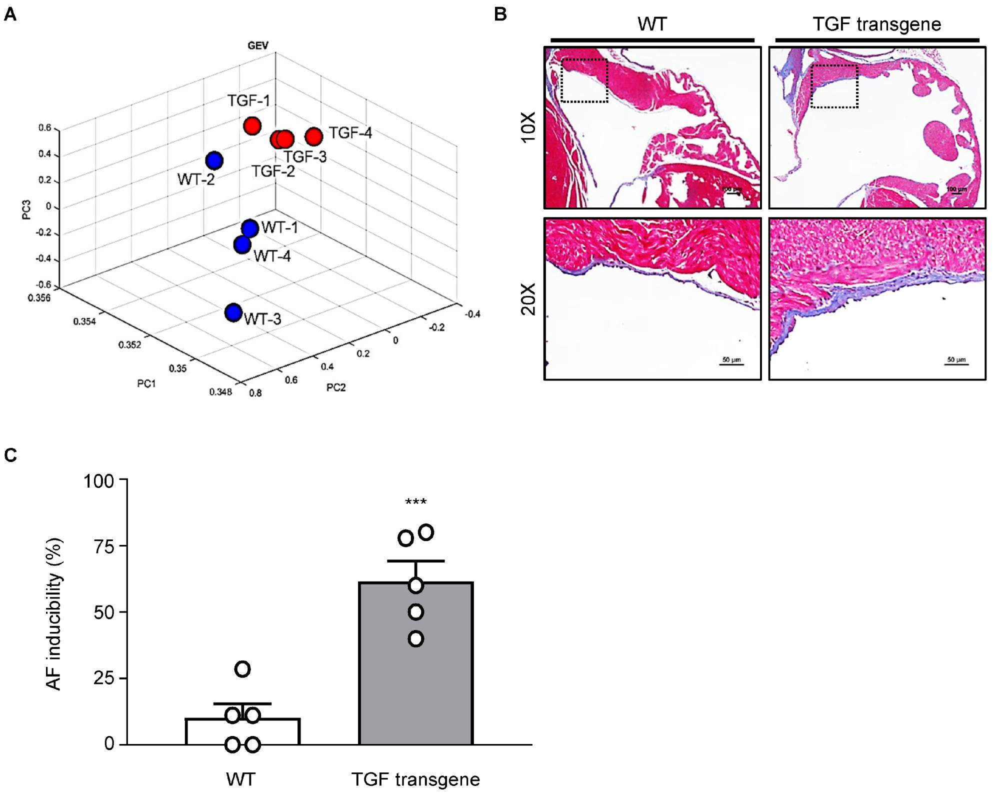

Supplemental Figure 6. Histological analysis of trichrome-stained atrial tissue of transgenic mice with cardiac-specific TGF-β overexpression.

(A) Atrial tissue lysates from TGF-β transgenic mice (Red) and WT mice (blue) were analyzed and compared by next-generation sequencing. The three-dimensional projection of the principal component analysis (PCA) shows separation between the two groups. **(B)** Atrial tissue of TGF-β transgenic mice (TGF transgene) morphology in cross-sections and showed greater collagen (blue) deposition in the endocardium of TGF-β transgenic mice tissue than in that of WT tissue. Scale bar= 100 and 50 µm. **(C)** AF inducibility in TGF-β transgenic mice or WT mice (n=5), ***P<0.001 versus WT were obtained by 2-tailed Student’s t test.

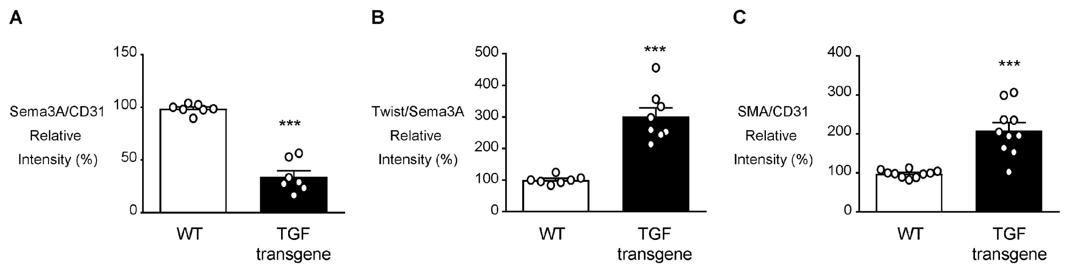

Supplemental Figure 7. Quantitative analyses of immunofluorescent staining for Sema3A, Twist, and SMA contained in CD31+ ECs in the endocardium.

(A) Quantitation of Sema3A and CD31 expression (B) Quantitation of Twist and Sema3A expression, (C) Quantitation of SMA expression and CD31 intensity in the endocardium layer of atria. (**A**-**C**) Data are presented as the mean±SEM (n=10 per group); ***$p<0.001$ versus WT were obtained by 2-tailed Student's t test.

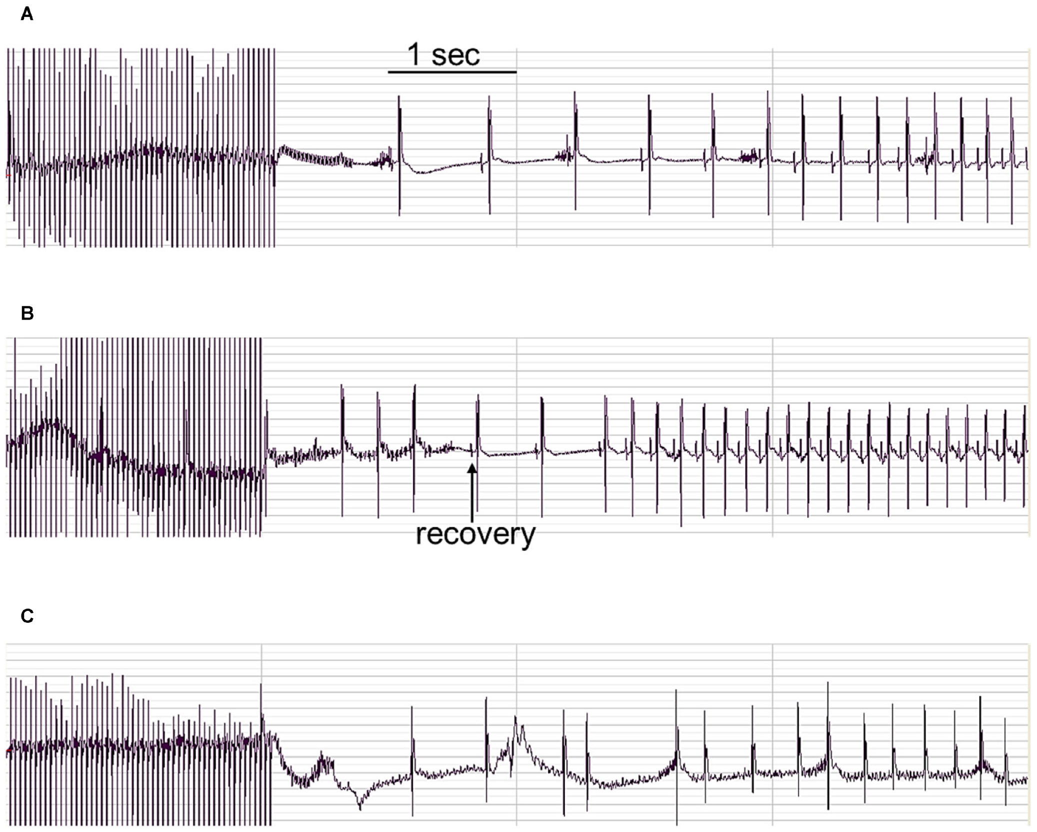

Supplemental Figure 8. Representative EKG tracing after 25-Hz burst pacing: (A) Sinus rhythm, (B) Non-sustained atrial fibrillation (AF), and (C) Sustained AF. The duration of AF was defined as the average of AF duration after burst pacing for 10 times in each mouse. The inducibility of AF was defined as the AF episode lasting for more than 3 seconds after burst pacing for 10 times in each mouse.

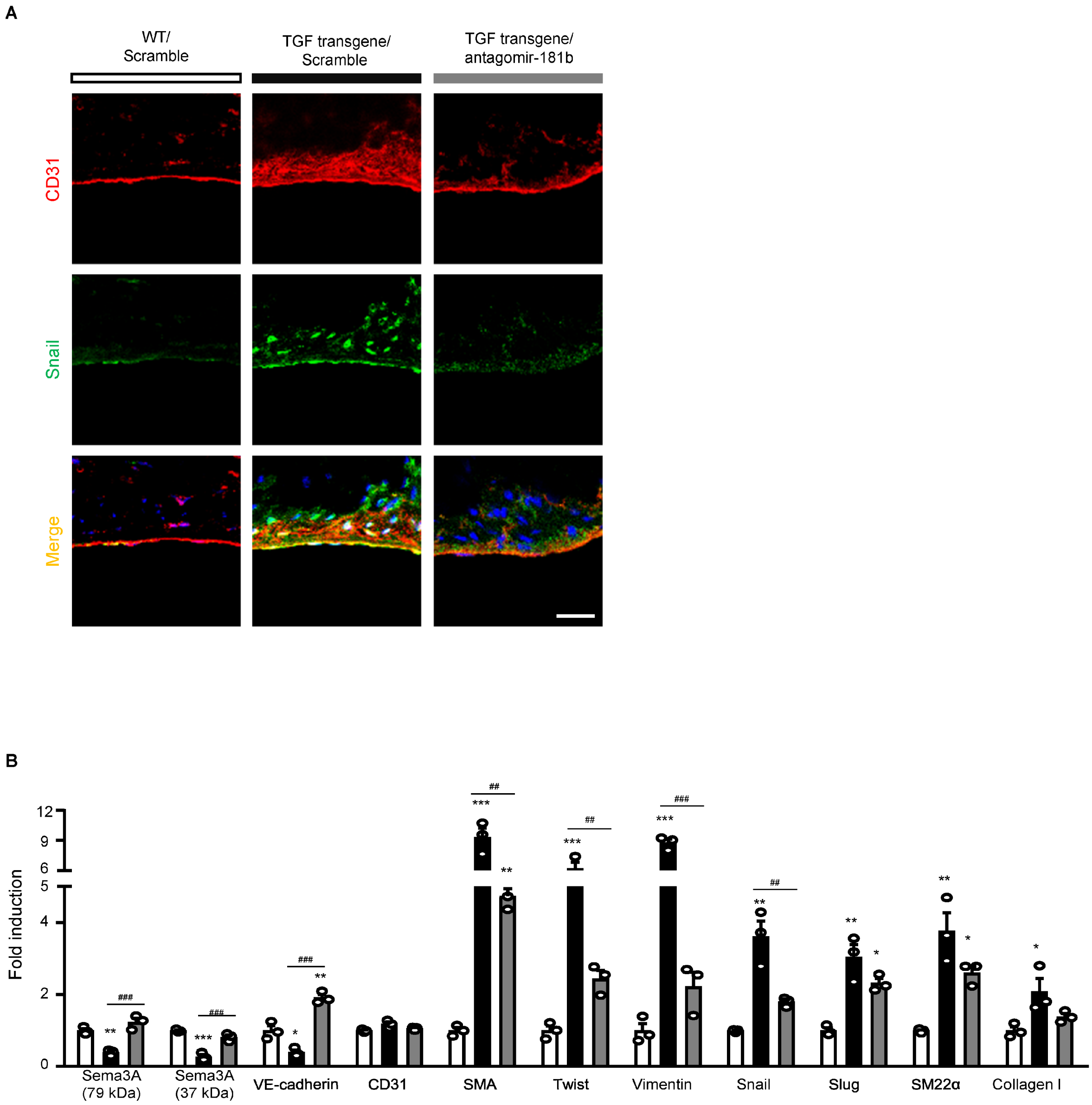

Supplemental Figure 9. The miR-181b antagomir blocks EndMT markers and reverses Sema3A. **(A)** Immunohistochemistry analysis of CD31 with Snail in the endocardium (scale bar: 20 μm; n=5). **(B)** Representative quantitative data of Western blot analysis proteins in atrial tissue from WT, TGF-β transgenic (TGF transgene) and TGF-β transgenic mice (TGF transgene) with antagomir-181b treatment. are showed increased SMA, Twist, Vimentin, Snail, Slug, SM22α and Collagen I levels but decreased Sema3A and VE-cadherin levels in TGF-β transgenic mice group compared with WT. TGF-β transgenic mice with antagomir-181b treatment reverse Sema3A and VE-cadherin protein and reduced EndMT markers. n=3, *p<0.05, **p<0.01, ***p<0.001 versus the WT with scramble group; ##p<0.01, ###p<0.001, TGF-β transgenic mice versus TGF-β transgenic mice with antagomir-181b treatment group were obtained by 1-way ANOVA with Bonferroni's post hoc test.

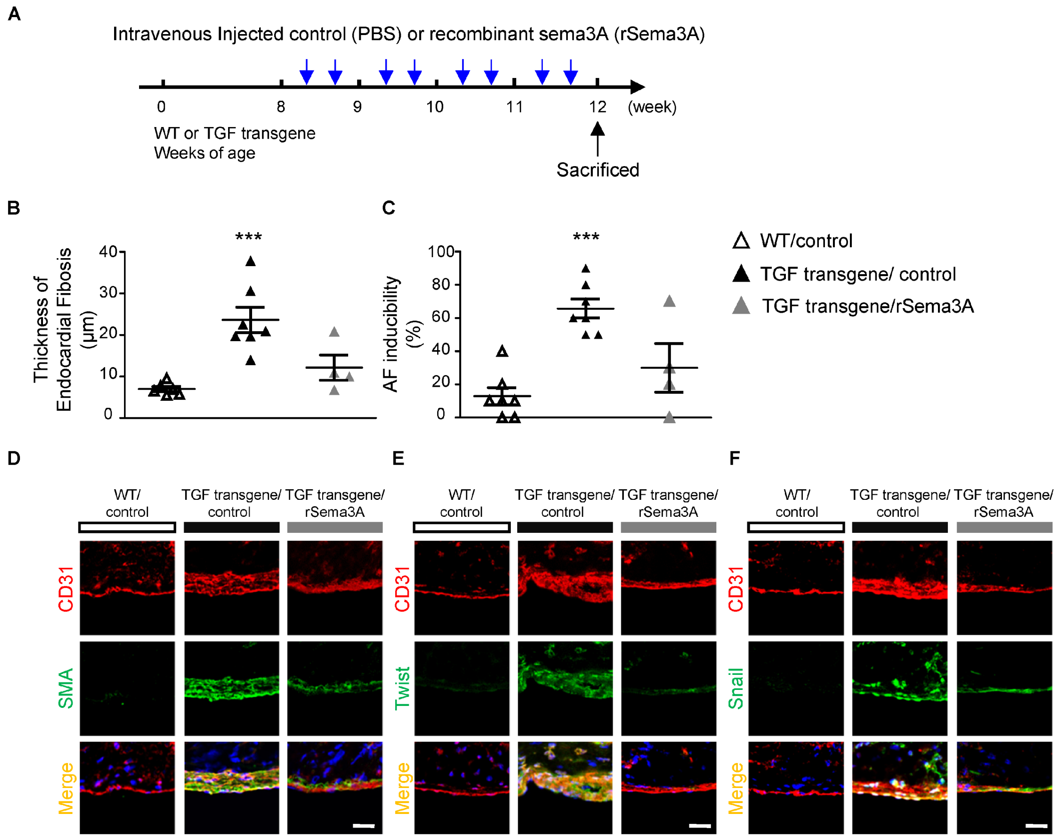

Supplemental Figure 10. Sema3A as a potential AF therapeutic agent

(A) Design and optimization of the appropriate treatment strategy. (B) Quantitative analysis of endocardial fibrosis thickness, (C) AF inducibility. (B-C) Data are presented as the mean±SEM (n= 4-7 per group); ***p<0.001 versus WT/control were obtained by 1-way ANOVA with Bonferroni's post hoc Test. (D) (E) (F) Immunohistochemistry analysis of CD31 with SMA, Twist and Snail in the endocardium (scale bar: 20 μm; n=4-5).

Supplemental table. 1 the gene list from the Human Transcriptome Array 2.0 data

GeneSymbol	Gene Description	Fold change	p value
ADH1B	alcohol dehydrogenase 1B (class I), beta polypeptide	-20.44	0.026
ADAMTS1	ADAM metallopeptidase with thrombospondin type 1 motif, 1	-3.81	0.017
ADAMTS9	ADAM metallopeptidase with thrombospondin type 1 motif, 9	-3.75	0.045
HGF	hepatocyte growth factor (hepapoietin A; scatter factor)	-3.59	0.004
SPRY1	sprouty homolog 1, antagonist of FGF signaling (Drosophila)	-3.39	0.035
PCDH18	protocadherin 18	-3.35	0.049
TMEM88	transmembrane protein 88	-2.82	0.024
TRIM25	tripartite motif containing 25	-2.80	0.029
CYGB	cytoglobin	-2.78	0.027
SEMA3A	sema domain, immunoglobulin domain (Ig), short basic domain, secreted, (semaphorin) 3A	-2.76	0.004
IFI6	interferon, alpha-inducible protein 6	-2.57	0.015
ALDH1A3	aldehyde dehydrogenase 1 family, member A3	-2.54	0.028
S1PR3	sphingosine-1-phosphate receptor 3	-2.52	0.012
MAP3K5	mitogen-activated protein kinase kinase kinase 5	-2.40	0.040
KITLG	KIT ligand	-2.34	0.026
MYRIP	myosin VIIA and Rab interacting protein	-2.34	0.046
FGL2	fibrinogen-like 2	-2.33	0.033
PHLDA1	pleckstrin homology-like domain, family A, member 1	-2.33	0.038
SMAD3	SMAD family member 3	-2.31	0.023
NPY1R	neuropeptide Y receptor Y1	-2.27	0.007

CITED2	Cbp/p300-interacting transactivator, with Glu/Asp-rich carboxy-terminal domain, 2	-2.24	0.027
EDNRB	endothelin receptor type B	-2.22	0.039
TNFAIP3	tumor necrosis factor, alpha-induced protein 3	-2.21	0.015
ZFP36	ZFP36 ring finger protein	-2.20	0.025
EBF2	early B-cell factor 2	-2.14	0.039
CNKSR3	CNKSR family member 3	-2.13	0.038
HSPB6	heat shock protein, alpha-crystallin-related, B6	-2.12	0.046
RNF144B	ring finger protein 144B	-2.08	0.005
UST	uronyl-2-sulfotransferase	-2.07	0.024
PIEZO2	piezo-type mechanosensitive ion channel component 2	-2.06	0.004
CCDC102B	coiled-coil domain containing 102B	-2.04	0.021
OAS3	2'-5'-oligoadenylate synthetase 3, 100kDa	-2.02	0.019
GGT5	gamma-glutamyltransferase 5	-2.01	0.042
MX1	myxovirus (influenza virus) resistance 1, interferon-inducible protein p78 (mouse)	-2.01	0.031
SQRDL	sulfide quinone reductase-like (yeast)	-2.00	0.017
SAMHD1	SAM domain and HD domain 1	-2.00	0.049
HIST1H2BM	histone cluster 1, H2bm	-2.00	0.011
FLOT1	flotillin 1	-2.00	0.024
GUCY1B3	guanylate cyclase 1, soluble, beta 3	-1.99	0.030
LURAP1L	leucine rich adaptor protein 1-like	-1.99	0.016
EPDR1	ependymin related protein 1 (zebrafish)	-1.98	0.001
ADH1C	alcohol dehydrogenase 1C (class I), gamma polypeptide	-1.98	0.030
CTSL1	cathepsin L1	-1.96	0.026

IFITM4P	interferon induced transmembrane protein 4 pseudogene	-1.94	0.035
LRIG3	leucine-rich repeats and immunoglobulin-like domains 3	-1.94	0.037
IL13RA2	interleukin 13 receptor, alpha 2	-1.93	0.023
TCN2	transcobalamin II	-1.92	0.049
TMTC1	transmembrane and tetratricopeptide repeat containing 1	-1.92	0.020
EPS8	epidermal growth factor receptor pathway substrate 8	-1.91	0.026
APOBEC3B	apolipoprotein B mRNA editing enzyme, catalytic polypeptide-like 3B	-1.90	0.020
PTGER4	prostaglandin E receptor 4 (subtype EP4)	-1.90	0.030
ID4	inhibitor of DNA binding 4, dominant negative helix-loop-helix protein	-1.90	0.000
PDLIM1	PDZ and LIM domain 1	-1.90	0.001
EPSTI1	epithelial stromal interaction 1 (breast)	-1.89	0.003
ACSL5	acyl-CoA synthetase long-chain family member 5	-1.89	0.015
APOBEC3D	apolipoprotein B mRNA editing enzyme, catalytic polypeptide-like 3D	-1.89	0.023
SHISA3	shisa homolog 3 (Xenopus laevis)	-1.87	0.047
TNFRSF1B	tumor necrosis factor receptor superfamily, member 1B	-1.87	0.029
FAM129A	family with sequence similarity 129, member A	-1.86	0.037
KCTD12	potassium channel tetramerisation domain containing 12	-1.86	0.007
CDCA7L	cell division cycle associated 7-like	-1.85	0.001
GATA4	GATA binding protein 4	-1.85	0.009
FLOT2	flotillin 2	-1.84	0.015
GUCY1A3	guanylate cyclase 1, soluble, alpha 3	-1.84	0.019
SAT1	spermidine/spermine N1-acetyltransferase 1	-1.83	0.012

NID2	nidogen 2 (osteonidogen)	-1.83	0.042
NID1	nidogen 1	-1.81	0.040
ATP2B4	ATPase, Ca++ transporting, plasma membrane 4	-1.81	0.011
ALDH6A1	aldehyde dehydrogenase 6 family, member A1	-1.81	0.034
USP18	ubiquitin specific peptidase 18	-1.80	0.006
BLVRB	biliverdin reductase B (flavin reductase (NADPH))	-1.79	0.047
TNIK	TRAF2 and NCK interacting kinase	-1.79	0.022
CCNB2	cyclin B2	-1.78	0.036
CCNYL1	cyclin Y-like 1	-1.78	0.040
ATP5D	ATP synthase, H+ transporting, mitochondrial F1 complex, delta subunit	-1.78	0.011
HCP5	HLA complex P5 (non-protein coding)	-1.77	0.036
PLA2G16	phospholipase A2, group XVI	-1.77	0.031
RAPH1	Ras association (RalGDS/AF-6) and pleckstrin homology domains 1	-1.77	0.009
TMEM106C	transmembrane protein 106C	-1.74	0.042
ADIRF	adipogenesis regulatory factor	-1.74	0.049
APOBEC3C	apolipoprotein B mRNA editing enzyme, catalytic polypeptide-like 3C	-1.74	0.013
ADH1A	alcohol dehydrogenase 1A (class I), alpha polypeptide	-1.72	0.029
RAC2	ras-related C3 botulinum toxin substrate 2 (rho family, small GTP binding protein Rac2)	-1.72	0.030
TBC1D8	TBC1 domain family, member 8 (with GRAM domain)	-1.71	0.024
GIPC2	GIPC PDZ domain containing family, member 2	-1.70	0.027
HIST1H2BI	histone cluster 1, H2bi	-1.70	0.045

SNHG8	small nucleolar RNA host gene 8 (non-protein coding)	-1.69	0.014
RPS3AP47	ribosomal protein S3a pseudogene 47	-1.69	0.004
CA12	carbonic anhydrase XII	-1.68	0.029
SLIT2	slit homolog 2 (Drosophila)	-1.68	0.029
STEAP1B	STEAP family member 1B	-1.68	0.034
COLEC12	collectin sub-family member 12	-1.68	0.039
ZNF366	zinc finger protein 366	-1.68	0.004
ICAM2	intercellular adhesion molecule 2	-1.67	0.023
GSTM4	glutathione S-transferase mu 4	-1.67	0.049
EVA1C	eva-1 homolog C (C. elegans)	-1.66	0.001
TJP2	tight junction protein 2	-1.66	0.002
GPM6B	glycoprotein M6B	-1.66	0.012
FNBP1	formin binding protein 1	-1.66	0.034
CDK4	cyclin-dependent kinase 4	-1.65	0.011
RNU5F-1	RNA, U5F small nuclear 1	-1.65	0.021
EPHX1	epoxide hydrolase 1, microsomal (xenobiotic)	-1.65	0.028
C19orf54	chromosome 19 open reading frame 54	-1.65	0.036
FLI1-AS1	FLI1 antisense RNA 1	-1.64	0.037
SNAI2	snail family zinc finger 2	-1.64	0.028
FOSL1	FOS-like antigen 1	-1.64	0.024
IFITM2	interferon induced transmembrane protein 2	-1.64	0.046
PLCE1-AS1	PLCE1 antisense RNA 1	-1.64	0.030
SNORD14D	small nucleolar RNA, C/D box 14D	-1.64	0.023
AK5	adenylate kinase 5	-1.64	0.008
PNPO	pyridoxamine 5'-phosphate oxidase	-1.63	0.025

DUSP6	dual specificity phosphatase 6	-1.63	0.023
KAT2B	K(lysine) acetyltransferase 2B	-1.63	0.003
ZCCHC2	zinc finger, CCHC domain containing 2	-1.62	0.003
LDB2	LIM domain binding 2	-1.62	0.025
IL18R1	interleukin 18 receptor 1	-1.62	0.012
APOL6	apolipoprotein L, 6	-1.62	0.034
LMO3	LIM domain only 3 (rhombotin-like 2)	-1.61	0.013
NFKBIA	nuclear factor of kappa light polypeptide gene enhancer in B-cells inhibitor, alpha	-1.61	0.021
SLC43A3	solute carrier family 43, member 3	-1.61	0.021
IRF2	interferon regulatory factor 2	-1.61	0.042
SLCO2A1	solute carrier organic anion transporter family, member 2A1	-1.61	0.037
RNF213	ring finger protein 213	-1.61	0.030
UCP2	uncoupling protein 2 (mitochondrial, proton carrier)	-1.60	0.044
IL33	interleukin 33	-1.60	0.000
CD34	CD34 molecule	-1.60	0.009
HIST1H3H	histone cluster 1, H3h	-1.60	0.016
PSMB9	proteasome (prosome, macropain) subunit, beta type, 9	-1.60	0.036
PSMB9	proteasome (prosome, macropain) subunit, beta type, 9	-1.60	0.036
TYMS	thymidylate synthetase	-1.60	0.050
PSMB9	proteasome (prosome, macropain) subunit, beta type, 9	-1.60	0.035
PAG1	phosphoprotein associated with glycosphingolipid microdomains 1	-1.60	0.030
CASP10	caspase 10, apoptosis-related cysteine peptidase	-1.59	0.032
AHNAK	AHNAK nucleoprotein	-1.59	0.039

TPX2	TPX2, microtubule-associated, homolog (Xenopus laevis)	-1.59	0.030
TSPAN18	tetraspanin 18	-1.59	0.047
CTC1	CTS telomere maintenance complex component 1	-1.58	0.016
IL1RL1	interleukin 1 receptor-like 1	-1.58	0.023
SVILP1	supervillin pseudogene 1	-1.58	0.034
AIM1	absent in melanoma 1	-1.57	0.028
FERMT3	fermitin family member 3	-1.57	0.038
ITPK1	inositol-tetrakisphosphate 1-kinase	-1.57	0.048
MTRNR2L1	MT-RNR2-like 1	-1.56	0.006
ETS2	v-ets erythroblastosis virus E26 oncogene homolog 2 (avian)	-1.56	0.049
ARL2	ADP-ribosylation factor-like 2	-1.56	0.003
CAMK1	calcium/calmodulin-dependent protein kinase I	-1.56	0.001
FIG4	FIG4 homolog, SAC1 lipid phosphatase domain containing (S. cerevisiae)	-1.56	0.041
MAFB	v-maf musculoaponeurotic fibrosarcoma oncogene homolog B (avian)	-1.55	0.013
ADAM15	ADAM metallopeptidase domain 15	-1.55	0.009
CD47	CD47 molecule	-1.55	0.006
SRPX	sushi-repeat containing protein, X-linked	-1.55	0.036
TRERF1	transcriptional regulating factor 1	-1.55	0.007
FGD5	FYVE, RhoGEF and PH domain containing 5	-1.54	0.026
OSR1	odd-skipped related 1 (Drosophila)	-1.54	0.004
LIN7A	lin-7 homolog A (C. elegans)	-1.54	0.004
REPS1	RALBP1 associated Eps domain containing 1	-1.54	0.025
LOC100129518	uncharacterized LOC100129518	-1.53	0.034

SLC50A1	solute carrier family 50 (sugar transporter), member 1	-1.53	0.026
LOC729603	calcium binding protein P22 pseudogene	-1.52	0.039
ARHGEF3	Rho guanine nucleotide exchange factor (GEF) 3	-1.52	0.013
ZNF319	zinc finger protein 319	-1.52	0.024
HIST3H2A	histone cluster 3, H2a	-1.52	0.044
USP41	ubiquitin specific peptidase 41	-1.52	0.008
FTL	ferritin, light polypeptide	-1.52	0.041
SMAP2	small ArfGAP2	-1.51	0.038
MMAB	methylmalonic aciduria (cobalamin deficiency) cblB type	-1.51	0.015
SLC7A8	solute carrier family 7 (amino acid transporter light chain, L system), member 8	-1.51	0.047
HSBP1L1	heat shock factor binding protein 1-like 1	-1.51	0.012
MGST1	microsomal glutathione S-transferase 1	-1.51	0.007
OXA1L	oxidase (cytochrome c) assembly 1-like	-1.50	0.031
CLEC14A	C-type lectin domain family 14, member A	-1.50	0.006
ARHGAP6	Rho GTPase activating protein 6	-1.50	0.030
ANKRD10-IT1	ANKRD10 intronic transcript 1 (non-protein coding)	1.50	0.018
SEC23A	Sec23 homolog A (S. cerevisiae)	1.50	0.047
GEM	GTP binding protein overexpressed in skeletal muscle	1.50	0.007
RALA	v-ral simian leukemia viral oncogene homolog A (ras related)	1.50	0.020
SLFN5	schlafen family member 5	1.51	0.002
PACS1	phosphofurin acidic cluster sorting protein 1	1.51	0.033
ACBD3	acyl-CoA binding domain containing 3	1.52	0.015
RNU7-37P	RNA, U7 small nuclear 37 pseudogene	1.52	0.035
ZNF432	zinc finger protein 432	1.52	0.002

MYO1D	myosin ID	1.53	0.006
SIPA1L3	signal-induced proliferation-associated 1 like 3	1.53	0.044
ZNF460	zinc finger protein 460	1.54	0.045
BMS1	BMS1 ribosome biogenesis factor	1.54	0.047
MT1L	metallothionein 1L (gene/pseudogene)	1.54	0.006
MMP16	matrix metallopeptidase 16 (membrane-inserted)	1.54	0.037
KIF3A	kinesin family member 3A	1.55	0.046
MEG3	maternally expressed 3 (non-protein coding)	1.55	0.023
DST	dystonin	1.56	0.048
GALNT7	UDP-N-acetyl-alpha-D-galactosamine:polypeptide N-acetylgalactosaminyltransferase 7 (GalNAc-T7)	1.57	0.008
ITGA5	integrin, alpha 5 (fibronectin receptor, alpha polypeptide)	1.57	0.048
ZDHHC17	zinc finger, DHHC-type containing 17	1.58	0.044
UGGT2	UDP-glucose glycoprotein glucosyltransferase 2	1.59	0.035
IFNE	interferon, epsilon	1.59	0.001
SNORD113-4	small nucleolar RNA, C/D box 113-4	1.60	0.026
DLC1	deleted in liver cancer 1	1.60	0.018
RAB23	RAB23, member RAS oncogene family	1.60	0.043
FBXO32	F-box protein 32	1.61	0.048
APBB2	amyloid beta (A4) precursor protein-binding, family B, member 2	1.61	0.049
INSIG2	insulin induced gene 2	1.61	0.033
CTHRC1	collagen triple helix repeat containing 1	1.61	0.030
CCDC176	coiled-coil domain containing 176	1.62	0.029
LINC00842	long intergenic non-protein coding RNA 842	1.62	0.019

ACSL4	acyl-CoA synthetase long-chain family member 4	1.62	0.016
SMYD3-IT1	SMYD3 intronic transcript 1 (non-protein coding)	1.63	0.046
ASAH2B	N-acylsphingosine amidohydrolase (non-lysosomal ceramidase) 2B	1.63	0.002
EDEM3	ER degradation enhancer, mannosidase alpha-like 3	1.63	0.046
UBL3	ubiquitin-like 3	1.64	0.020
SNX25	sorting nexin 25	1.65	0.004
VDR	vitamin D (1,25- dihydroxyvitamin D3) receptor	1.65	0.010
HIVEP2	human immunodeficiency virus type I enhancer binding protein 2	1.66	0.002
COL5A2	collagen, type V, alpha 2	1.66	0.035
SNORD64	small nucleolar RNA, C/D box 64	1.67	0.003
DGKI	diacylglycerol kinase, iota	1.68	0.006
ABL2	v-abl Abelson murine leukemia viral oncogene homolog 2	1.68	0.005
KLHL28	kelch-like family member 28	1.68	0.038
BIRC2	baculoviral IAP repeat containing 2	1.69	0.045
ARL5A	ADP-ribosylation factor-like 5A	1.69	0.038
SMURF2	SMAD specific E3 ubiquitin protein ligase 2	1.70	0.022
SLC39A6	solute carrier family 39 (zinc transporter), member 6	1.71	0.044
FZD8	frizzled family receptor 8	1.71	0.002
TMC7	transmembrane channel-like 7	1.71	0.020
TULP4	tubby like protein 4	1.71	0.009
COG6	component of oligomeric golgi complex 6	1.71	0.034
TTC3P1	tetratricopeptide repeat domain 3 pseudogene 1	1.73	0.043
ZBTB38	zinc finger and BTB domain containing 38	1.73	0.033

DLG1	discs, large homolog 1 (Drosophila)	1.73	0.006
OCIAD2	OCIA domain containing 2	1.75	0.015
FNDC3B	fibronectin type III domain containing 3B	1.75	0.043
AEBP1	AE binding protein 1	1.75	0.012
RAI14	retinoic acid induced 14	1.75	0.035
CARD11	caspase recruitment domain family, member 11	1.76	0.035
NYNRIN	NYN domain and retroviral integrase containing	1.76	0.021
RNF121	ring finger protein 121	1.76	0.009
USP46	ubiquitin specific peptidase 46	1.77	0.012
PDGFB	platelet-derived growth factor beta polypeptide	1.77	0.033
EVI5	ecotropic viral integration site 5	1.78	0.045
HOMER1	homer homolog 1 (Drosophila)	1.78	0.027
ELL2	elongation factor, RNA polymerase II, 2	1.78	0.028
PANX1	pannexin 1	1.79	0.030
RNF152	ring finger protein 152	1.80	0.016
TMEM45A	transmembrane protein 45A	1.80	0.020
ITGA2	integrin, alpha 2 (CD49B, alpha 2 subunit of VLA-2 receptor)	1.80	0.037
KIAA1033	KIAA1033	1.81	0.031
LINC00152	long intergenic non-protein coding RNA 152	1.81	0.034
ALDH1L2	aldehyde dehydrogenase 1 family, member L2	1.81	0.031
FNDC3A	fibronectin type III domain containing 3A	1.81	0.023
PCDH9	protocadherin 9	1.85	0.012
C15orf54	chromosome 15 open reading frame 54	1.85	0.022
HSPA13	heat shock protein 70kDa family, member 13	1.85	0.047

CEP170	centrosomal protein 170kDa	1.85	0.030
SAMD9	sterile alpha motif domain containing 9	1.86	0.044
PPP2R3A	protein phosphatase 2, regulatory subunit B", alpha	1.86	0.004
CDK6	cyclin-dependent kinase 6	1.87	0.022
LOX	lysyl oxidase	1.87	0.021
GOLIM4	golgi integral membrane protein 4	1.88	0.039
RAB3B	RAB3B, member RAS oncogene family	1.89	0.050
LOXL3	lysyl oxidase-like 3	1.90	0.007
MYO10	myosin X	1.90	0.018
TBX3	T-box 3	1.92	0.031
ZSWIM6	zinc finger, SWIM-type containing 6	1.93	0.014
BMPR2	bone morphogenetic protein receptor, type II (serine/threonine kinase)	1.93	0.036
SRPX2	sushi-repeat containing protein, X-linked 2	1.93	0.002
LINC00340	long intergenic non-protein coding RNA 340	1.95	0.038
C8orf4	chromosome 8 open reading frame 4	1.96	0.025
CSGALNACT2	chondroitin sulfate N-acetylgalactosaminyltransferase 2	1.97	0.009
CDK14	cyclin-dependent kinase 14	1.99	0.021
NT5DC2	5'-nucleotidase domain containing 2	2.00	0.027
CEP170P1	centrosomal protein 170kDa pseudogene 1	2.00	0.036
WNT5B	wingless-type MMTV integration site family, member 5B	2.01	0.029
SNORA11	small nucleolar RNA, H/ACA box 11	2.01	0.003
COL1A1	collagen, type I, alpha 1	2.01	0.040
LPCAT2	lysophosphatidylcholine acyltransferase 2	2.02	0.020
SNORD114-17	small nucleolar RNA, C/D box 114-17	2.02	0.019

TBC1D8B	TBC1 domain family, member 8B (with GRAM domain)	2.03	0.040
LRP1	low density lipoprotein receptor-related protein 1	2.04	0.045
FRMD6	FERM domain containing 6	2.05	0.002
SPSB1	splA/ryanodine receptor domain and SOCS box containing 1	2.07	0.047
PTPRK	protein tyrosine phosphatase, receptor type, K	2.07	0.003
GRPEL2	GrpE-like 2, mitochondrial (E. coli)	2.08	0.041
KRT7	keratin 7	2.15	0.004
KLF7-IT1	KLF7 intronic transcript 1 (non-protein coding)	2.16	0.048
COL4A1	collagen, type IV, alpha 1	2.18	0.023
LIMS3L	LIM and senescent cell antigen-like domains 3-like	2.18	0.033
PCDH10	protocadherin 10	2.19	0.028
VCAN-AS1	VCAN antisense RNA 1	2.22	0.045
TTC3	tetratricopeptide repeat domain 3	2.30	0.046
MFAP5	microfibrillar associated protein 5	2.37	0.049
MEDAG	mesenteric estrogen-dependent adipogenesis	2.39	0.024
NEGR1	neuronal growth regulator 1	2.39	0.040
IGFBP3	insulin-like growth factor binding protein 3	2.39	0.020
MIR181A2HG	MIR181A2 host gene (non-protein coding)	2.41	0.045
SNORD114-2	small nucleolar RNA, C/D box 114-2	2.43	0.040
LINC00312	long intergenic non-protein coding RNA 312	2.46	0.048
RNA5SP385	RNA, 5S ribosomal pseudogene 385	2.49	0.002
PTGER2	prostaglandin E receptor 2 (subtype EP2), 53kDa	2.49	0.033
UBA6	ubiquitin-like modifier activating enzyme 6	2.50	0.048
YIPF5	Yip1 domain family, member 5	2.51	0.033
SEMA3C	sema domain, immunoglobulin domain (Ig), short basic	2.53	0.017

	domain, secreted, (semaphorin) 3C		
TNS1	tensin 1	2.58	0.005
TPM1	tropomyosin 1 (alpha)	2.59	0.025
ESM1	endothelial cell-specific molecule 1	2.65	0.050
WNT5A	wingless-type MMTV integration site family, member 5A	2.66	0.039
PLCB4	phospholipase C, beta 4	2.69	0.050
ITGBL1	integrin, beta-like 1 (with EGF-like repeat domains)	2.73	0.001
GRIA3	glutamate receptor, ionotropic, AMPA 3	2.78	0.022
RHOB	ras homolog family member B	2.79	0.004
MBOAT2	membrane bound O-acyltransferase domain containing 2	2.83	0.012
PCDHGC5	protocadherin gamma subfamily C, 5	2.88	0.014
TNFAIP6	tumor necrosis factor, alpha-induced protein 6	2.90	0.038
SPOCK1	sparc/osteonectin, cwcv and kazal-like domains proteoglycan (testican) 1	2.91	0.018
ADAMTS6	ADAM metallopeptidase with thrombospondin type 1 motif, 6	2.92	0.042
VEGFA	vascular endothelial growth factor A	2.97	0.018
ITGAV	integrin, alpha V	2.97	0.023
SLC19A2	solute carrier family 19 (thiamine transporter), member 2	3.03	0.009
WISP1	WNT1 inducible signaling pathway protein 1	3.07	0.034
PGM2L1	phosphoglucomutase 2-like 1	3.08	0.033
LIPG	lipase, endothelial	3.11	0.031
SERPINE2	serpin peptidase inhibitor, clade E (nexin, plasminogen activator inhibitor type 1), member 2	3.12	0.008
PLXDC2	plexin domain containing 2	3.13	0.040

LMCD1	LIM and cysteine-rich domains 1	3.23	0.026
PALLD	palladin, cytoskeletal associated protein	3.34	0.013
PPP1R14C	protein phosphatase 1, regulatory (inhibitor) subunit 14C	3.43	0.029
PDPN	podoplanin	3.44	0.044
DACT1	dishevelled-binding antagonist of beta-catenin 1	3.48	0.019
DPYSL3	dihydropyrimidinase-like 3	3.50	0.050
PDGFC	platelet derived growth factor C	3.70	0.024
FBLN5	fibulin 5	3.79	0.003
ANGPTL4	angiopoietin-like 4	3.81	0.034
BHLHE40	basic helix-loop-helix family, member e40	3.82	0.021
NOX4	NADPH oxidase 4	3.86	0.004
CDH2	cadherin 2, type 1, N-cadherin (neuronal)	3.89	0.003
C5orf46	chromosome 5 open reading frame 46	3.95	0.031
LTBP2	latent transforming growth factor beta binding protein 2	4.09	0.005
SEMA7A	semaphorin 7A, GPI membrane anchor (John Milton Hagen blood group)	4.13	0.018
STK38L	serine/threonine kinase 38 like	4.17	0.017
FAP	fibroblast activation protein, alpha	4.27	0.009
PMEPA1	prostate transmembrane protein, androgen induced 1	4.34	0.043
CDKN2B	cyclin-dependent kinase inhibitor 2B (p15, inhibits CDK4)	4.99	0.035
SLC46A3	solute carrier family 46, member 3	4.99	0.020
XYLT1	xylosyltransferase I	5.23	0.023
TSPAN13	tetraspanin 13	5.77	0.022
ADAM12	ADAM metallopeptidase domain 12	6.43	0.009
SKIL	SKI-like oncogene	6.56	0.013

CDH6	cadherin 6, type 2, K-cadherin (fetal kidney)	6.77	0.003
TGFBI	transforming growth factor, beta-induced, 68kDa	7.05	0.002

Supplemental table 2. Sequencing summary of the small RNA libraries. Raw reads were quality processed and aligned to the reference mouse genome mm10 and miRBase (miRBase22) (TGF-β transgenic mouse=TGF). Total number and the percentage of processed reads mapped to miRNA is shown. The number of miRNAs identified in each sample with **≥1 or ≥3** read counts are indicated

**Sample**	**TGF 1**	**TGF 2**	**TGF 3**	**TGF 4**	**WT 1**	**WT 2**	**WT 3**	**WT 4**
Total Raw Reads	8901145	26028620	27046765	16157349	13532478	16001879	27101615	20852973
Avg. read length	20.8328	21.2732	21.1261	19.7869	19.5943	19.7583	21.2419	21.0306
Reads after pre-processing	8572099	24888731	25517586	15315321	12806417	15185028	26228520	19853251
Aligned (%)	96.30	95.62	94.35	94.79	94.63	94.90	96.78	95.21
Fully within a microRNA	16.84%	35.16%	27.01%	16.16%	16.29%	18.47%	37.36%	30.94%
Partly within a microRNA	5.72%	11.38%	9.22%	5.56%	5.33%	6.11%	13.19%	10.94%
Not in a microRNA	77.45%	53.46%	63.78%	78.28%	78.39%	75.42%	49.45%	58.12%
microRNA reads	1443541.472	8750877.82	6892299.979	2474955.874	2086165.33	2804674.67	9798975.07	6142595.9
Total expressed miRNA (1or more reads)	423	429	442	434	411	428	412	412
miRNA (3 or more reads)	362	347	358	356	351	346	327	331

Uncuted bloting

MiR-181b targets semaphorin 3a to mediate TGF-β-induced endothelial-to-mesenchymal transition related to atrial fibrillation

Running title: *MiR-181b is associated with TGF-β-induced EndMT in AF*

Ying-Ju Lai[1,2,3#], Feng-Chun Tsai[4, 5#], Gwo-Jyh Chang[1,6], Shang-Hung Chang,[1,5], Chung-Chi Huang, [2,7], Wei-Jan Chen,[1,5]*, Yung-Hsin Yeh[1,5]*

[1]Cardiovascular Department, Chang Gung Memorial Hospital, Tao-Yuan, [2]Department of Respiratory Therapy, Chang Gung University College of Medicine, Tao-Yuan. [3]Department of Respiratory Care, Chang Gung University of Science and Technology, Chia-Yi. [4]Department of Thoracic and Cardiovascular Surgery, Chang Gung Memorial Hospital, Tao-Yuan. [5]Department of medicine, Chang Gung University College of Medicine, [6]Graduate Institute of Clinical Medical Sciences, Chang Gung University College of Medicine, [7] Department of Pulmonary and Critical Care Medicine, Chang Gung Memorial Hospital, Tao-Yuan, Taiwan

The authors have declared that no conflict of interest exists.

Address for Correspondence: Yung-Hsin Yeh, MD
The Cardiovascular Department, Chang Gung Memorial Hospital, Fu -Shin Rd. No. 5, Kwei - Shan, Tao-Yuan 333, Taiwan
Tel: 886-3-3281200 ext 8162
Fax: 886-3-3271192
E-mail: yeongshinn@cgmh.org.tw

Wei-Jan Chen, MD, PhD
The Cardiovascular Department, Chang Gung Memorial Hospital, Fu -Shin Rd. No. 5, Kwei - Shan, Tao-Yuan 333, Taiwan
Tel: 886-3-3281200 ext 8162
Fax: 886-3-3271192
E-mail: wjchen@cgmh.org.tw

Figure 2B

CD31

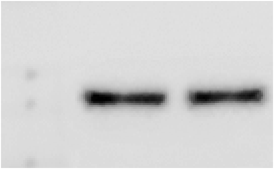

eNOS

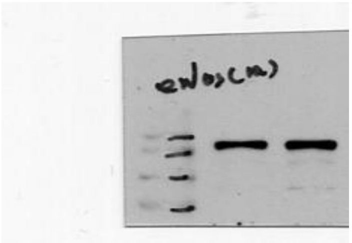

SMA

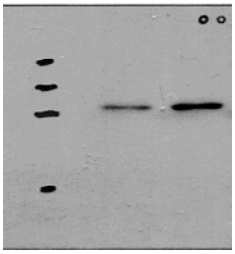

GAPDH

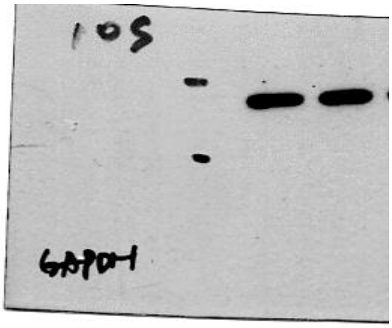

Figure 2F

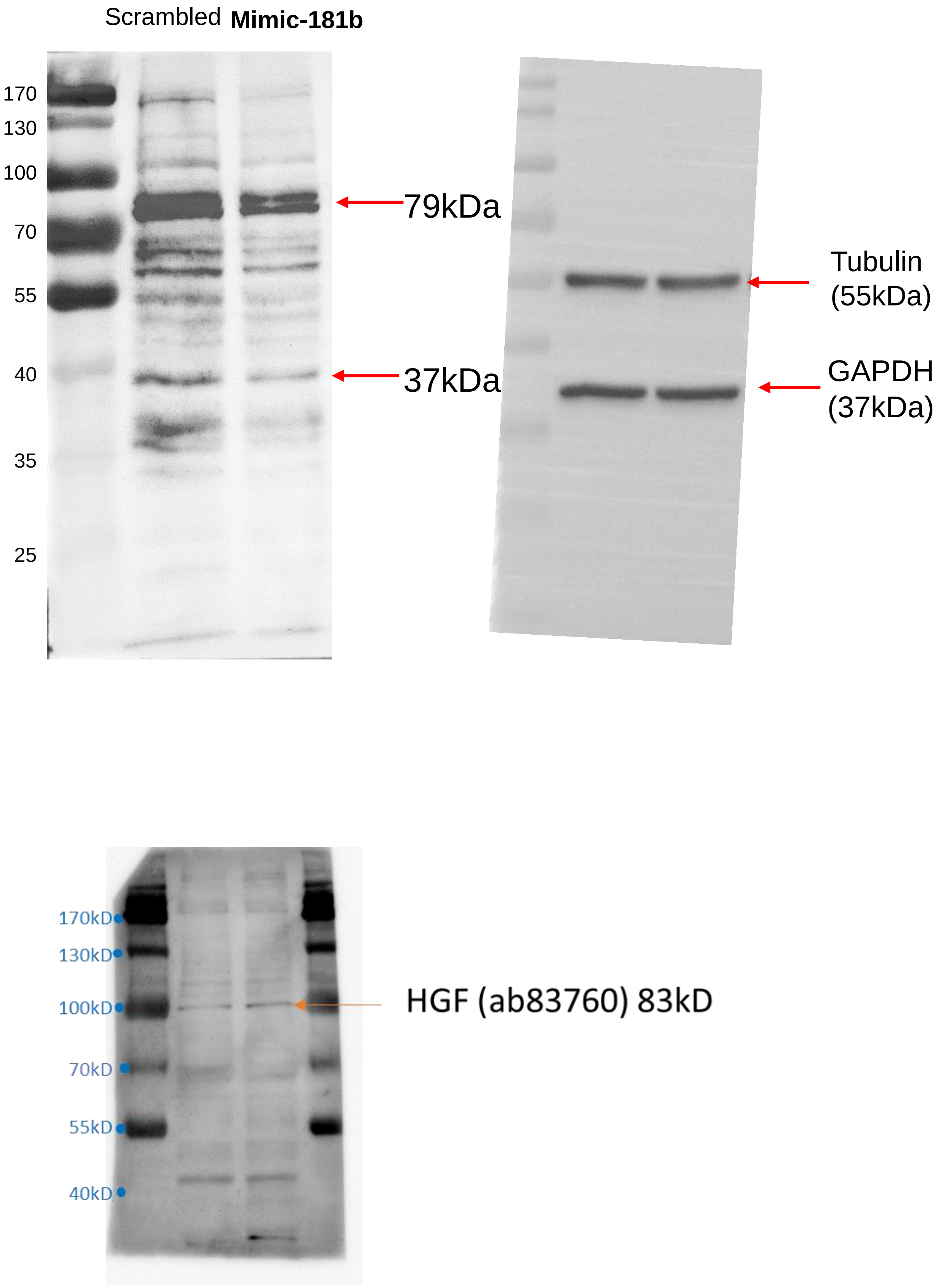

Scrambled
Mimic-181b
170
130
100
70
55
40
35
25
79kDa
37kDa
Tubulin (55kDa)
GAPDH (37kDa)
170kD
130kD
100kD
70kD
55kD
40kD
HGF (ab83760) 83kD

Figure 4A

Twist (26kD)

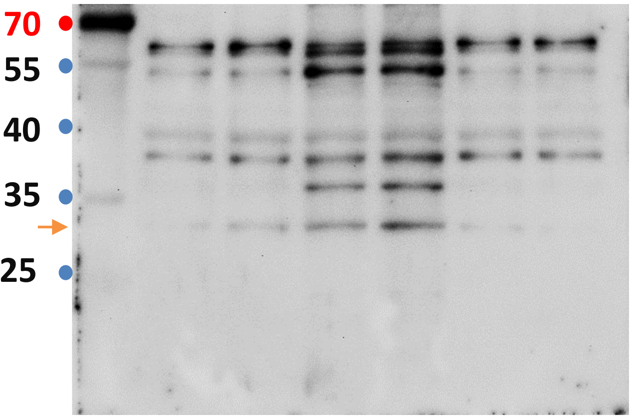

GAPDH(37kD)

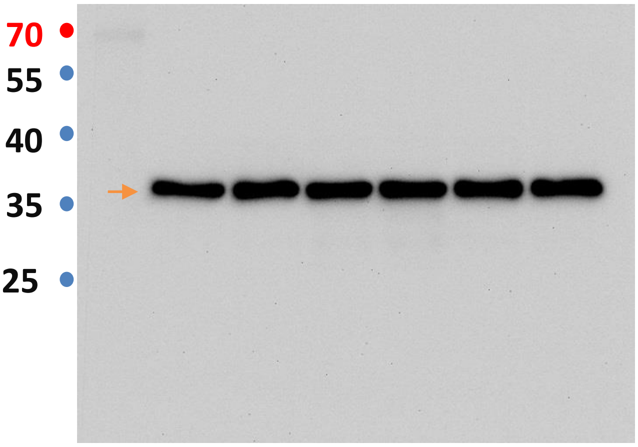

Sema3A

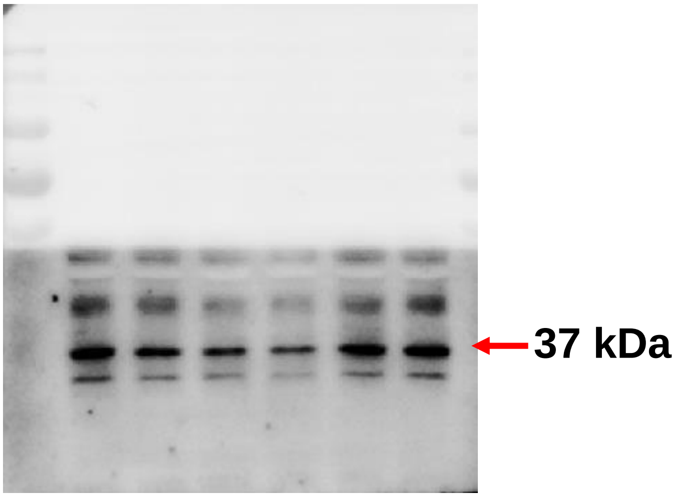

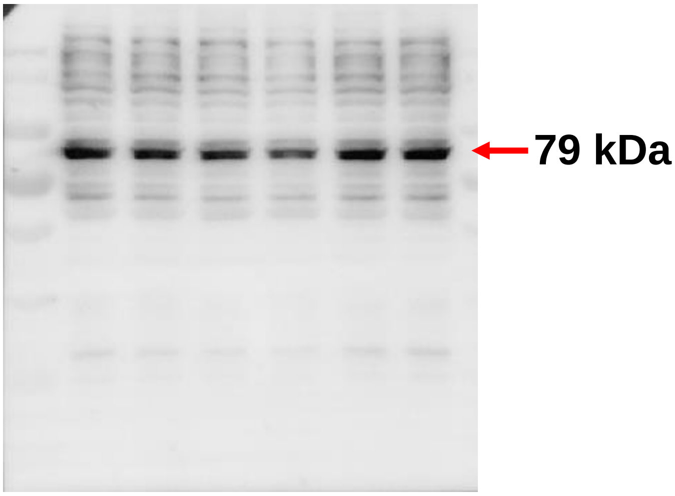

SMA (42kD)

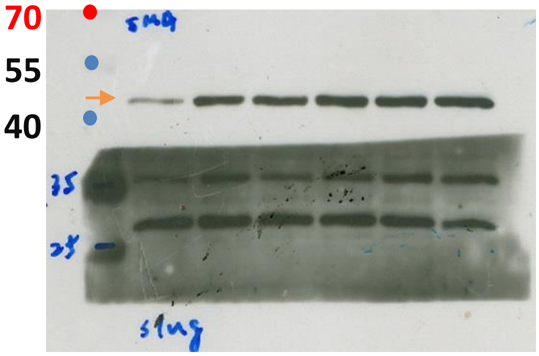

Figure 4A

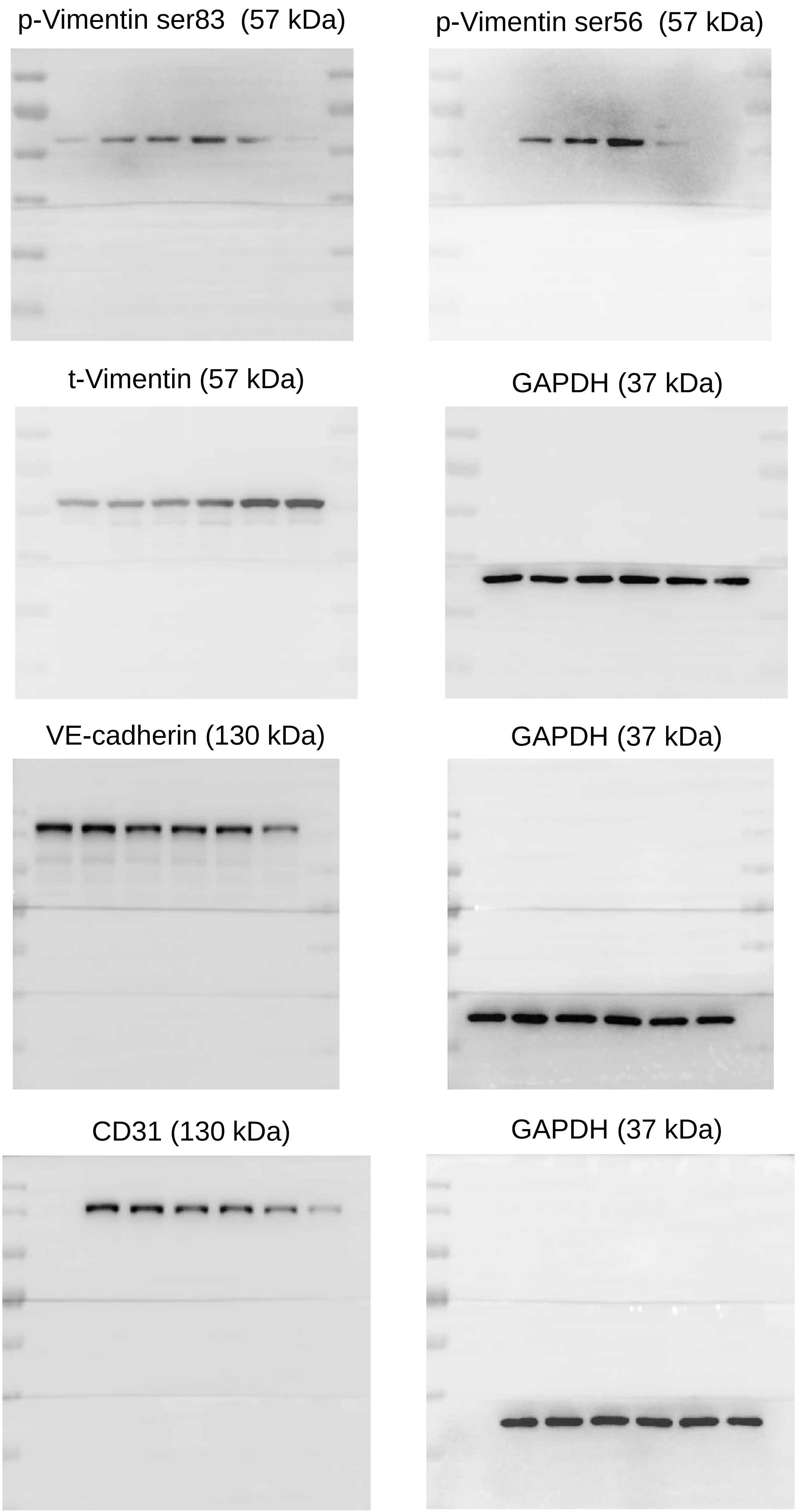
p-Vimentin ser83 (57 kDa)
p-Vimentin ser56 (57 kDa)
t-Vimentin (57 kDa)
GAPDH (37 kDa)
VE-cadherin (130 kDa)
GAPDH (37 kDa)
CD31 (130 kDa)
GAPDH (37 kDa)

Figure 4A

Collagen I (120 kDa)

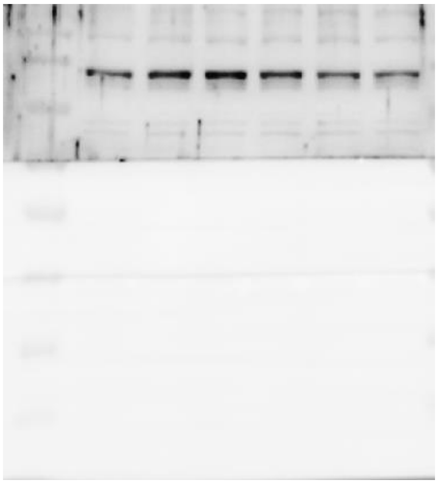

GAPDH (37 kDa)

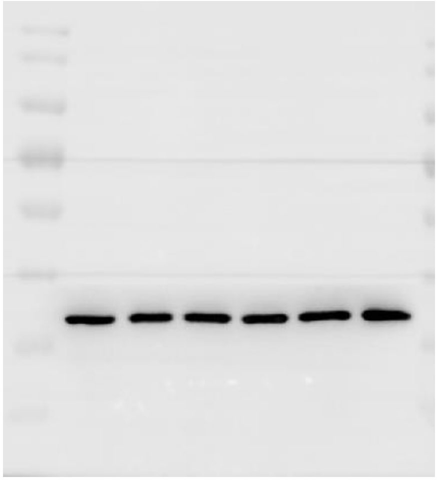

Snail (29 kDa)

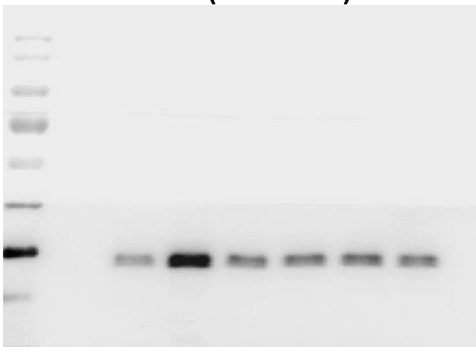

GAPDH (37 kDa)

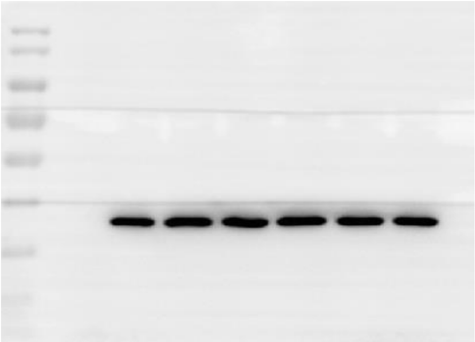

Slug (30 kDa)

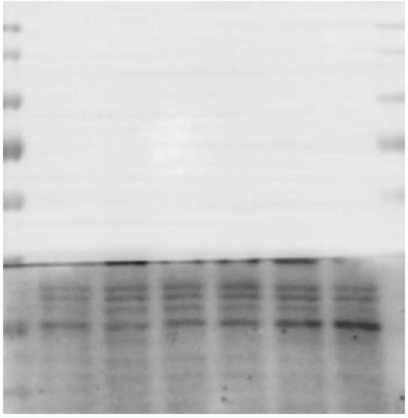

GAPDH (37 kDa)

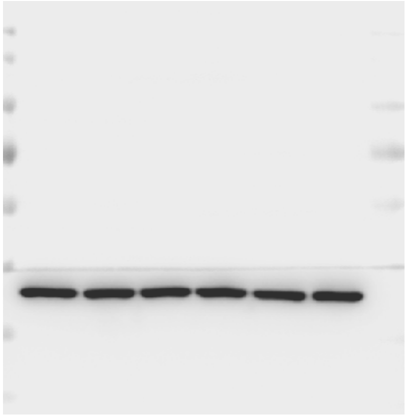

SM22α (22 kDa)

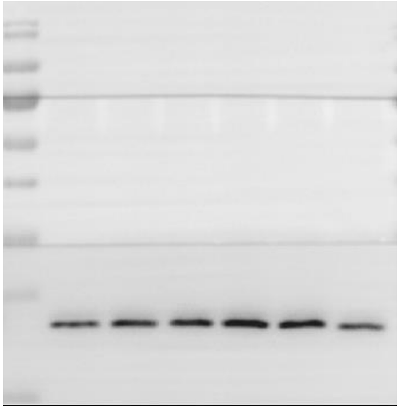

GAPDH (37 kDa)

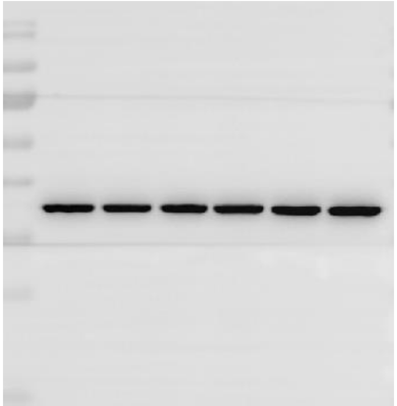

Figure 4B

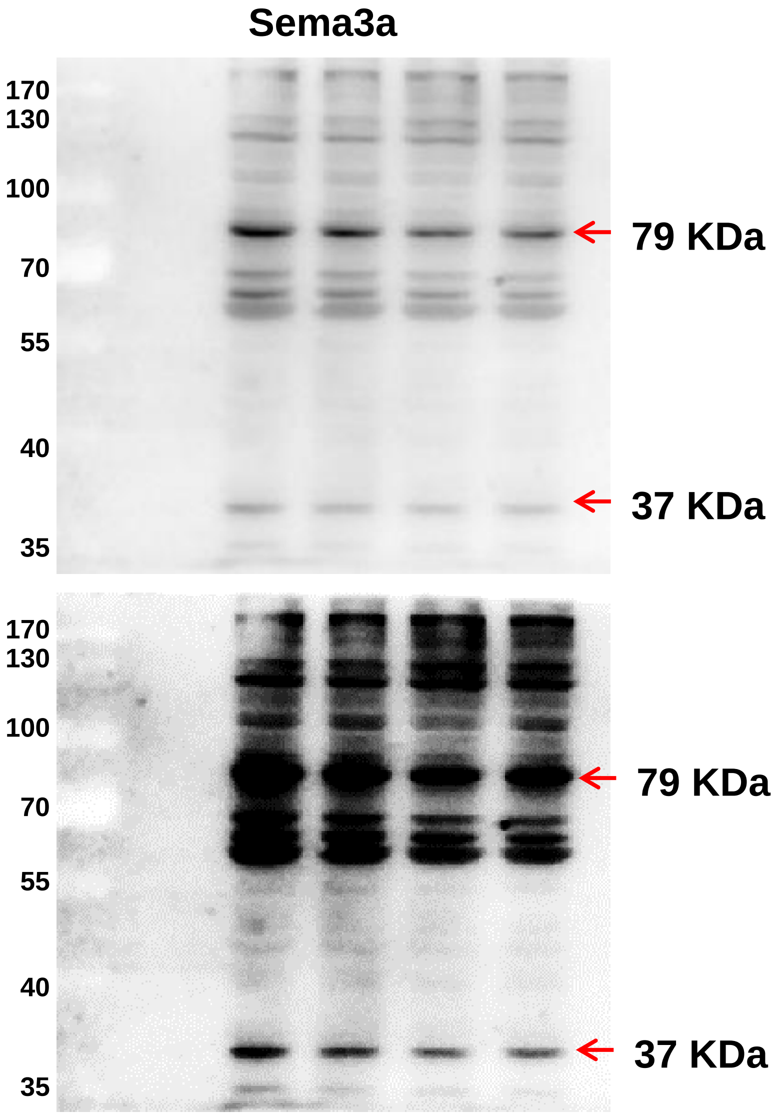
Sema3a
170
130
100
70
55
40
35
79 KDa
37 KDa
170
130
100
70
55
40
35
79 KDa
37 KDa

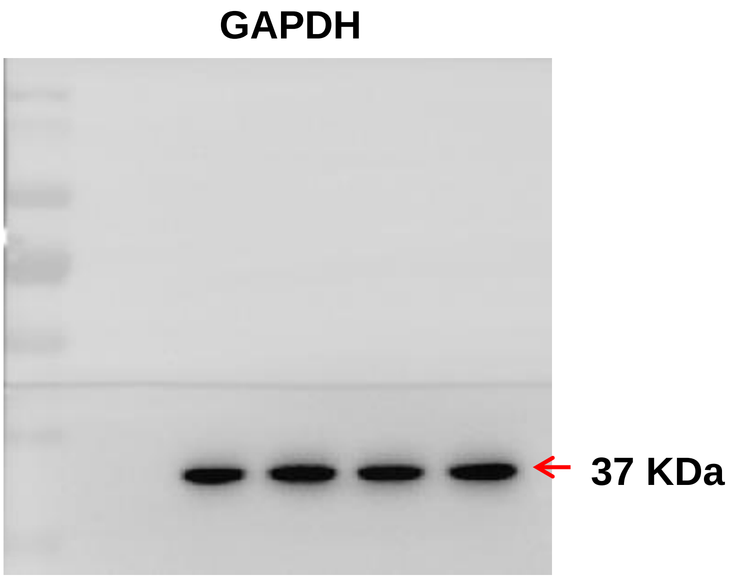
GAPDH
37 KDa

Figure 4B

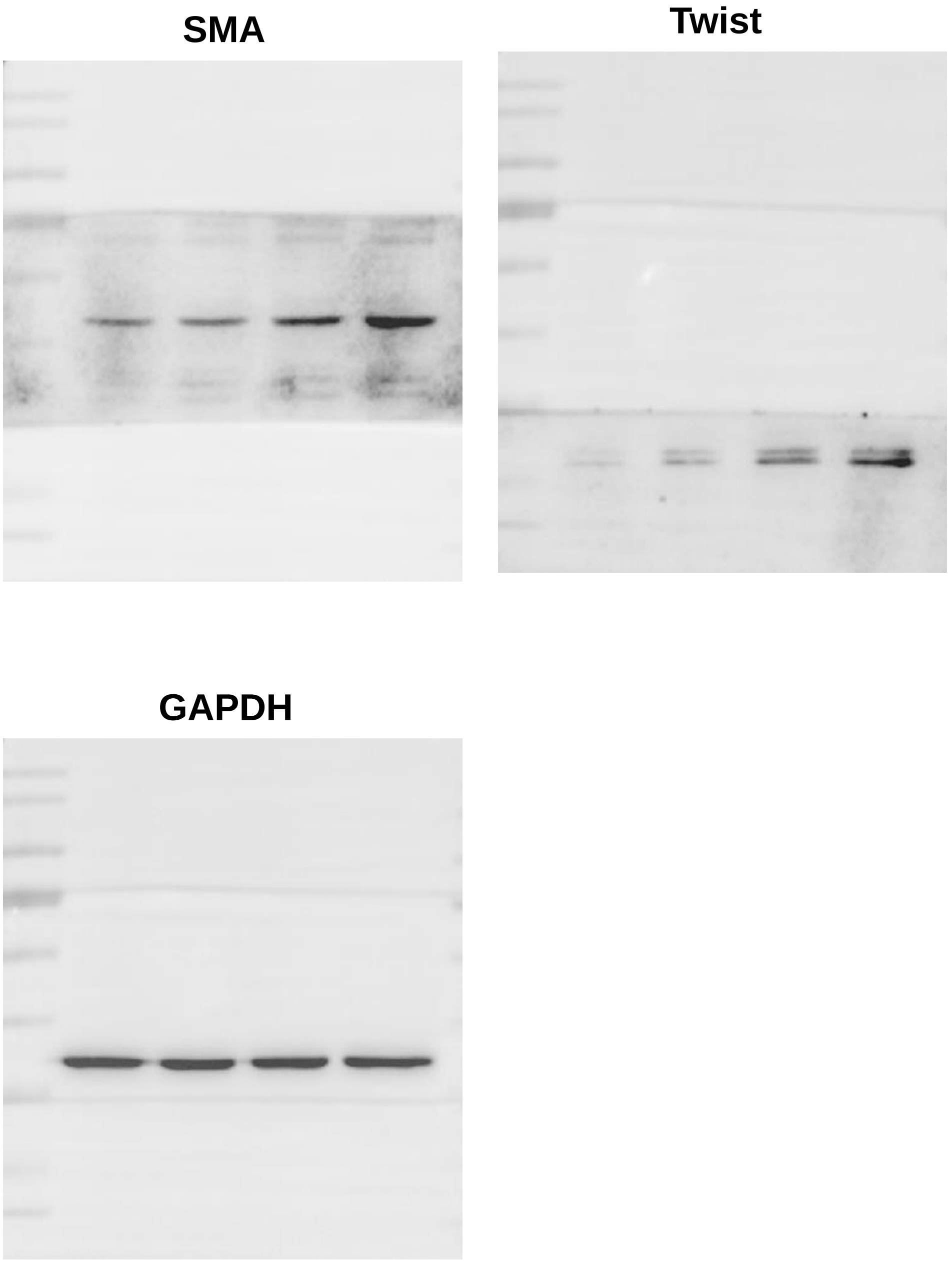

Figure 4C

PageRuler™ Prestained Protein Ladder, 10 to 180 kDa
Catalog number: 26616

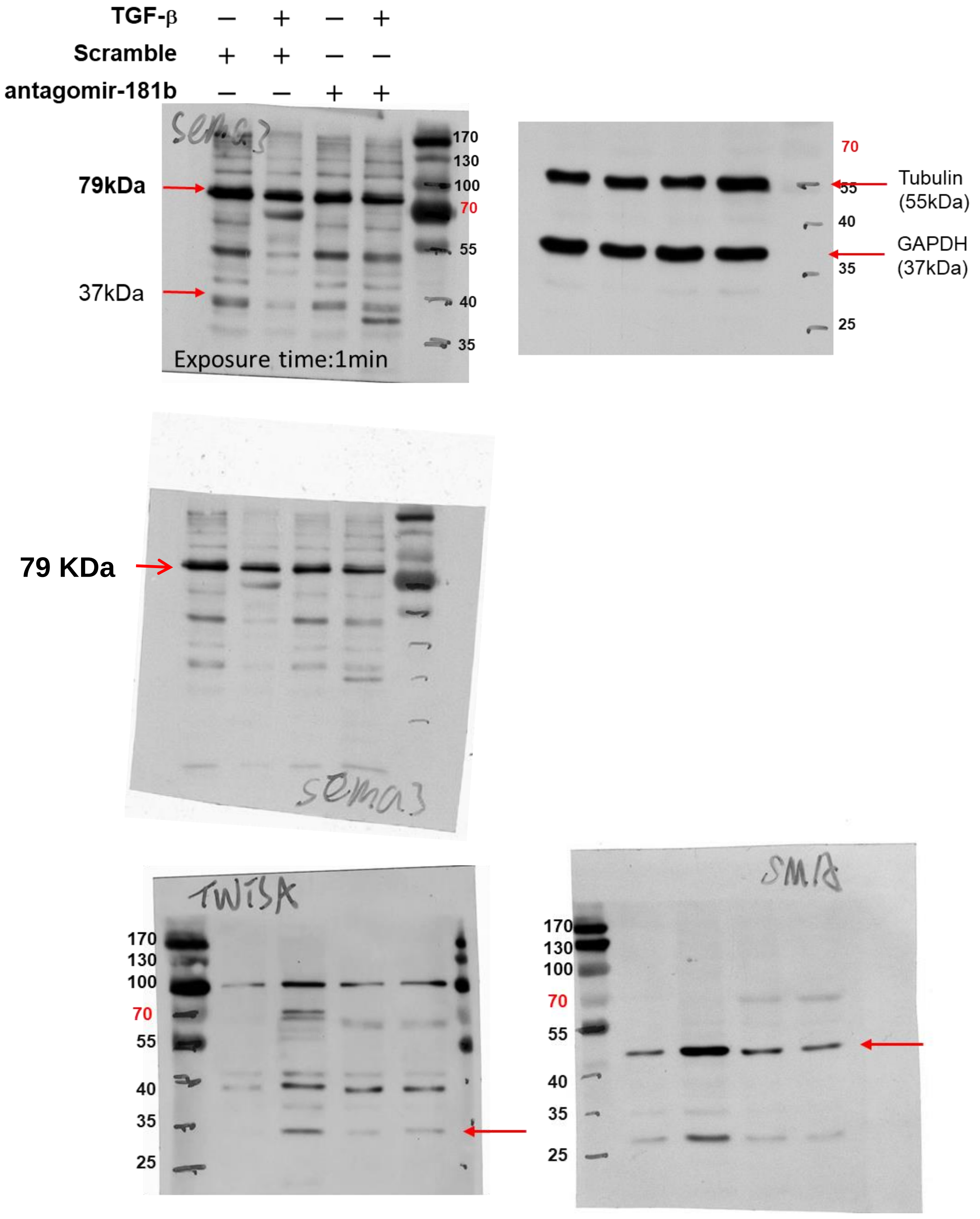

Figure 4G

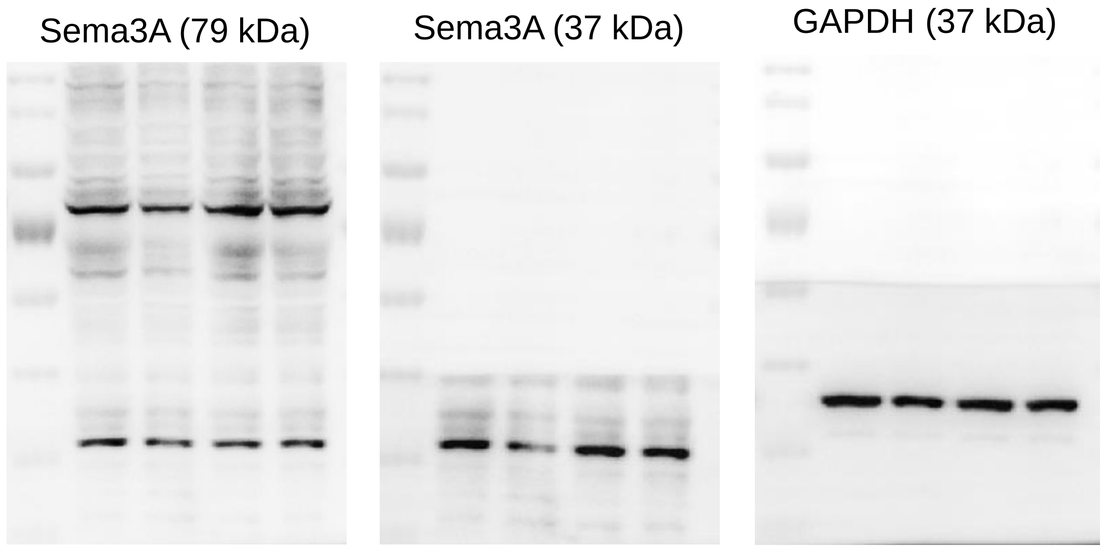

Figure 4H

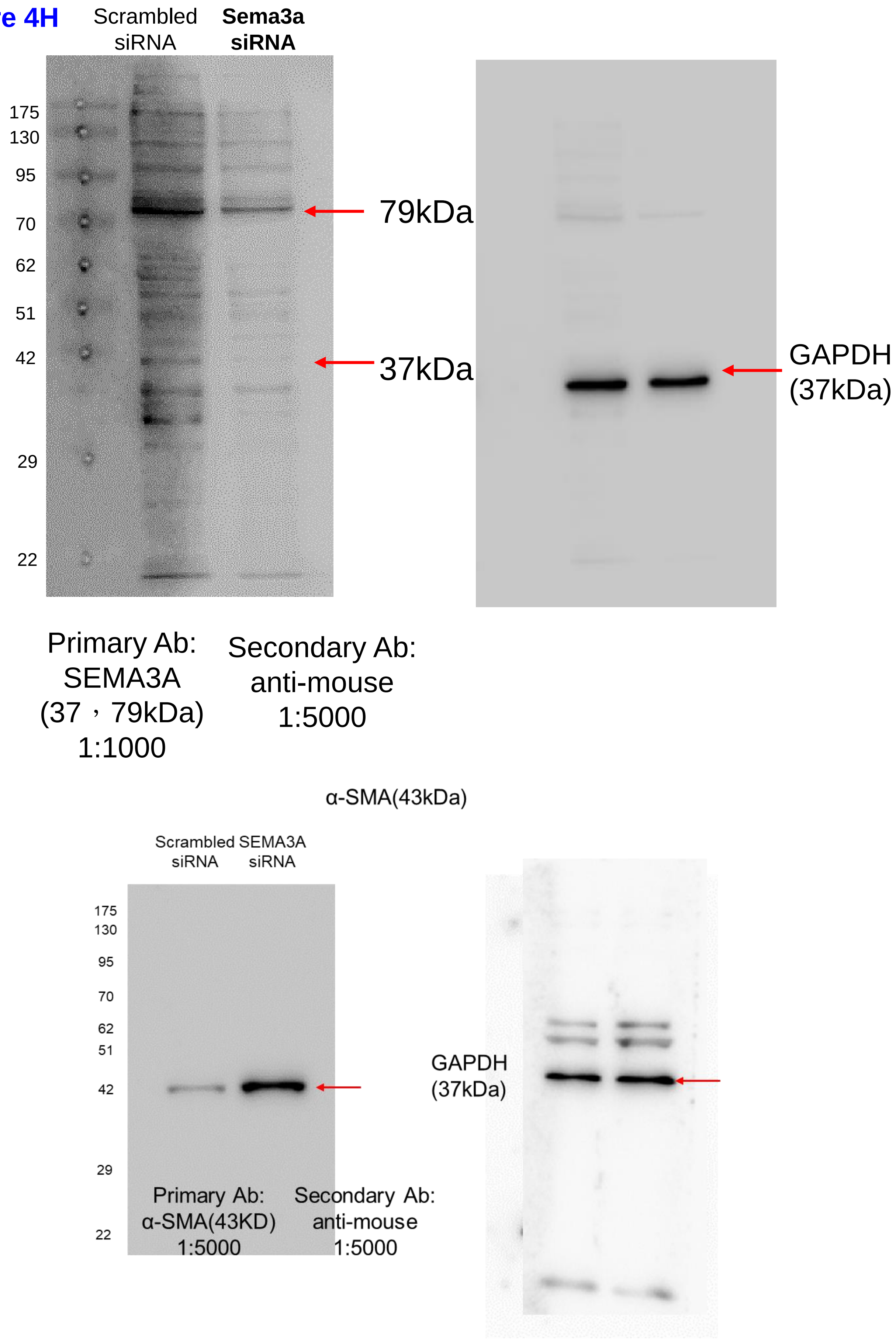

Scrambled siRNA
Sema3a siRNA
175
130
95
70
62
51
42
29
22
79kDa
37kDa
GAPDH (37kDa)
Primary Ab: SEMA3A (37，79kDa) 1:1000
Secondary Ab: anti-mouse 1:5000
α-SMA(43kDa)
Scrambled siRNA
SEMA3A siRNA
175
130
95
70
62
51
42
29
22
Primary Ab: α-SMA(43KD) 1:5000
Secondary Ab: anti-mouse 1:5000
GAPDH (37kDa)

Figure 4H

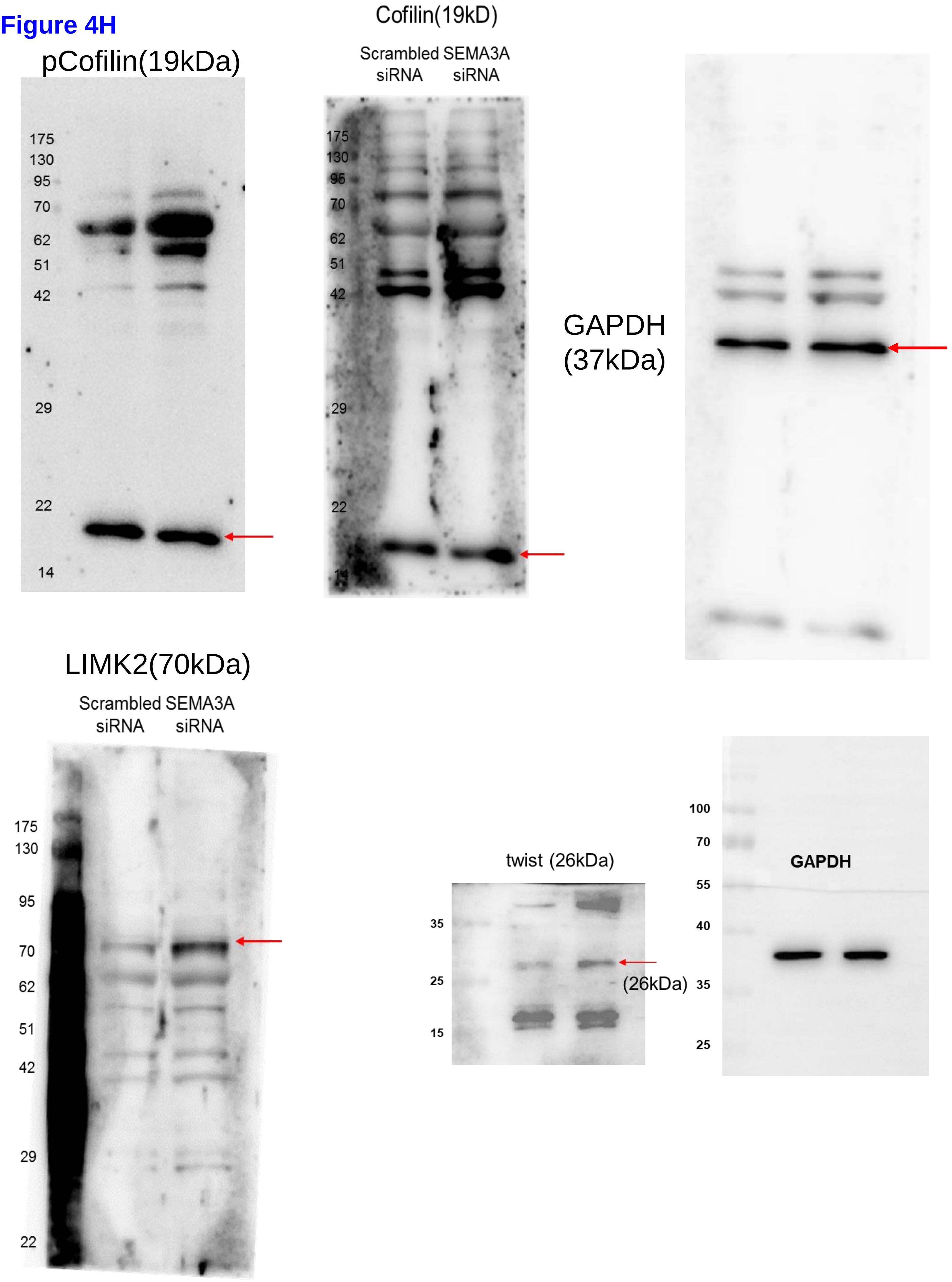
pCofilin(19kDa)
175
130
95
70
62
51
42
29
22
14
Cofilin(19kD)
Scrambled siRNA
SEMA3A siRNA
175
130
95
70
62
51
42
29
22
14
GAPDH (37kDa)
LIMK2(70kDa)
Scrambled siRNA
SEMA3A siRNA
175
130
95
70
62
51
42
29
22
twist (26kDa)
35
25
15
(26kDa)
100
70
55
40
35
25
GAPDH

Figure 5A

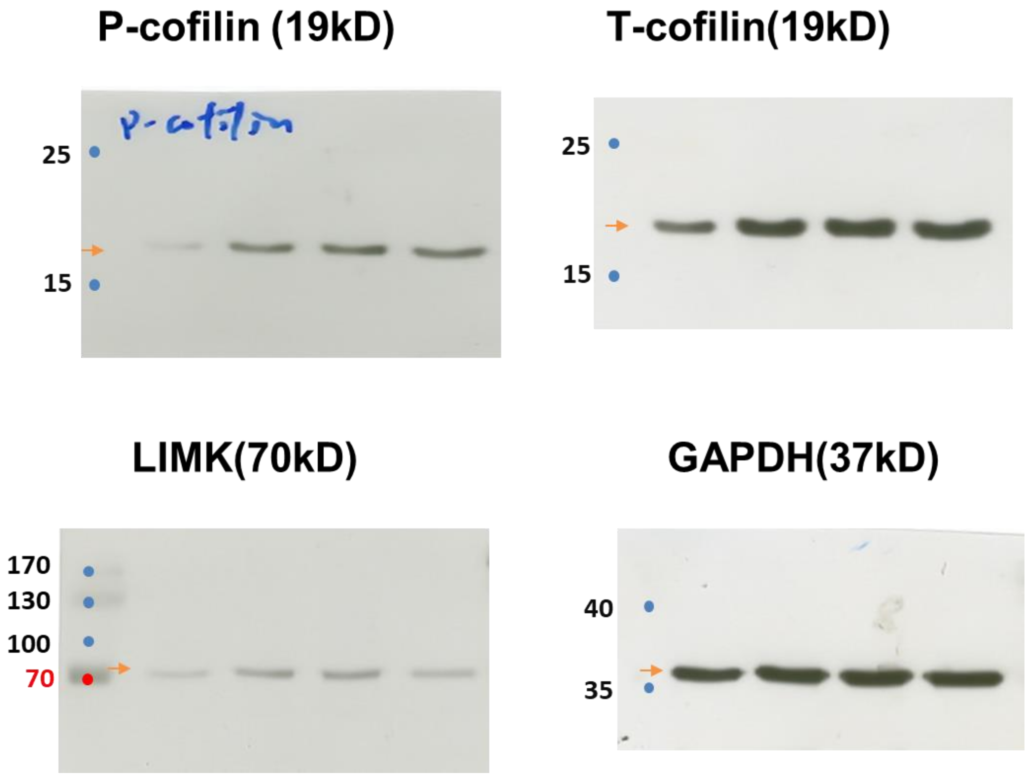
P-cofilin (19kD)
T-cofilin(19kD)
P-cofilin
25
15
25
15
LIMK(70kD)
GAPDH(37kD)
170
130
100
70
40
35

Figure 5B

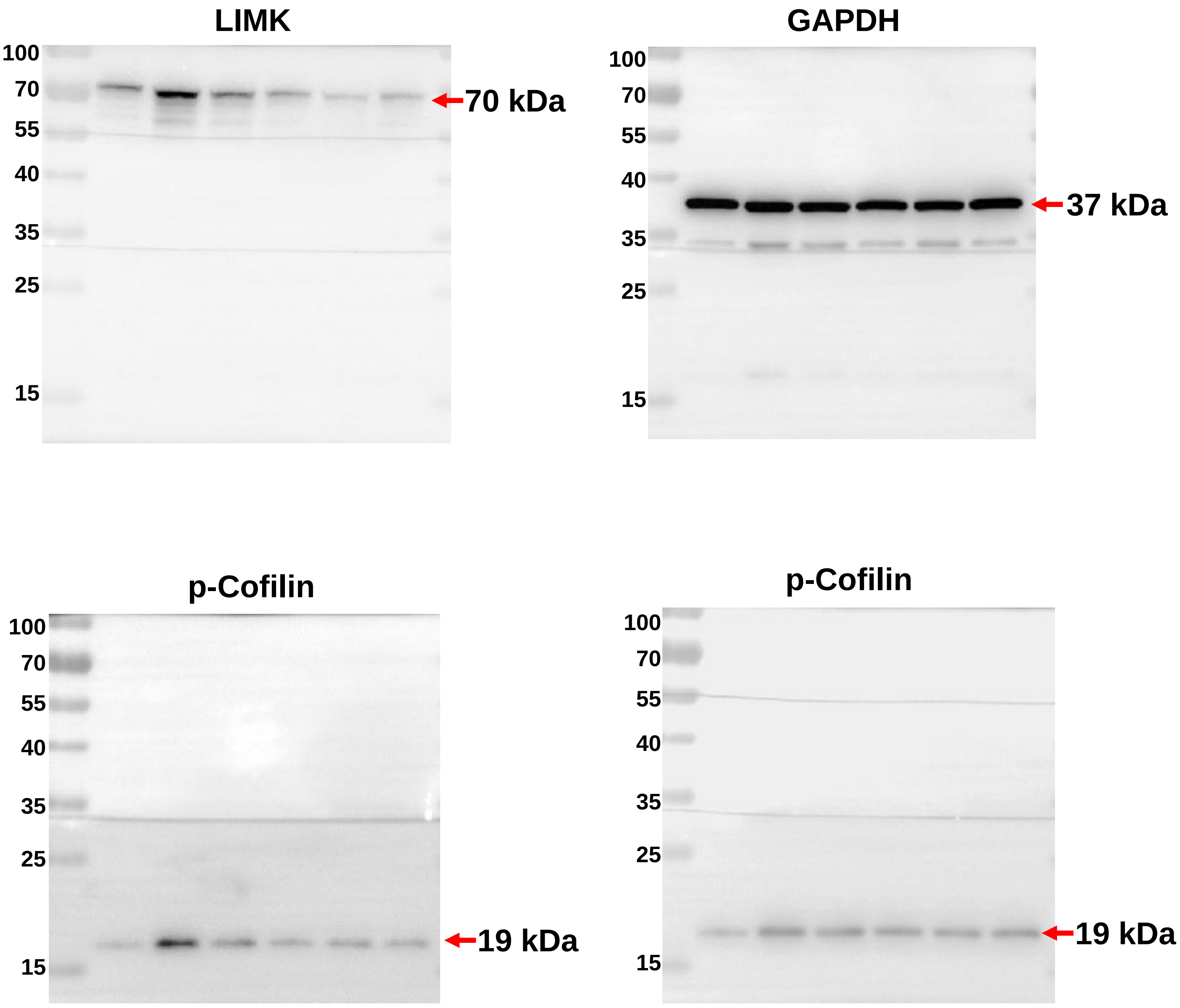
LIMK
100
70
55
40
35
25
15
70 kDa
GAPDH
100
70
55
40
35
25
15
37 kDa
p-Cofilin
100
70
55
40
35
25
15
19 kDa
p-Cofilin
100
70
55
40
35
25
15
19 kDa

Figure 5B

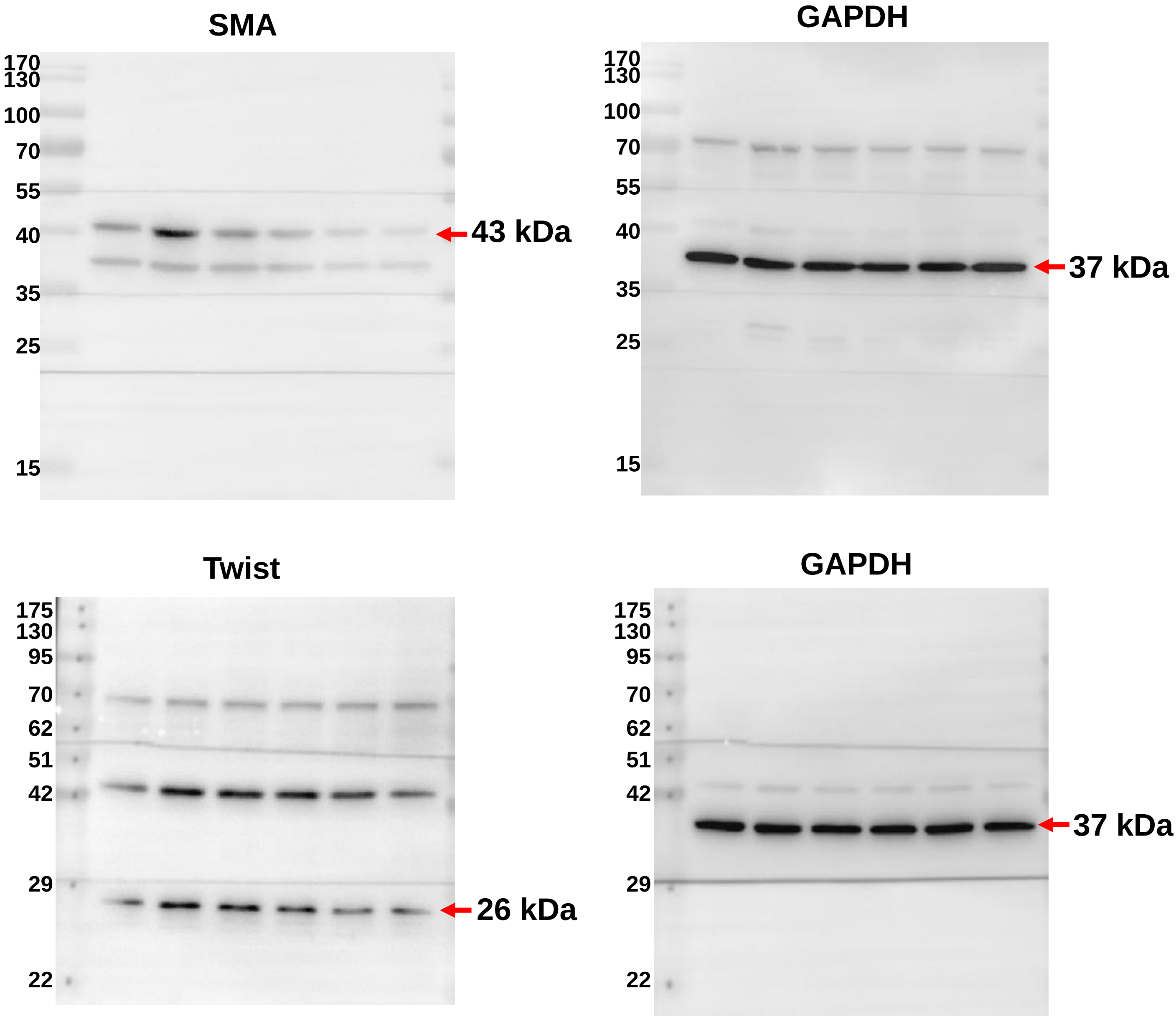

SMA
170
130
100
70
55
40
35
25
15
43 kDa
GAPDH
170
130
100
70
55
40
35
25
15
37 kDa
Twist
175
130
95
70
62
51
42
29
22
26 kDa
GAPDH
175
130
95
70
62
51
42
29
22
37 kDa

Figure 5B

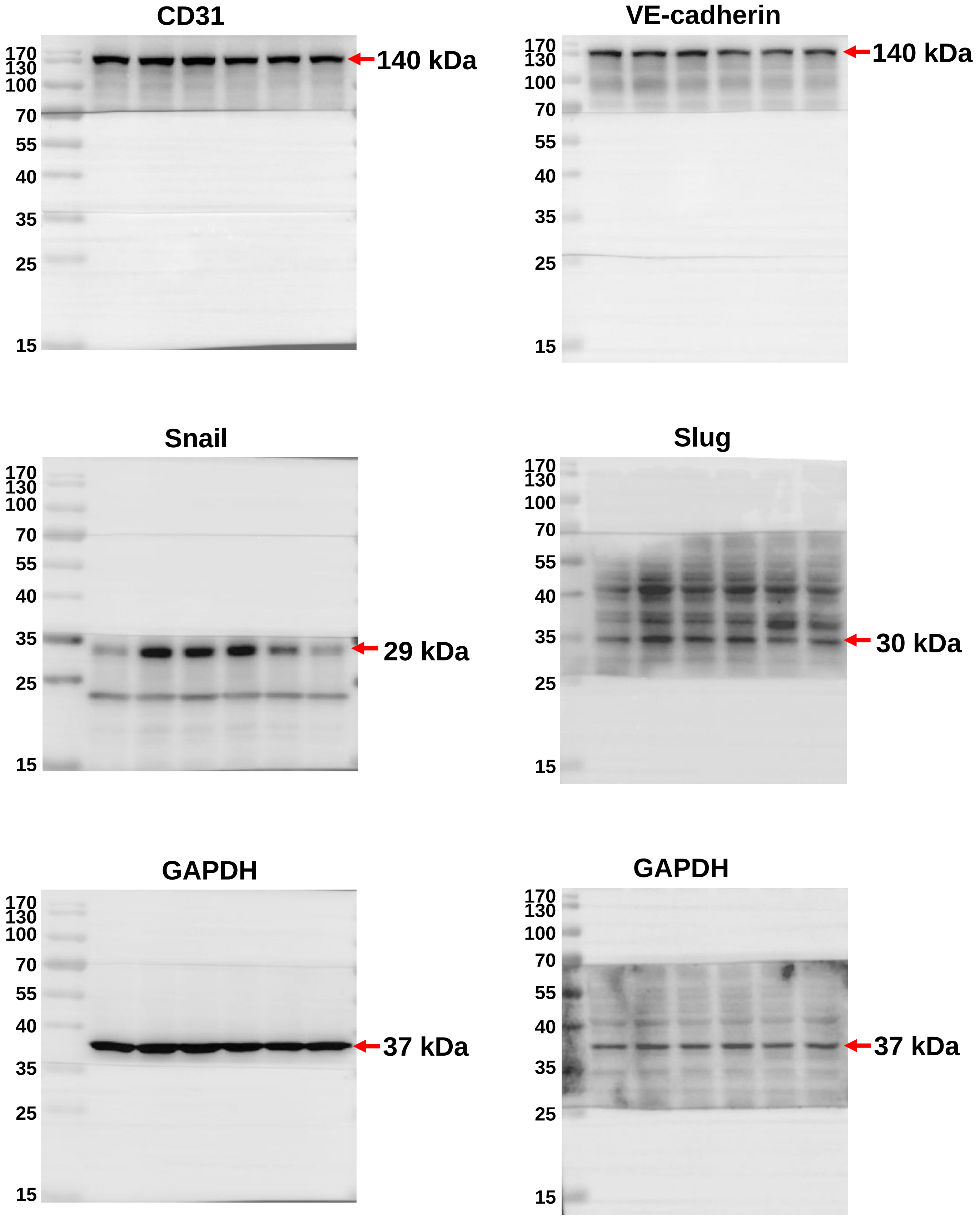
CD31
170
130
100
70
55
40
35
25
15
140 kDa
VE-cadherin
170
130
100
70
55
40
35
25
15
140 kDa
Snail
170
130
100
70
55
40
35
25
15
29 kDa
Slug
170
130
100
70
55
40
35
25
15
30 kDa
GAPDH
170
130
100
70
55
40
35
25
15
37 kDa
GAPDH
170
130
100
70
55
40
35
25
15
37 kDa

Figure 5B

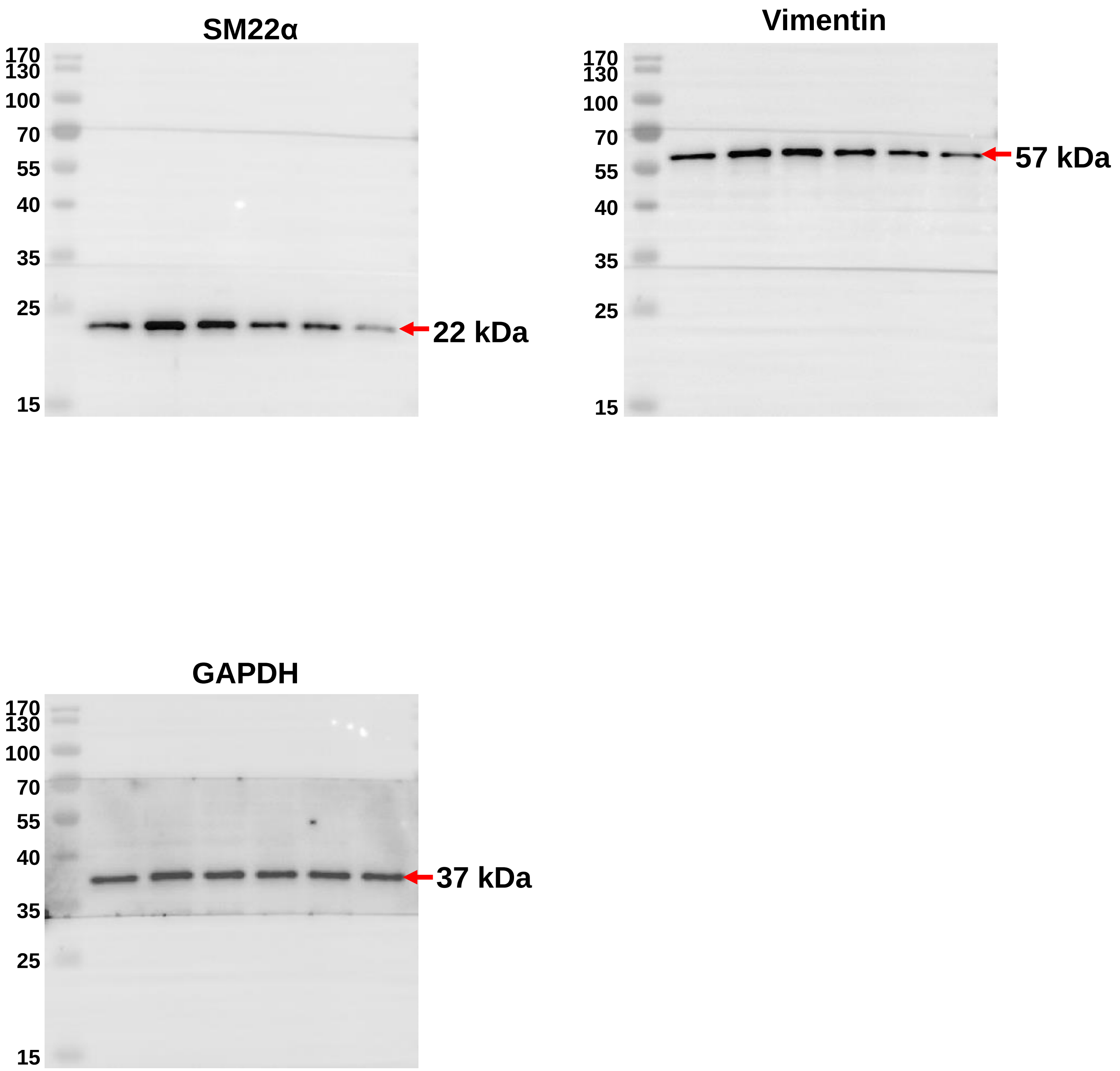
SM22α
170
130
100
70
55
40
35
25
15
22 kDa
Vimentin
170
130
100
70
55
40
35
25
15
57 kDa
GAPDH
170
130
100
70
55
40
35
25
15
37 kDa

Figure 6E

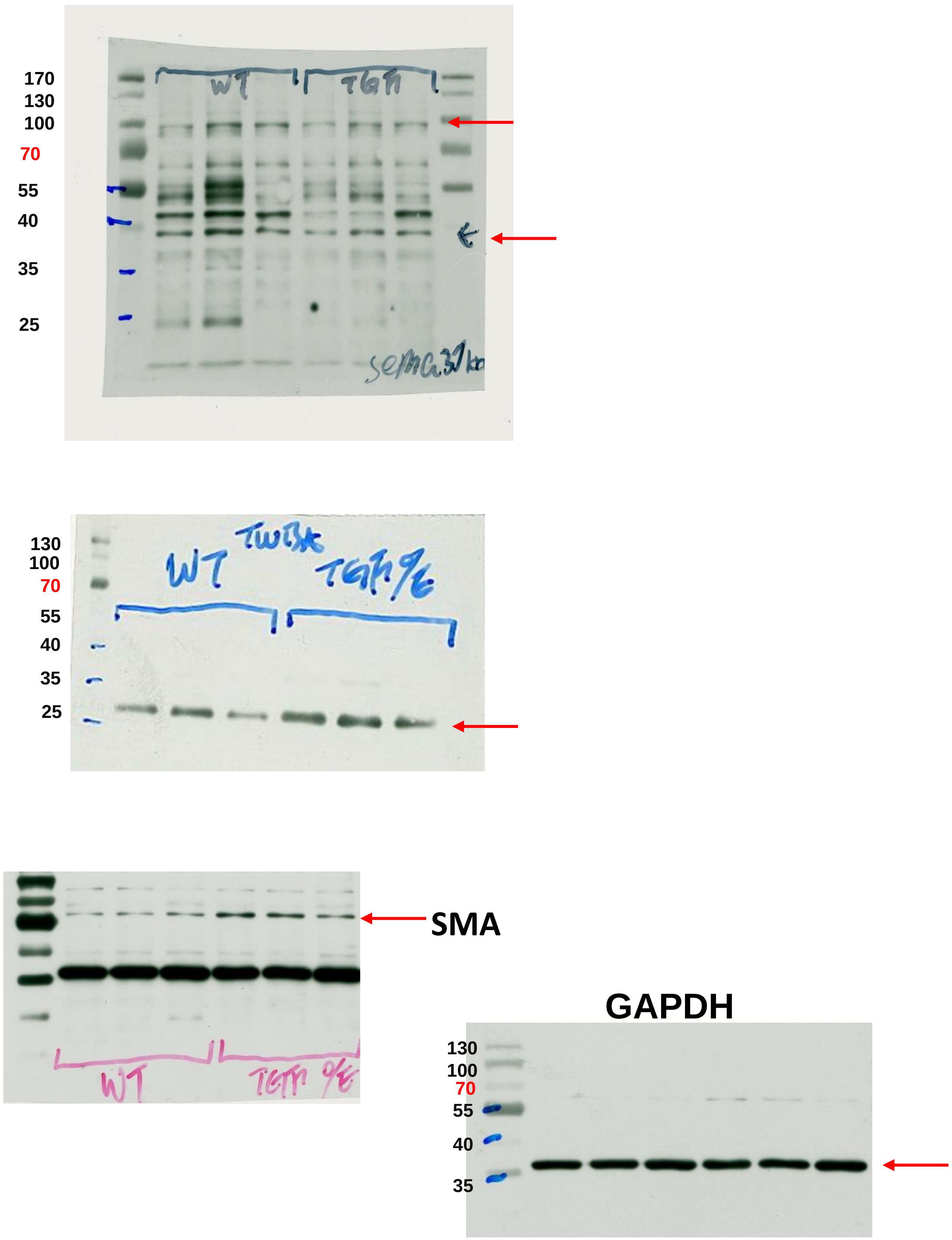

170
130
100
70
55
40
35
25
130
100
70
55
40
35
25
WT
SMA
GAPDH
130
100
70
55
40
35

Figure 7H.

Sema3A (79 kDa)

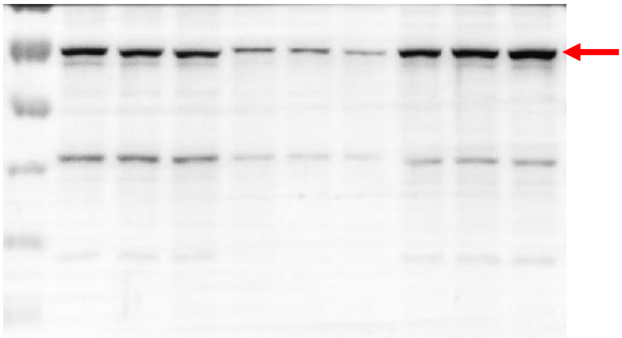

Sema3A (37 kDa)

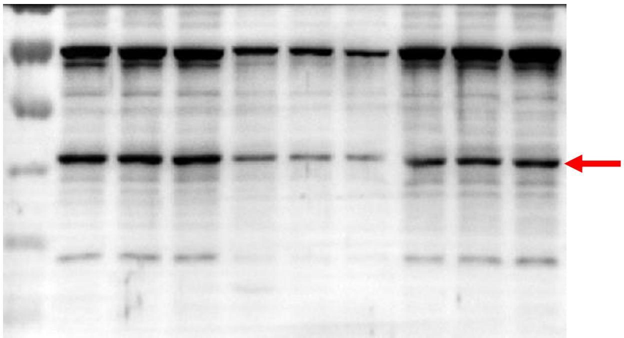

VE-cadherin (130 kDa)

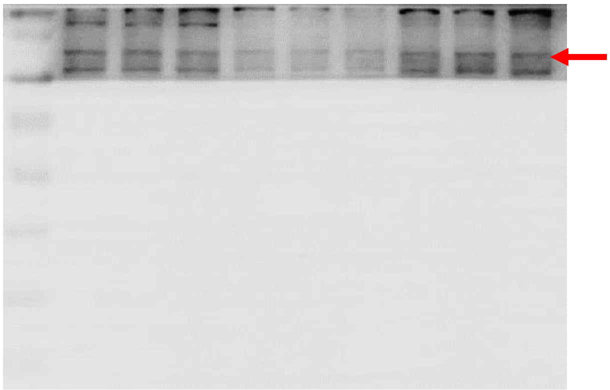

CD31 (130 kDa)

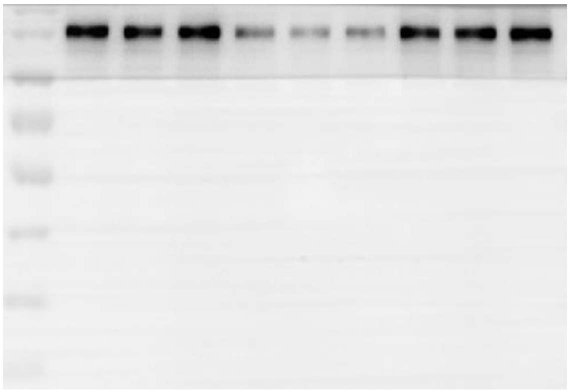

GAPDH (37 kDa)

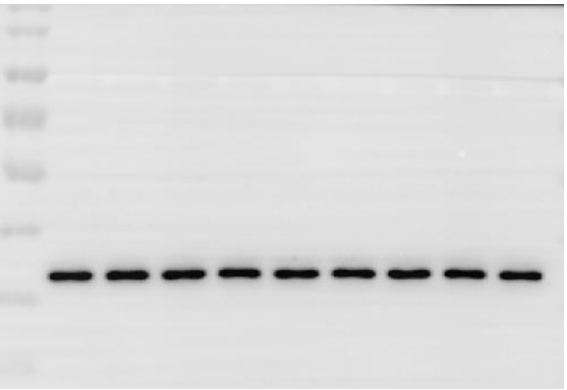

Figure 7H.

t-Vimentin (57 kDa)

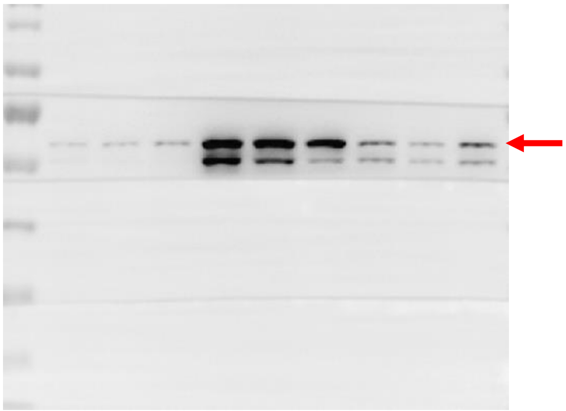

Twist (26kD)

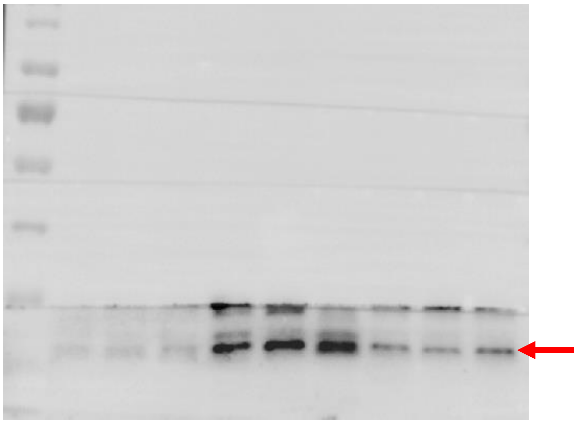

SMA (43kD)

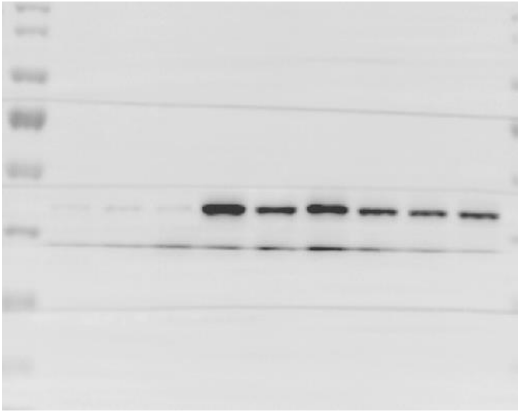

SM22α (22 kDa)

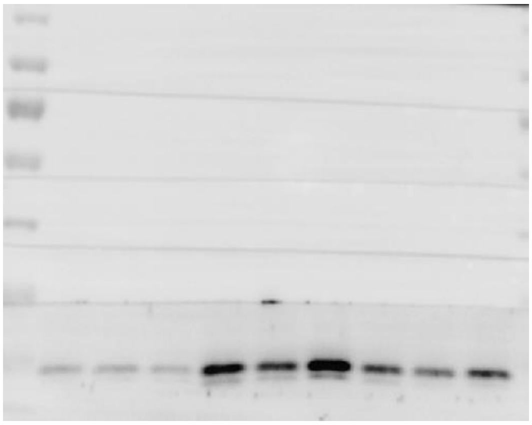

GAPDH (37 kDa)

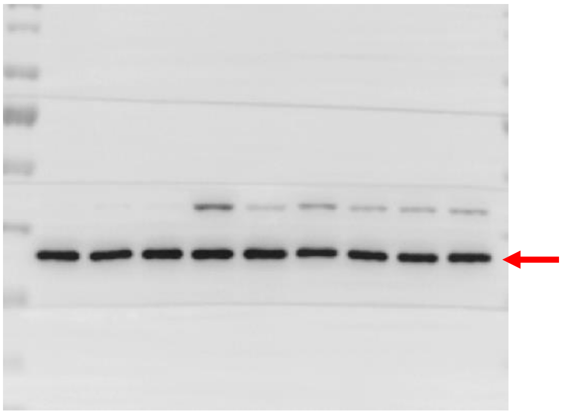

Figure 7H.

Snail (29 kDa)

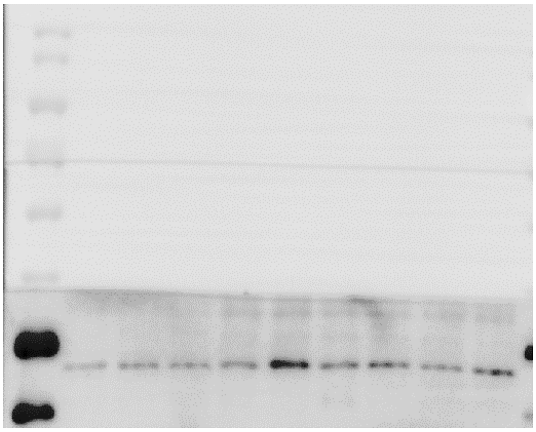

Slug (30 kDa)

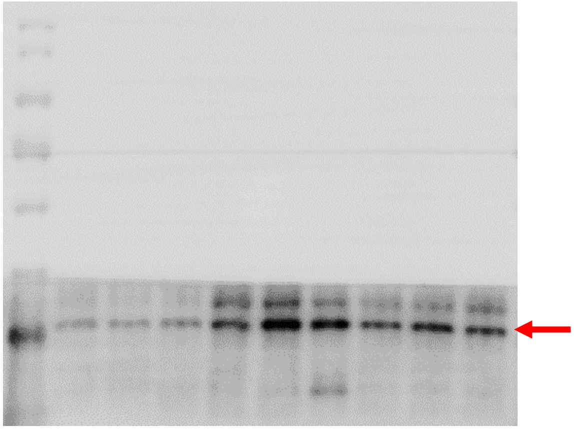

GAPDH (37 kDa)

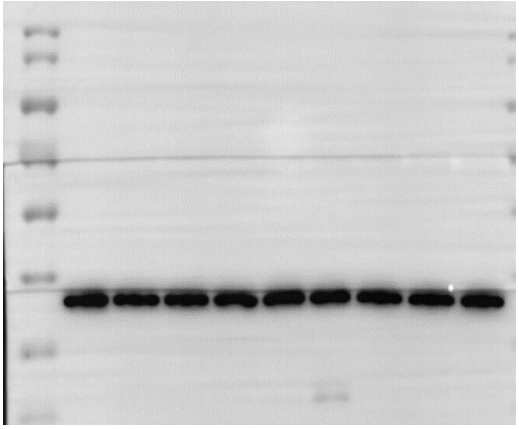

Collagen I (120 kDa)

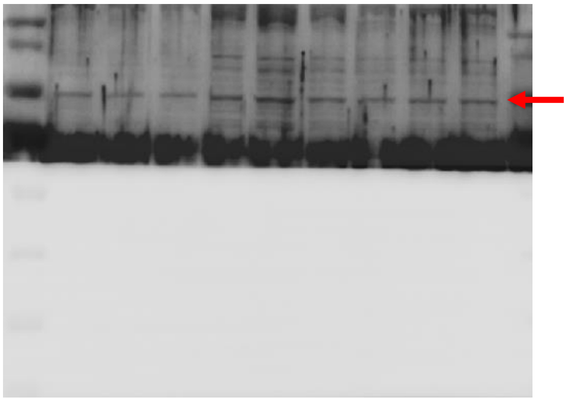

GAPDH (37 kDa)

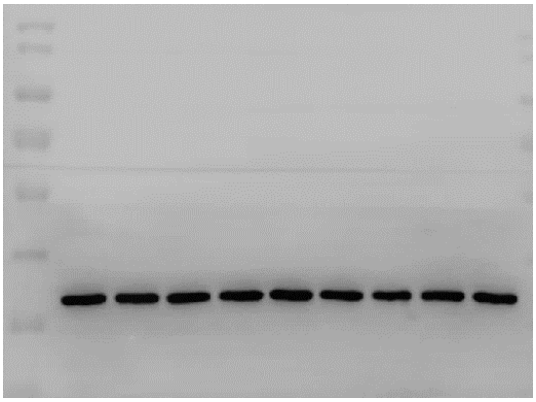

Figure8D

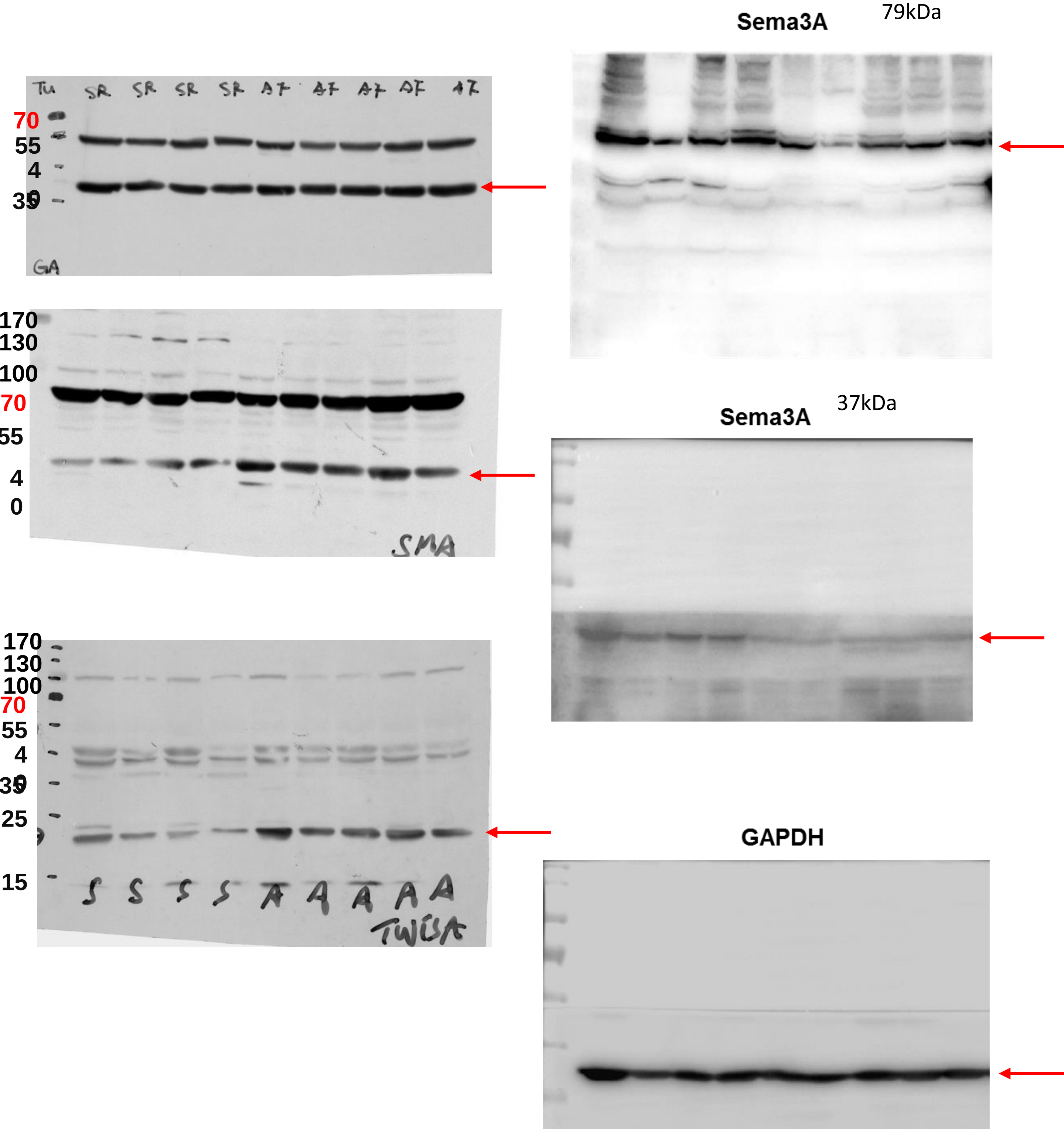
Sema3A
79kDa
Sema3A
37kDa
GAPDH
70
55
170
130
100
70
55
170
130
100
70
55
25
15

Supplemental Figure 2C

CD31

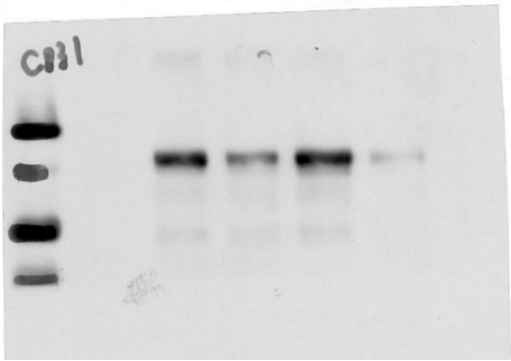

eNOS

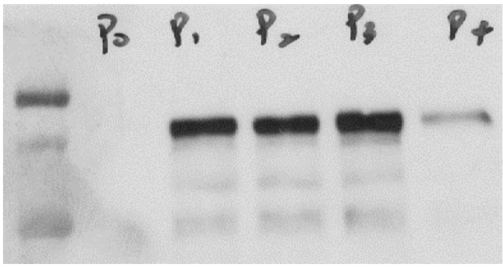

tubulin

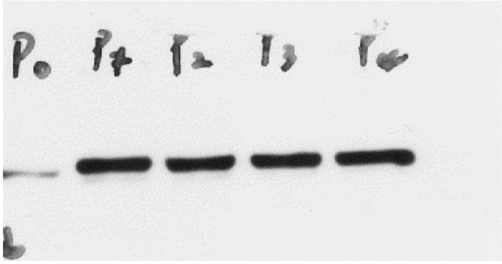

Supplement Figure 4.

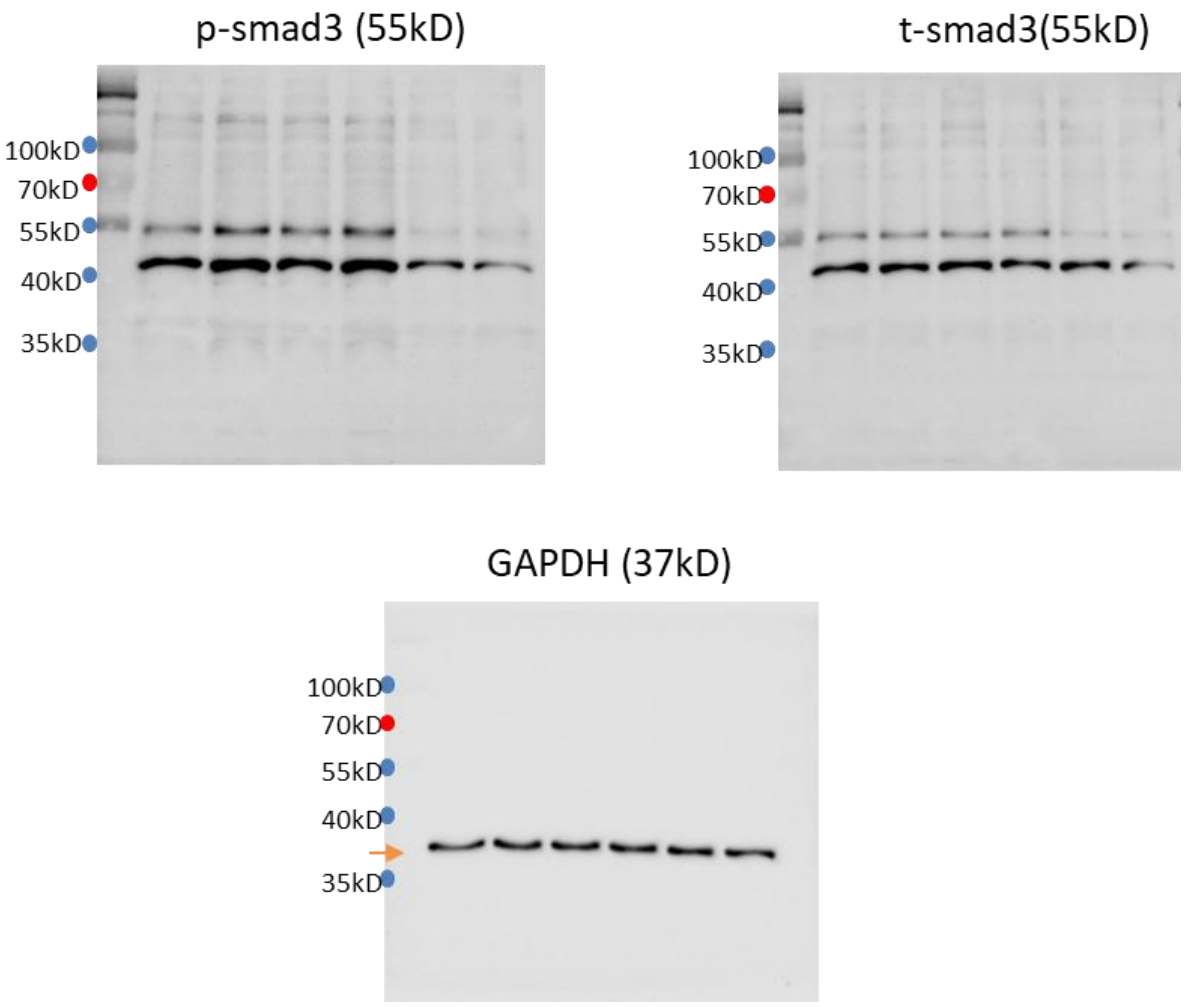

Supplement: Supplemental data [file jci-132-142548-s213.pdf]
